# Ambivalence in pregnancy intentions: The effect of quality of care and context among a cohort of women attending family planning clinics in Kenya

**DOI:** 10.1371/journal.pone.0190473

**Published:** 2018-01-09

**Authors:** Eliud Wekesa, Ian Askew, Timothy Abuya

**Affiliations:** 1 School of Humanities and Social Sciences, South Eastern Kenya University, Kitui, Kenya; 2 Department of Reproductive Health, Population Council, Nairobi, Kenya; 3 Department of Reproductive Health and Research, World Health Organization, Geneva, Switzerland; Public Library of Science, UNITED KINGDOM

## Abstract

**Context:**

Ambivalence in pregnancy intentions is well-documented in sub-Saharan African (SSA) settings and has been associated with inconsistent use of contraception, thereby exposing women using contraception to the possibility of unintended pregnancies. A better understanding of the potential role for client counseling interventions in enabling women to achieve their pregnancy intentions is essential for aiding program efforts to reduce unintended pregnancies.

**Objective:**

To measure ambivalence in pregnancy intentions longitudinally and determine its association with the quality of care received, controlling for demographic, socio-economic and contextual factors among a cohort of family planning (FP) clients in Kenya.

**Methods:**

This paper uses data drawn from a prospective cohort study of FP clients to investigate the relationship between the quality of care received during FP service delivery and the decisiveness of their pregnancy intentions over time. The study tests the hypothesis that higher quality of care enables women to be less ambivalent about their pregnancy intentions. Binary logistic regression with random effects and multinomial logistic regression were used to assess the predictive effect of the quality of care received by a woman on the decisiveness or ambivalence of her pregnancy intentions, and on any shifts in ambivalence over time, controlling for background characteristics. The study recruited 1,957 women aged 15–49 years attending twelve family planning clinics in four counties in Central Kenya; of these, 1,053 women were observed for four rounds of data collection over a period of 24 months and form the sample for analysis.

**Findings:**

A substantial proportion (43%) of women expressed ambivalence about their intentions to become pregnant at some point during the study period, while over half (57%) remained unequivocal throughout the study. Almost one third of women (31%) shifted from being unequivocal to ambivalent and 12% shifted from ambivalence to being unequivocal. Women experiencing higher quality of care have lower odds of ever expressing ambivalence and higher odds of remaining unequivocal over time, net of other factors. Quality of care was not associated with a shift in ambivalence over time.

**Conclusion:**

FP programs offering higher quality of care are likely to support women to be more decisive in their pregnancy intentions. Improving the quality of care can contribute to reduced ambivalence and consequently reduced likelihood of unintended pregnancy among contraceptive users. This study provides further evidence of the benefits gained through providing high quality services.

**Trial registration:**

ClinicalTrials.gov NCT01694862

## Introduction

Many policy statements and much research tend to treat pregnancy intentions as clear-cut dichotomous categories, that is, either intended or unintended [1, 2]. In most population surveys, pregnancy intendedness is derived from retrospective self-reporting by women about their last pregnancy or birth. For example, the demographic and health surveys (DHS) measure pregnancy intendedness through the following question: *At the time you became pregnant*, *did you want to become pregnant then*, *did you want to wait until later*, *or did you not want to have any (more) children at all*? [3]. A pregnancy is then classified as unintended if it is reported to have been mistimed (occurred earlier than planned) or unwanted (occurred when no more children were desired [3].

However, this classification does not reveal the complexity of factors influencing women’s expressions of their reproductive intentions [2, 4]. Pregnancy intentions can be complex, involving a range of emotional and psychological factors that are a product of individual intentions, and multiple interwoven social and economic influences, including community, partner and personal values about childbearing [2]. Qualitative studies show that many women hold attitudes and intentions about pregnancy that are ambivalent, apparently contradictory or poorly specified, and that these may vary over time [5]. Pregnancy ambivalence is defined as “unresolved or contradictory feelings about whether one wants to have a child at a particular moment” [6, 7]. Apparent discrepancies may exist between women’s stated pregnancy intentions and her reported happiness or unhappiness [5]. For example, studies in the United States have found that 29% of women interviewed expressed ambivalence about their last pregnancy [8] and that 45% of women and men expressed ambivalence about their pregnancy intentions [6]. In Africa, DHS data from Burkina Faso, Ghana and Kenya showed that at least a quarter of women (one third in Kenya) who expressed wanting to delay or limit their next birth also reported that a pregnancy in the next few weeks would not be a problem [9]. Therefore, estimated rates of unintended pregnancy that are based on intentions expressed through responses to such questions may not be accurate if women have ambivalent feelings towards wanting or avoiding pregnancy [10].

It is important to capture ambivalence in pregnancy intentions because studies show that women who express ambivalent feelings towards pregnancy may use less effective methods of contraception [1] and/or are inconsistent users of contraceptive methods [11, 12]. Understanding a woman’s pregnancy intentions when providing contraceptive services may help to ensure that she uses more effective methods and/or more consistently, thereby reducing the likelihood of unintended pregnancies [2].

Few studies of ambivalence have been undertaken in Sub-Saharan African settings, many of which are characterized by traditionally high fertility norms that are rapidly, but unevenly, changing as countries are entering into a demographic transition. At this point in the transition, many women and couples often face dilemmas of whether to conform to social and familial pressures for a large family, or to have a relatively small family to gain the economic, educational and benefits of raising fewer children. Moreover, the continuing, albeit slowly declining, prevalence of HIV/AIDS [13] may also influence fertility desires and pregnancy intentions of women and couples, especially those that know that they are living with HIV [14, 15]. Studies in Sub-Saharan Africa from the 1990s and early 2000s reported declines in fertility rates among women living with HIV/AIDS [16, 17], as well as lower fertility desires among HIV-infected individuals than their uninfected peers [18]. However, recent research conducted in the context of expanding availability of antiretroviral therapy (ART) and thus safer options for delivering healthy children has produced mixed results [18]. A recent study in Kenya, for example, found that fertility intentions among people living with HIV/AIDS are fraught with ambivalence and affected by age, sex, number of children, social support and household wealth [19].

Health care delivery in African settings is often bedeviled by poor quality of care. Evidence shows that improving the quality of reproductive health services that women receive improves client’s satisfaction and continued use of the services [20]. However, there is no empirical evidence on the effect of quality of care on fertility/pregnancy intentions. It is, therefore, hypothesized that improved quality of experiential clinical care helps women form unequivocal pregnancy intentions and subsequently enhances their ability to achieve their fertility goals or reproductive intentions.

## Materials and methods

### Study design, data sources and context

This study uses a subset of a dataset collected from a longitudinal quasi-experimental study–the ***Integra Initiative–***which was implemented from 2008 to 2013 to evaluate the benefits and costs of improving the quality of care provided to FP clients through integrating HIV services into existing and strengthening family planning services in Kenya. The dataset for this analysis is drawn from one particular component of the larger project: a cohort of family planning clients who were followed for 24 months to determine the effect of improving the quality of care through implementing a service integration intervention on women’s continuity of use of family planning and achievement of their stated pregnancy intentions. The full protocol for the *Integra* study is described elsewhere [21] (S1 Text).

The *Integra* intervention was originally implemented in six clinics, with a further six clinics selected through matching on specific characteristics (case load, type of health facility and level of care) to serve as comparison sites for a quasi-experimental design to test the effectiveness of the quality improvement intervention. The study was undertaken in four contiguous counties in central Kenya, among a population with a relatively high contraceptive prevalence rate (67%) and low total fertility rate (3.4) compared with the national averages (46% and 4.6 respectively) [22]. The proportion of women who wanted no more children (64%) was also higher than the national average (54%) [22]. The HIV prevalence rate among women in this locality was 6.2%, which was lower than the national average (8.0%) [22].

### Quality improvement intervention and its measurement

Multiple interlinked activities were implemented to improve the quality of family planning service delivery:

1. Existing national guidelines for integration of HIV and family planning services were adapted using approved service delivery protocols and a validated algorithm for delivering integrated FP/HIV services, the Balanced Counseling Strategy Plus (BCS+) [23].
2. A standardized training, mentoring and supervisory package was developed to orientate providers participating in the study.
3. Provider capacity to offer quality FP services (including certain HIV services as appropriate) was improved through a “mentorship” approach [23].
4. Facilities were provided with all of the equipment and supplies required to provide an integrated FP service based on gaps identified during a health facility assessment prior to developing and introducing the intervention.
5. Supportive supervision was conducted, bimonthly initially and subsequently on a quarterly basis, to ensure the required quality of FP services was being delivered; data management and record keeping at clinics were also strengthened.
6. Availability of information education and communication (IEC)/ behavior change communication (BCC) materials was improved by providing posters for use during group and individual counseling and making available relevant leaflets that women could take home to discuss with their partner and families.
7. The referral system between the FP service and existing antiretroviral therapy (ART) services was strengthened to enhance access to an integrated service for women living with HIV who attended the family planning service.
8. Data collection and recording systems and registers were modified to ensure that all service delivery data were accurately captured.

Early in the intervention implementation phase, it was found that the quasi-experimental design, with six intervention clinics and six matched comparison clinics, had been compromised by contamination. At that time (i.e. 2010–12), there was a rapid escalation in the investment of resources and support for activities to enhance the availability of HIV services throughout the health system as part of the Government of Kenya and its partners’ response to the HIV/AIDS pandemic. Consequently, all 12 intervention and comparison clinics were receiving various additional inputs and resources from the government and from donor-supported technical assistance organizations. As a result, the quasi-experimental design could no longer be maintained because it was not possible to attribute the quality of care received by women in the six intervention clinics to the *Integra* quality improvement and integration intervention.

Consequently, instead of the quality of care being a bivariate variable measuring ‘exposure’ or ‘non-exposure’ to the intervention, and the evaluation design comparing the quality of care received at intervention and comparison clinics, a continuous variable was developed to measure the quality of care and the evaluation design compared the quality of care score at each of the 12 clinics as measured on this continuous scale. This variable, scoring between 0 and 27, was created by aggregating the presence or absence of 27 functions of the family planning service defined through reference to the Kenyan national standards and guidelines [24]. The presence or absence of each function was measured through observations of client–provider interactions recorded by non-participant researchers following the woman’s recruitment into the study after giving her informed consent immediately before her family planning consultation began.

To generate a composite quality score (CQS) for the care received by each women during her family planning consultation, the proportion of functions observed to be performed correctly during the consultation relative to the 27 functions that comprise the definition of a high quality service was calculated for each consultation. Equal weighting was assigned to each function. Each woman’s consultation was observed for up to a further three consultations over the ensuing 24 month period using the same data collection instrument so that a CQS score could be calculated for each consultation. To facilitate understanding the programmatic implications of these measurements, the 27 functions were grouped into six domains that reflect the key elements of the service delivery experience: i) building rapport; ii) history taking; iii) use of IEC materials; iv) counselling on method use; v) HIV/STI management; and vi) documentation (S2 Table). Scores for each domain were weighted equally and calculated for each consultation and aggregated for each round to give an overall score for the quality of care received by the woman.

### Conceptualization and measurement of ambivalence

Two broad approaches have been used to measure pregnancy ambivalence, both using data collected retrospectively. One measures the difference between pregnancy intention and pregnancy affect [2, 6]; women are considered to be ambivalent if they report no explicit intention to become or to avoid pregnancy, but would be happy if they found out they were pregnant [6]. For example, Trussell and colleagues found contradictions between childbearing desires and happiness and contraceptive use. In their study, although a majority (59%) of women with contraceptive failures classified the resultant pregnancies as unintended and reported being unhappy or very unhappy, 25% said they were happy or very happy with the pregnancy [8].

The other approach is a psychometric measure that combines independent questions of how much the respondent want(ed) to get pregnant and how much they wanted to avoid getting pregnant[2]. A cross tabulation between “desire not to get pregnant” (negative) and “desire to get pregnant” (positive) generates four quadrants of pregnancy intention: Indifferent (low positive and low negative), anti-natal (low positive and high negative) pro-natal (high positive and low negative) and ambivalent (high positive and high negative) [2]. This approach creates a categorization ranging from unequivocal intentionality (positive or negative), through ambivalence, to indifference.

A variant of this psychometric measure is the London Measure of Unplanned Pregnancy [25], which comprises six questions to measure a current or recent pregnancy: fertility intentions, desire for motherhood, contraceptive use, preconception preparations, pregnancy timing, and partner influence [25]. Each item is scored and the aggregated scores are categorized as unplanned, ambivalent or planned [26].

Given the challenges with interpreting measures of intentionality, and hence ambivalence retrospectively, this analysis takes advantage of the datasets from a longitudinal study that prospectively collected information from women on their fertility desires. Our measure of ambivalence adopts an approach that incorporates the interactions between intentions and affective feelings about pregnancy and childbearing to determine whether a woman is ambivalent or unequivocal through their responses to the following questions:

1. *(Since last we talked to you) would you like to have a/child*, *or would you prefer not have any (more) children*? (Response categories were yes, no and undecided)
2. *When do you think you may have your (first or next) child*? (Response categories were within one year, between 1 and 2 years, after 2 years and don’t know)
3. *“If you found out that you were pregnant tomorrow would you be*: *happy*, *sad*, *or you would not mind*?

Women were then categorized as being ambivalent if they reported one of the following four combinations of responses:

1. Not desiring children, but will be happy if she found out that she is pregnant tomorrow;
2. Wanting a child within one year, but would be sad if she found out that she is pregnant tomorrow;
3. Wanting to wait for at least 2 years before having a (more) child(ren), but would be happy if she found that she is pregnant tomorrow;
4. Being undecided as to whether they any more (but will be sad or happy if found out that she is pregnant).

Women were categorized as unequivocal if they reported any of the following:

1. Not desiring children, and will be sad if she found out that she is pregnant tomorrow;
2. Wanting a child within one year, and would be happy if she found out that she is pregnant tomorrow;
3. Wanting to wait for at least 2 years before having a (more) child(ren), and would be sad if she found that she is pregnant tomorrow.

The conceptual framework for this analysis proposes that a woman’s propensity to feel ambiguity about becoming pregnant may be influenced by various individual-level traits. For this analysis, we were necessarily limited to including socio-demographic indicators that had been measured in the original longitudinal study. From among these we selected the following because they have been demonstrated in the literature to be associated with reported ambiguity: age, marital status, educational status, religion, economic status and parity [19]. Moreover, emerging evidence also suggests that a woman’s HIV status may also influence her pregnancy intentions [18, 19]. Consequently, we were not able to include other variables, such as partners’ intentions and desires, which have been shown in the literature to be associated with pregnancy ambivalence [27–29].

The purpose of this analysis is to test the hypothesis that the quality of care received during a woman’s family planning (FP) consultation(s) net of these personal traits influences whether she reports being ambivalent or unequivocal about a future pregnancy. Through repeated measures over time among the same cohort of women who have started using family planning, we will also assess the extent to which ambiguity varies over time.

### Sampling and data collection procedures

Representative samples of women aged 18 to 49 years receiving FP services were recruited and followed over a period of 24 months after receiving the service. Providers working in the study facilities were asked to briefly describe the study to all FP clients during the routine group health education session at the beginning of each day and inform clients that they may be approached by a member of the research team and that it would be voluntary to comply with no retribution for non-participation. Each FP client, whether new or revisit, coming for a consultation on the day(s) of the research team’s visit to the facility sequentially was invited to participate in the study until the desired sample size was reached. The eligibility criteria were: aged 18–49 years, resident in the catchment area of the health facility, and the ability to give written informed consent to participate.

The sample size for the two original cohorts had been calculated for use within the quasi-experimental study design [28] and yielded a sample size of approximately 1,000 women each for the intervention and comparison groups, including an allowance for attrition over time. The actual number of women recruited at all 12 clinics at baseline (round 0) was 1,957. The numbers of women interviewed at each subsequent round were: round 1 = 1,458 (8 months); round 2 = 1,259 (16 months); and round 3 = 1,155 (24 months). Because not all women could be interviewed at every round, the total number of women who were interviewed in all four rounds was 1,053 and these women form the sample for this analysis (S1 Fig).

Following recruitment into the study, the women’s FP consultation on that day was observed, with her informed consent, by a non-participant medically qualified observer who recorded on a standardized data collection instrument various aspects of delivery of the family planning service, including the 27 functions described above. Women were then observed at up to three subsequent FP consultations over the following 24 month period.

Upon exiting each consultation, all women were interviewed to record their fertility intentions (using the questions described above) and the socio-demographic variables listed above. For the cohort component, during the three follow up interviews, women were offered the option of the researcher meeting them at their home, at the facility (if attending for a FP service), or at a mutually agreeable location. At the time of recruitment, the woman’s name, physical address and a phone number (where available) were recorded on a sheet separate from the questionnaire and kept physically apart (in a locked cupboard). Women were informed of and reimbursed for the costs of their travel and light refreshments if interviewed away from their homes (an average of $5/Kshs350).

### Ethics

Ethical approval for the *Integra* study was granted by the Population Council Institutional Review Board and the Kenya Medical Research Institute (KEMRI) Ethical Review Board. Written informed consent was obtained from each respondent and confidentiality was assured before conducting data collection. Informed consent was obtained through providing women with detailed information about the study and requesting their agreement in writing to participate according to these principles: aims/methods of study; anticipated benefits, risks/ discomfort it may cause; the duration of the interview; voluntary participation and the fact that they have a right to refuse to answer any questions and may withdraw from the study at any time if they wish, without any reprisals.

### Analysis plan

Data collected were cleaned, edited, coded and analyzed using Stata version 12. The first step in the analysis plan was to generate descriptive frequencies and to perform cross tabulations with chi-square tests of significance. The second step involved fitting a multivariable logistic model that included all variables of interest. To identify the predictors of our measure of pregnancy intentions, a multivariable binary logistic regression with random effects model was fitted to control for unobserved characteristics of the individuals recruited within the same clinic. The third step was to model changes or “shifts” in intentionality over the 24 month period of study. To identify predictors of an intentionality shift, a multinomial logistic regression model was fitted with the same variables from the binary model. The analysis presented here was restricted to women who were observed and interviewed in all four rounds of consultations (n = 1,053).

## Results

Table 1 describes the personal socio-demographic characteristics of the women that are proposed as potentially influencing their pregnancy intentions. The women interviewed at round 3 are the sub-sample of women interviewed at round 0 who have continued to use a contraceptive method over the 24 month period; some women may still have been continuing to use at 24 months but are not included because they were lost to follow up.

**Table 1:** Background characteristics of study participants (N = 1957).

Characteristic	Round 0* (n = 1957)	Round 3** (n = 1155)
**Age in years (15–49)**		
15–24	28	13
25–29	28	28
30–34	23	26
35–39	14	20
40 plus	7	13
**Marital status**		
Never Married	5	5
Married	92	90
Formerly Married	3	5
**Education**		
Primary and below	60	59
Secondary	34	35
Tertiary	6	6
**Religion**		
Protestant	34	33
Catholic	26	26
Pentecostal	34	35
Other	6	5
**No. of living children (mean)**		
0–1	30	24
2	33	33
3	20	23
4 Plus	16	20
**HIV status**		
HIV positive	12.8	10.1
HIV Negative	83.6	88.3
Don’t know	2.3	1.2
Didn’t disclose	1.3	0.4
**Method of family planning**		
Injectables	55.5	44.0
Pills	29.5	29.2
Condoms	6.8	3.6
IUD	3.7	13.5
Implants	3.2	7.7
Other	1.3	2.0

* First interview

**Fourth (last interview)

Most women were in their late 20s or early 30s, married with two or more children, had primary level of education, and practiced a Christian faith. The most common method of family planning was injectables followed by hormonal pills. There was an increase in the proportion of long acting methods of contraception (IUD and implants) between baseline and endline.

Only two characteristics were statistically significantly different over time. The proportion of women aged 15–24 year reduced after 24 months, in part because of natural aging of the cohort and perhaps because of higher frequency of migration out of the study area as a result of school, work, or marriage, or because of higher levels of FP discontinuation. Secondly, among women who knew and reported their HIV status, the proportion reporting living with HIV had reduced after 24 months, probably due to death or higher frequency of method discontinuation among HIV-positive women.

### Pregnancy ambivalence

Table 2 describes the proportions of women’s stated intentionality concerning future pregnancy and shifts during follow up. Overall, 43% of women expressed some ambivalence about getting pregnancy at some point during the two year period; conversely, over half the women (57%) were consistently unequivocal in their stated intentions throughout the two years. A tiny minority consistently expressed ambivalence throughout all four interviews during the study period.

**Table 2:** The proportions of ambivalence and shifts during follow up (n = 1053).

Ambivalence shift	Round 0 to Round 3	
	Number	Percentage
Ambivalent*to unequivocal**	126	12
Unequivocal to Ambivalent	321	31
Remained Ambivalent	4	0.4
Remained unequivocal	602	57
**Total**	**1053**	**100**

*Inconsistent or contradictory feelings in pregnancy intentions

** Clear cut and unambiguous feeling in pregnancy intentions

### Quality of care experienced

Table 3 presents overall mean scores for the indicators of quality of care received by all women across all four rounds of data collection in the two year period. Overall, there was a slight improvement in the quality of care experienced during the two year period (from 10.5 to 11.5), although this change was not statistically significant. There were variations among the domains of care, with building rapport and documentation registering significant improvements, while history taking experienced a significant decline. The decline in history taking may reflect the fact that these were repeat consultations with clients and so their records should already contain sufficient information about the client that the provider does not need to collect again.

**Table 3:** Quality of care scores (mean scores) by round of data collection.

Domain	Round 0	Round 1	Round 2	Round 3	Total	P value
Building rapport (0–5)	2.7	3.2	3.6	3.9	3.3	<0.001
History taking (0–4)	1.9	1.8	0.7	0.9	1.4	<0.001
Use of IEC* materials (0–3)	0.9	0.7	0.9	1.1	0.9	0.056
Counselling on method use (0–3)	1.5	1.2	1.4	1.6	1.5	0.008
STI management (0–10)	2.3	1.9	2.6	1.8	2.2	0.04
Documentation (0–2)	0.7	1.8	1.9	1.9	1.4	<0.001
Overall process scores (0–27)	10.5	10.7	11.4	11.5	11.0	0.003

* Information, education communication

### Associations between pregnancy ambivalence, quality of care and explanatory variables

Table 4 presents the results (log odds) from multivariate analyses of the associations between the explanatory variables and the likelihood of a woman expressing ambivalence or being unequivocal at least once during the four interviews. Four variables were significantly associated with both ambivalence and being unequivocal: age (except ambivalence among older women); number of living children; shift in fertility desires; and HIV status. Women expressing ambivalence were more likely to be older than 25 years, have one child or none, changed their desired fertility during the time period, and be HIV negative. Conversely, women who were unequivocal throughout the time period were those aged 15–24 years, had two or more children, did not change their fertility desires and were living with HIV; additionally, women who want no more children were twice as likely to be unequivocal than women wanting more.

**Table 4:** Multivariate model predicting ambivalence (n = 1053).

Characteristic	Ambivalent		Unequivocal	
	Odds ratio	P-value	Odds ratio	P-value
**Quality of care**	0.95 (0.92–0.96)	0.005	1.03 (1.02–1.05)	0.030
**Age**				
15–24 (ref)	1.00		1.00	
25–29	1.48 (1.32–1.93)	0.004	0.54 (0.43–0.67)	<0.001
30–34	1.71 (1.25–2.33)	0.001	0.44 (0.34–0.56)	<0.001
35–39	1.81 (1.24–2.63)	0.002	0.44 (0.28–0.58)	<0.001
40 plus	1.82 (1.12–2.97)	0.016	0.40 (0.28–0.58)	<0.001
**Marital status**				
Married (ref)	1.00		1.00	
Never Married	0.93 (0.58–1.50)	0.764	1.16 (0.81–1.67)	0.419
Formerly Married	0.53 (0.28–1.01)	0.052	1.33 (0.89–1.99)	0.160
**Education Status**				
Primary & less (ref)	1.00		1.00	
Secondary	0.90 (0.73–1.12)	0.338	1.07 (0.91–1.26)	0.434
Tertiary	0.91 (0.60–1.38)	0.652	1.11 (0.79–1.56)	0.556
**Household wealth**				
Poorest (ref)	1.00		1.00	
Lower Middle	0.95 (0.69–1.30)	0.736	1.13 (0.89–1.44)	0.286
Middle	1.23 (0.91–1.67)	0.181	0.88 (0.69–1.11)	0.286
Upper Middle	1.06 (0.78–1.45)	0.692	0.89 (0.70–1.13)	0.333
Richest	0.95 (0.68–1.33)	0.755	1.04 (0.80–1.34)	0.765
**Religion**				
Catholic (ref)	1.00		1.00	
Protestant	1.04 (0.81–1.32)	0.742	1.04 (0.86–1.26)	0.700
Pentecostal	0.97 (0.76–1.24)	0.794	1.26 (1.05–1.53)	0.014
Other	0.71 (0.41–1.22)	0.212	1.64 (1.11–2.43)	0.013
**Living children**				
0–1 Child (ref)	1.00		1.00	
2 Children	0.62 (0.48–0.80)	<0.001	1.99 (1.61–2.47)	<0.001
3 Children	0.29 (0.21–0.42)	<0.001	4.13 (3.14–5.43)	<0.001
4 and more	0.19 (0.12–0.31)	<0.001	7.35 (5.24–10.32)	<0.001
**Fertility desire at Round 0**				
Wants more	1.00		1.00	
No more	0.73 (0.58–0.93)	0.010	1.99 (1.61–2.47)	<0.001
**Fertility desire shift**				
Consistent fertility desire	1.00		1.00	
Changed fertility desire	1.42 (1.17–1.72)	<0.001	0.53 (0.45–0.62)	<0.001
**HIV status**				
Negative/Unknown (ref)	1.00		1.00	
HIV Positive	0.51 (0.35–0.75)	0.001	2.43 (1.85–3.19)	<0.001

As women get older they have increased odds of expressing ambivalence, but as parity increases women have increased odds of being unequivocal in their pregnancy intentions; indeed, women with three children had four times increased odds of being unequivocal and those with four or more children had seven times increased odds of being unequivocal.

Women receiving a higher quality of care had reduced odds of expressing ambivalence about their pregnancy intentions at any point in time, controlling for other factors in the model. However, although statistically significant, the odds ratio of 0.95 indicates that this association is not particularly strong.

### Associations with a shift in pregnancy ambivalence

Data were analyzed to determine any associations with a shift in intentionality over time. Table 5 presents the results from a multinomial logistic regression model. The three outcomes in the model are shift from ambivalence to unequivocal, shift from unequivocal to ambivalent and no shift over time. The estimates are adjusted for clustering at a health facility.

**Table 5:** Multivariate model predicting ambivalence shift(n = 1053).

Characteristic	Ambivalent to unequivocal Vs No shift		Unequivocal to ambivalent Vs no shift	
	Odds ratio	P-value	Odds ratio	P-value
**Quality of care**	0.94 (0.86–1.02)	0.152	0.99 (0.95–1.04)	0.758
**Age**				
15–24 (ref)	1.00		1.00	
25–29	1.84 (1.09–3.10)	0.023	1.86 (1.38–2.50)	<0.001
30–34	2.89 (1.54–5.40)	0.001	2.04 (1.11–3.75)	0.022
35–39	3.11 (1.14–8.47)	0.026	1.88 (0.90–3.93)	0.093
40 plus	2.54 (0.92–7.04)	0.073	2.43 (0.83–7.19)	0.107
**Marital status**				
Married (ref)	1.00		1.00	
Never Married	1.44 (0.69–2.96)	0.329	1.08 (0.56–2.10)	0.819
Formerly Married	1.43 (0.82–2.50)	0.211	0.72 (0.35–1.50)	0.384
**Education Status**				
Primary & less (ref)	1.00		1.00	
Secondary	0.90 (0.57–1.42)	0.638	0.92 (0.59–1.42)	0.702
Tertiary	1.03 (0.46–2.30)	0.942	0.76 (0.39–1.48)	0.416
**Household wealth**				
Poorest (ref)	1.00		1.00	
Lower Middle	0.79 (0.50–1.22)	0.286	0.90 (0.55–1.47)	0.664
Middle	0.65 (0.31–1.33)	0.234	1.30 (0.81–2.09)	0.266
Upper Middle	0.87 (0.45–1.66)	0.669	1.27 (0.84–1.90)	0.256
Richest	0.99 (0.66–1.50)	0.972	0.95 (0.51–1.78)	0.874
**Religion**				
Catholic (ref)	1.00		1.00	
Protestant	0.79 (0.42–1.50)	0.475	1.12 (0.72–1.75)	0.606
Pentecostal	0.70 (0.38–1.31)	0.268	0.84 (0.65–1.09)	0.189
Other	0.84 (0.28–2.49)	0.755	0.55 (0.24–1.27)	0.159
**Living children**				
0–1 Child (ref)	1.00		1.00	
2 Children	0.48 (0.38–0.61)	<0.001	0.49 (0.31–0.78)	0.003
3 Children	0.26 (0.15–0.44)	<0.001	0.23 (0.11–0.46)	<0.001
4 and more	0.16 (0.09–0.29)	<0.001	0.13 (0.60–0.28)	<0.001
**Fertility desire at Round 0**				
Wants more	1.00		1.00	
No more	0.57 (0.28–1.17)	0.128	0.55 (0.35–0.86)	0.008
**Fertility desire shift**				
Consistent fertility desire	1.00		1.00	
Changed fertility desire	2.43 (1.51–3.93)	<0.001	1.75 (1.44–2.13)	<0.001
**HIV status**				
Negative/Unknown (ref)	1.00		1.00	
HIV Positive	0.31 (0.17–0.58)	<0.001	0.47 (0.21–1.05)	0.064

Three factors had statistically significant associations with a shift in ambivalence: age, parity and a change in fertility desire. Women aged 20 and over and women who changed their fertility desire had increased odds of becoming unequivocal or ambivalent over time. Women with two or more children had reduced odds of becoming ambivalent or unequivocal over time compared to women with none or one child. Likewise women who were living with HIV had reduced odds of moving from being ambivalent to being unequivocal compared to those who were HIV negative. Quality of care was not associated with the likelihood of women shifting to or from ambivalence.

## Discussion and conclusion

Ambivalence in fertility preferences is critical because it has been associated with nonuse or inconsistent use of contraception, thereby exposing women to unintended pregnancy [6]. Understanding ambivalence in fertility preferences and being able to recognize and incorporate it during counseling on and provision of contraception is important if women are to be provided with information and services to reduce unintended pregnancies [6]. The findings presented here represent the first analyses to explore both the measurement of ambivalence itself and its association with programmatic and other explanatory factors.

Findings reported from this study should be interpreted in the light of several limitations. The first limitation is the loss to follow up between rounds, which reduced the sample size by more than a third over the course of two years. However, apart from HIV status and age (there was a higher rate of follow up among the HIV negative and those aged over 24), the baseline and endline background characteristics were similar and so we expect this not to have a significant effect on our analysis. Secondly, it is possible that using face-to-face interviews to gather information on personal matters such as pregnancy intentions and affect may have contributed to reporting bias as people might have a problem revealing them to a stranger. Thirdly, the FP quality improvement intervention was integrated within a broader strategy to integrate HIV services within existing FP services, and so the quality improvement activities necessarily included elements that went beyond simply addressing the quality of the FP service itself. Finally, we did not collect detailed reproductive history of our cohort and thus missed out on potential cofounders such as miscarriages and abortions.

Our analysis found that over half (57%) of the women in this study were unequivocal about their fertility desires throughout the two year period of observation, but only 0.4 percent were ambivalent throughout the period. Thus two out of five women shifted between being ambivalent or unequivocal at some point during the two years; such women were likely to be older or to have none or one child, to have expressed a change in their desired family size and to be HIV negative.

Paradoxically, younger women aged 15–24 years are more likely to remain unequivocal and the least likely to shift from one position to another, yet those with none or one child, who are often in this age group, are more likely to have been ambivalent at some point in time and to have had an ambivalence shift.

Women receiving services at facilities with higher levels of quality of care are statistically less likely to report ambivalence, net of other factors, although this is not a strong association and there is no statistical association with being more likely to be unequivocal. Thus while we can conclude that higher quality counseling appears to help women hold less ambivalent pregnancy intentions, the lack of association with ambivalence shift also suggests that this is a weak association.

For providers to effectively provide family planning counseling and services, pregnancy intentions must be accurately assessed. While further research is needed to understand which counseling techniques effectively help women expressing ambivalence, acknowledging that ambivalence towards pregnancy exits is important for the development of policy and service delivery interventions. Women’s commitment and motivation to avoiding pregnancy is known to affect their contraceptive behavior [30]; women who are ambivalent about becoming pregnant or avoiding a pregnancy are less likely to use contraception[30] and more likely to have gaps in contraceptive use, thereby exposing them to the risk of unintended pregnancy [11]. Better understanding of dimensions of pregnancy intentions may improve ways of helping women to prevent unplanned pregnancies. Health care providers should discuss pregnancy risks and contraceptive options with women who are not motivated to prevent pregnancy before it occurs.

The inclusion of discussions during counseling on a woman’s desire to become or avoid pregnancy and the affective dimension of her becoming pregnant is highly advisable. The DHS measures the wanted status of pregnancy in the last five years, and has a question on whether becoming pregnant soon would be a problem for the woman. However, this question might not capture ambivalence as commonly understood and personally defined. The term “problem” is broad and might be interpreted to include problems related to physical ability to carry a pregnancy as well as financial ability to rear a child, as well as feelings of well-being or happiness. There is need for additional measures on happiness, and other measures that better assess the strength of desire to avoid/become pregnant in the large-scale surveys such as the DHS.

## Supporting information

S1 Text Original, unpublished version of the study protocol approved by the ethics committee. (DOCX)

S1 Fig Participant flow diagram. (DOCX)

S1 Table Trend statement checklist. (DOCX)

S2 Table Annex 1: Attributes of service delivery. (DOCX)
