# Supplementary material for: Ambivalence in pregnancy intentions: The effect of quality of care and context among a cohort of women attending family planning clinics in Kenya
Source: PLoS One. 2018 Jan 9;13(1):e0190473. doi: 10.1371/journal.pone.0190473 (PMC5760043; doi:10.1371/journal.pone.0190473)
Supplement: S1 Text — (DOCX) [file pone.0190473.s001.docx]

**Original, unpublished version of the study protocol that the ethics committee approved before the trial began.**

**Project Title**: **Assessing the benefits and costs of integrating HIV into FP services in Kenya**

**Principal Investigators:**

Charlotte Warren

[cwarren@popcouncil.org](mailto:cwarren@popcouncil.org)

Population Council

General Accident Insurance House

Ralph Bunche Road

PO Box 17643

Nairobi 00500

Kenya

Tel: +254 20 2713480

Charlotte Watts

Charlotte.Watts@lshtm.ac.uk

London School of Hygiene and Tropical Medicine

Room 403,

Gordon Square,

London WC1H OPD

Susannah Mayhew/ Andrew Sloggett (acting PI) asloggett@lshtm.ac.uk

Susannah.Mayhew@lshtm.ac.uk

Centre for Population Studies

49-40 Bedford Square

London School of Hygiene and Tropical Medicine

London WC1B 3DP

**Location**: Central Province, Kenya

**Project dates:** January 2009 – October, 2012

**Study Coordinator**: Ian Askew

**Signatures of PIs**:


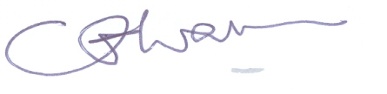
 21^st^ October 2008

Charlotte Warren

Submitted to the Population Council IRB on 22^nd^ October 2008

Submitted to KEMRI/NERC 24^th^ October 2008 (NON SSC NO. 113)

Amended version submitted December 18^th^ 2008 IRB 443

# Summary of Proposed Research

A consortium of three organizations – Population Council, London School of Hygiene and Tropical Medicine (LSHTM) and the International Planned Parenthood Federation (IPPF) – funded by the Bill and Melinda Gates Foundation (BMGF), have come together to address the lack of evidence around the feasibility, effectiveness, cost and impact of a range of existing or potential models for delivering integrated HIV and SRH services in high and medium HIV prevalence settings. This project will seeks to answer three key sets of research questions:

1. What are the relative benefits of different models of integrated SRH and HIV services over separately provided services? Specifically, does integration lead to: a) increases in numbers of clients using services; b) Changes in the profile of clients attending services; c) Increases in the range of services accessed by clients; and d) improvements in the quality of services?
2. In the target populations, what is the impact of integrated services on: a) HIV related risk behavior; b) HIV related stigma; and c) Unintended pregnancy?
3. What is the cost, feasibility and cost-effectiveness of providing selected integrated services: a) What is the cost of integrating HIV and/or SRH services with existing services; b) How do costs vary by model of integration; and c) Does integration result in a more optimal utilization of existing infrastructure and human resources?

This study will be carried out in 12 Ministry of Health facilities in Central Province Kenya and 6 IPPF affiliate facilities and aims: to determine the feasibility, acceptability, cost and immediate effectiveness of providing an expanded package of integrated HIV and RH services to new and revisiting FP clients; and to determine the impact of this integration model on client behaviors and their health status. The study comprises of three designs: 1) Quasi- experimental time series assessment within facilities 2) Static post test comparison following experimental and control cohorts of clients and 3) post test only surveys of community perceptions among experimental and control communities.

Facility based research will be conducted at various stages during the study to track changes over time for the range of integrated services offered. Facility inventories, costing tools, structured observations, and client exit interviews will be the methods used in order to collect comparable data across each clinic. Although health providers will be observed providing care and interviewed the main target population is women aged18 years and above who are accessing family planning services. In addition to the periodic exit interviews, a cohort of women will be approached and requested to interview them at intervals over a two and half year period, with in the facility or at home depending on their preference. A community survey will also be carried out in two locations where both women and men who may or may not use services will be approached for interview.

This project deals with some issues that are considered sensitive in nature (SRH behaviour). Ethical considerations in this project relate to risk and risk management, protecting confidentiality and anonymity of the informants, compensation, and obtaining consent and assent. In addition to submitting this proposal to the Population Council’s Institutional Review Board, the proposal will be reviewed by Division of Reproductive Health, Kenya Medical Research Institute (KEMRI) IRB for local ethical review and research authorization.

# Abbreviations and Acronyms

AIDS Acquired Immuno Deficiency Syndrome

ARV Anti retro viral

ART Antiretroviral therapy

BCC Behaviour change and communication

BMGF Bill and Malinda Gates Foundation

BNIM Biographic Narrative Interpretive Method

CCC Comprehensive Care Centre

CD4 Cluster of Differentiation Antigen 4 (glyco- protein found on surface of helper key cells)

CPI Client – Provider Interaction

CT Counseling and Testing

DHMT District Health Management Team

DPT Diphtheria Pertussus and Tetanus

DRH Division of Reproductive Health

EA Enumeration Area

FGD Focus Group Discussion

FHOK Family Health Options of Kenya

FP Family Planning

HAART HIV/AIDS Antiretroviral Therapy

HFA Health Facility Assessment

HIV Human Immuno-deficiency Virus

IDI In depth interview

ID Identity

IEC Information, Education and Communication

IPPF International Planned Parenthood Federation

IUD Intra Uterine (Contraceptive) Device

KAIS Kenya AIDS Indicator Survey

KDHS Kenya Demographic Health Survey

KEMRI Kenya Medical Research Institute

LAM Lactational Amenorrhea Method

LSHTM London School Hygiene and Tropical Medicine

LQA Lots Quality Assurance

MCH Maternal and Child Health

MNH Maternal and Newborn Health

MOPH S Ministry of Public Health and Sanitation (Kenya)

MOMS Ministry of Medical Services (Kenya)

NASCOP National AIDS and STDs Control Program

PHT Public Health Technician

PLWH People living with HIV

PMTCT Prevention of Mother to Child Transmission

SGPT /ALT Serum glutamic-pyruvic transminase /Alanine -amino -tranasferase

SRH Sexual and Reproductive Health

STI Sexually Transmitted Infection

RH Reproductive Health

UNAIDS United Nations HIV Program

UNFPA United Nations Population Fund

USAID United States Agency for International Development

VCT Voluntary Counseling and Testing

WHO World Health Organisation

Contents

[Summary of Proposed Research ii](#_Toc217365560)

[Abbreviations and Acronyms iii](#_Toc217365561)

[Background 1](#_Toc217365562)

[Specific Aims 3](#_Toc217365563)

[Description of the Subject Populations 4](#_Toc217365564)

[Research Protocol 13](#_Toc217365565)

[Informed Consent. 23](#_Toc217365566)

[Appendix 1: Consent for provider interview 25](#_Toc217365567)

[Appendix 2a: Consent for client to observe provider-client interaction 27](#_Toc217365568)

[Appendix 2b: Consent from Client for short interview following CPI 29](#_Toc217365569)

[Appendix 3A: Consent from Client for cohort study 31](#_Toc217365570)

[Appendix 3B: Client tracing form for Cohort study 33](#_Toc217365571)

[Appendix 3C Informed consent during home visit 34](#_Toc217365572)

[Appendix 4A: Community Survey: Household Roster Form 36](#_Toc217365573)

[Appendix 4B: Introductions for use in community surveys 37](#_Toc217365574)

[Appendix 4C: Parent/guardian Information sheet - community survey 39](#_Toc217365575)

[Appendix 4D: Parent/guardian consent form 41](#_Toc217365576)

[Appendix 5A: Participant Information Sheet (FGD) Community Based 42](#_Toc217365577)

[Appendix 5B: Participant consent form 44](#_Toc217365578)

[Appendix 5C: Participant Information Sheet (Provider) 45](#_Toc217365579)

[Appendix 5D: Provider consent Form 47](#_Toc217365580)

[Appendix 6: Key Research Personnel 48](#_Toc217365581)

[Appendix 7: Research Ethics Certificates for Population Council staff 50](#_Toc217365583)

[Appendix 8: Final drafts of study instruments: 51](#_Toc217365584)

[Appendix 8.1: CPI 52](#_Toc217365585)

[Appendix 8.2: Client exit 61](#_Toc217365586)

[Appendix 8.3: facility inventory 71](#_Toc217365587)

[Appendix 8.4: Provider Knowledge 80](#_Toc217365588)

[Appendix 8.5: client cohort 85](#_Toc217365599)

[Appendix 8.6: Community Survey of Women 93](#_Toc217365600)

[Appendix 8.7: Community Survey of Men 101](#_Toc217365601)

[Appendix 8.8: Client in-depth interview guide 109](#_Toc217365602)

[Appendix 8.9: FP/RH Client in-depth interview guide 115](#_Toc217365603)

[Appendix 8.10 FP/RH Provider in-depth interview guide 121](#_Toc217365604)

[Appendix 8.11: HIV Provider in-depth interview guide 128](#_Toc217365605)

[Appendix 8.12: Economics component periodic activity review tool 133](#_Toc217365606)

# Background

There are many well-established reasons that support the rationale for integrating or linking sexual and reproductive health (SRH) and HIV services in developing countries. Most HIV infections in these countries are sexually transmitted or associated with pregnancy, childbirth, and breastfeeding. Many of the behaviors that prevent HIV transmission also prevent sexually transmitted infections (STIs) and some prevent unintended pregnancies. Individuals who use SRH services often benefit from HIV/AIDS services, and vice versa. Further, with nearly 40 million people living with HIV today, there is a clear need to respond to the sexual and reproductive health needs of HIV positive people.

A number of international statements, position papers and advocacy efforts indicate the commitment of the international community to intensifying linkages between SRH and HIV and AIDS, at both a policy and programmatic level^[[1]](#footnote-1)^. At a regional level the recent policy meeting in Maputo affirmed the need to act on the bi-directional linkages^[[2]](#footnote-2)^. An interagency document produced by WHO, UNFPA, UNAIDS and IPPF, entitled *“Sexual and Reproductive Health and HIV/AIDS, A Framework for Priority Linkages*”^[[3]](#footnote-3)^, highlights some of the prioritized strategic programmatic linkages between services for SRH and HIV and outlines the ‘expected’ benefits, which include: a) improved access to and use of key HIV/AIDS and SRH services; b) efficiencies in providing similar services jointly rather than separately; and c) reduced HIV/AIDS-related stigma and discrimination.

Furthermore, the *Framework* recognizes the imperative for improving the evidence-base needed to ascertain the magnitude of these benefits. This need for more evidence to develop and inform policies and strategies for effective integration was further underlined at an international conference organized by the Bill and Melinda Gates Foundation (BMGF) in Ethiopia, entitled “Linking Sexual and Reproductive Health, Family Planning and HIV”^[[4]](#footnote-4)^. Moreover, a series of meetings to be held in October 2008, convened by USAID and WHO, will seek to determine the state of the art in programming for a variety of linkages. A forthcoming Cochrane Review of evaluations of integrated SRH and HIV/AIDS services highlights the absolute dearth of empirical evidence for virtually all models of integrated services.

The Population Council has undertaken a number of evaluative studies that contribute to this limited evidence base, which form the basis on which this protocol has been developed. In particular, two studies have been completed, one in South Africa and one in Kenya, that have developed, implemented and evaluated alternative models for integrating HIV services into family planning (FP) services. In brief, these studies used quasi-experimental designs to evaluate models for integrating STI/HIV counseling and risk assessment, together with providing HIV testing, into services for FP clients. Both studies had similar results; for example, the study in Kenya^[[5]](#footnote-5)^ found that:

- Integrating STI/HIV counseling and offering HIV counseling and testing (CT) within FP services was feasible and acceptable to clients and providers.
- Overall quality of care improved significantly, most probably due to use of the Balanced Counseling Strategy Plus tool. Average quality scores were significantly higher during consultations where providers used the BCS+ tool (18.86) than where they did not (12.19).
- Counseling on HIV CT increased significantly during FP consultations. The proportion of providers discussing CT increased significantly between baseline (39%) and endline (88%)
- A significantly greater proportion of providers offered an HIV test following the intervention (from 1% at baseline to 39% for repeat clients and 48% for new clients).
- Between half and three-fourths of the women who were offered an HIV test accepted—35 percent among women attending a clinic where testing was offered as part of the consultation, and 20 percent among women who were referred elsewhere for the test.
- Integrating STI/HIV counseling and HIV CT into FP services increased the average time spent with clients from about 10 minutes to 14 or 15 minutes. Moreover, the cost of staff for this additional time (between $0.24 and $0.41 per client) was reasonable. Estimated costs for onsite CT range from $5.60 per client at a hospital setting to $9.53 in a dispensary, which compare favorably with an estimated per-client cost of $27 at a stand-alone CT clinic^[[6]](#footnote-6)^).

Clients attending for FP services are sexually active, consistent condom use is low, and in a mid to high prevalence epidemic they may well be at risk of HIV infection, or already be infected but not know their status. As FP services gradually become more widely used in Africa, they provide an important opportunity to increase access to both HIV testing services and to Anti-retroviral (ARV) services and other care and support services for those FP clients found to be positive.

A consortium of three organizations – Population Council, London School of Hygiene and Tropical Medicine (LSHTM) and the International Planned Parenthood Federation (IPPF) – funded by the BMGF, have come together to address this lack of evidence around the feasibility, effectiveness, cost and impact of a range of existing or potential models for delivering integrated HIV and SRH services in high and medium HIV prevalence settings. This project will answer three key sets of research questions:

1. What are the relative benefits of different models of integrated SRH and HIV services over separately provided services? More specifically, does integration lead to:
   1. Increases in the numbers of clients using services;
   2. Changes in the profile of clients attending services;
   3. Increases in the range of services accessed by clients;
   4. Improvements in the quality of services?
2. In the target populations, what is the impact of integrated services on:
   1. HIV related risk behavior;
   2. Stigma associated with HIV care;
   3. Unintended pregnancy?
3. What is the cost, feasibility and cost-effectiveness of providing selected integrated services:
   1. What is the cost of integrating HIV and/or SRH services with existing services;
   2. How do costs vary by model of integration;
   3. Does integration result in a more optimal utilization of existing infrastructure and human resources?

# Specific Aims

This protocol describes a study to evaluate the feasibility, effectiveness, impact and cost of integrating HIV services into FP services in Kenya^[[7]](#footnote-7)^. The study, which builds on the experiences gained in the study described above, has two specific aims:

1. The previous study (Liambila et al 2008) demonstrated a need for additional services following the standard package of HIV/STI prevention and VCT awareness information, individual risk assessment and dual protection during all FP consultations. Additional services are required for those FP clients who already know they are HIV positive or are found to be positive after testing as a result of the integrated service.

The main service identified, was to strengthen the linkage with ARVs and other care and support services for women (and their partners) known or found to be HIV positive. These HIV services are currently unsystematic and vary considerably according to clinic and provider. This project will systematically increase access to ARV and other HIV care and support services for those FP clients found to be HIV positive at two levels: at district hospitals that already function as ‘Comprehensive Care Centres’ (CCCs), where ARV services (pre-HAART and HAART) are already available on-site and so FP clients will be linked directly to these services; at health centers and dispensaries, where systematic referral linkages to ARV services available in other facilities (including CCCs) will be established for those FP clients known or found to be HIV positive. Moreover screening and management of STIs for all clients during the FP consultation will be strengthened.

The first aim of this research, therefore, is to determine the feasibility, acceptability, cost and immediate effectiveness of providing this expanded package of integrated services to new and revisiting FP clients.

The expected results include:

- Feasibility of linking these services demonstrated
- Provision of linked services reported as acceptable by providers and clients
- Increased numbers/proportions of eligible HIV+ women starting to use ART
- Increased numbers/proportions of FP clients receiving integrated CT and STI counselling and screening services
- Increased numbers/ proportions of clients screened for STIs
- Increased numbers/ proportions of clients treated for STIs?
- Increased numbers/ proportions of new and repeat FP clients first testing (and retesting) for HIV
- Improved attitudes of service providers towards HIV+ clients
- Total resource requirements and unit costs of intervention known
  - Cost per eligible client receiving ART,
  - Cost per person-month of receiving ART
  - Cost per client counseled, tested and receiving results
  - Cost per client receiving each service component
  - Cost per client receiving each service component
  - Cost per client counselled, tested and receiving results

1. Previous research also demonstrated the acceptability, feasibility, costs and immediate effects of integrating HIV/STI counseling and risk assessment, together with HIV CT, into FP services. The second aim of this research is to determine the impact of this integration model on client behaviors and their health status; that is, whether FP clients who receive this integrated package of services are more likely to adopt risk reduction behaviors for infection prevention and avoidance of unintended pregnancy, and to achieve their desired health status, in terms of acquiring or transmitting STI/HIV and a planned reproductive intention, than FP clients that receive a standard FP service. The costs of achieving these health status indicators will be estimated. The research will also determine whether providing integrated services affects community perceptions of: a) stigma around HIV services; and b) the quality of care provided when FP services are provided individually or in combination with HIV services.

The expected results include:

- Reduction in reported HIV risk behaviors among HIV negative and HIV positive clients (measures include: condom use at last sex; number of partners in past 12m)
- Reduced incidence of unintended pregnancies
- Increased duration of contraceptive use among HIV negative and HIV positive clients
- Improved attitudes of service providers towards HIV positive clients
- Increased compliance with ART among those on treatment
- Reduction in clients reporting unacceptable or stigmatizing behavior by providers
- Decreased stigmatization at community level of HIV services when integrated with FP and other RH services

# Description of the Subject Populations

Population Council and LSHTM will address these research aims in a sample of 18 facilities, including 12 MOH facilities and 6 FHOK facilities, so as to control for and compare the anticipated differences between service delivery systems and quality of service provided in the public and non-governmental sectors. Starting with the existing 23 facilities from the previous study (Liambila et al 2008) and building on that experience, six intervention facilities were selected. The two key criteria for selecting the six facilities were those that performed well in the study and had a high throughput of FP clients (100 or more per month). These six intervention facilities include two hospitals and four health centres.

In order to match these sites with six comparable or control sites and avoid contamination, additional criteria were used to select facilities with similar characteristics from outside the two original study districts. These criteria include high client load (more than 100 FP per month), a minimum of two FP providers qualified in and currently providing FP services, a range of services (FP. VCT, STI treatment, PMTCT): no provision of integrated CT-FP services. All hospitals are in peri-urban locations and health centres in rural locations.

We have used randomized pair-wise matching to select the six comparison clinics according to the criteria described above. These facilities serve populations with similar socio-economic characteristics, have a similar health infrastructure (i.e. public sector), and serve a population with a relatively high modern method contraceptive prevalence rate (58%) compared with the national average (33%) (KDHS 2003) and similar HIV prevalence among women aged 15 to 49 years in Central Province 7.6% compared to the national level of 8.7% (KAIS 2008) .

*Map of Central Province outlining districts*


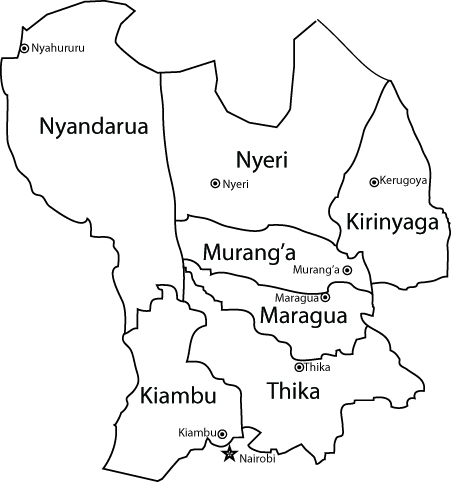


To address the research aims within the context of the FHOK program^^[[8]](#footnote-8)^^, three FHOK clinics currently providing integrated HIV/RH services (Nairobi, Thika and Nakuru) will be compared with three FHOK clinics (Meru, Eldoret and Kisumu) that provide RH services (with some HIV care) but do not offer an integrated model. Facility based research will be conducted at three stages during the study in order to track changes over time in clinic preparedness for the range of services offered, quality of care indicators, and client satisfaction. Facility checklists, structured observations, and client exit interviews will be the methods used in order to collective comparable data across each clinic. This information will be analysed together with the routine program data from each site (including numbers of clients, and socio-demographic profiles). The clinics offering an integrated model of care will be compared with those offering vertically organised services to prospectively assess the strengths and weaknesses of each model.

Population Council will be responsible for research in the MOH facilities and LSHTM for research in the FHOK clinics. LSHTM will also be responsible for the economics study in the MOH and FHOK facilities and within the community. The same sampling, data collection procedures and tools will be used in both MOH and FHOK clinics.

Table 1 lists the intervention and comparison sites.

**Table 1: Selected Facilities**

| **CT/FP in place 2008** | **No formal integration at June 2008** |
| --- | --- |
| Nyeri PGH, Nyeri | Murang’a North District Hospital |
| Ngorano HC, Nyeri | Kigumo HC, Maragua district |
| Warazo HC, Nyeri | Kangari HC, Maragua district |
| Thika District Hospital,Thika District | Nyahururu District H, Nyandarua District |
| Ruiru HC Thika District | Engineer HC, Nyandarua District |
| Kiruara HC Thika District | Njabini  HC, Nyandarua District |
| IPPF/FHOK Thika | IPPF/FHOK Kisumu |
| IPPF/FHOK Nairobi | IPPF/FHOK Meru |
| IPPF/FHOK Nakuru | IPPF/FHOK Eldoret |

There are four subject populations in this study:

1. Health care providers delivering FP services

All health care providers that provide FP services in the study clinics at the time of data collection will be requested to participate in the study. Providers will be interviewed to assess their knowledge concerning FP, STI and HIV/AIDS services and to understand their attitudes around integrated SRH/HIV services. For the hospitals and FHOK facilities, it is expected that between four to eight providers will be requested to participate in the study, and at health centers the number is expected to be between two and four. This will give a sample size of between 16 – 32 providers for each group of MOH facilities and between 12 – 24 providers for each group of FHOK facilities.

The quality of service they provide will be assessed through non-participant observations of their interactions with FP clients. Providers will be approached on four occasions at baseline, 5 months after the intervention, around 18 months and 30 months later.

Recruitment of providers will be as follows. Following sensitization meetings held with the district health management teams, Population Council researchers will hold group meetings with the management and health care providers of each facility to introduce the purpose and methods of the study and to request their participation in interviews. Interviews with each provider will be scheduled at a time that does not take them away from attending to their clients’ needs, either at their break times or during the afternoon when facilities are less busy. At the time of the interview, the benefits and risks of their participation will be described to each provider and they will be assured that consenting to be interviewed is voluntary and all information provided will be kept in confidence (see informed consent form in appendix 1). No-one, including their supervisor, will know what they say and their names will not appear on the questionnaire sheet. However the overall information and opinions from all providers will be revealed but it will not be known who said what.

1. Women of reproductive age accessing FP services

A representative sample of women aged 18 years and above seeking FP services will be recruited in the selected sites to assess the quality of FP and other services provided. Providers working in the study facilities will be asked to briefly describe the study to all FP clients during the routine health education session at the beginning of each day so that they understand the study objectives. The provider will inform clients that they may be approached by a member of the research team, but assure clients that it is voluntary to comply.

For the purposes of this study, it is assumed that the quality of service observed across a representative number of FP consultations at a particular facility will be a valid and reliable measure of the overall quality of service available at that facility. In each study facility, a qualified nurse-midwife will be trained to be a non-participant observer of 18 new and 18 revisit FP consultations in each facility, that is, 108 new and 108 revisit clients for each study group. Each client observed will also be interviewed immediately after the consultation to measure their perceptions of the service received. The mean quality of service for each study group will then be calculated by aggregating quality of service scores for all 108 new and 108 revisit clients at all facilities in that group.

These cross-sectional samples of clients will be recruited, observed and interviewed at three points for the control facilities and four points in the intervention facilities groups to allow the quality of care provided to be assessed before and after introduction of the interventions and to be compared between those exposed and not exposed to the interventions. Samples of women will be recruited at the following time:

- Immediately before the interventions are introduced - all sites
- Five to six months after the intervention (intervention sites only)
- 15- 18 months after intervention - all sites
- 27 - 30 months after intervention all sites

At the start of the consultation, each new and revisit FP client aged18 years and above attending for a consultation on the day of the research team’s visit to the facility will be invited to participate in the study until the desired sample size is reached. Their participation will be through allowing a non-participant researcher to observe their FP consultation with the provider and also agreeing to be interviewed separately and in privacy after the consultation. If the client agrees to be both observed and interviewed she will be assured that her participation in the study is completely voluntary and nothing recorded during the observation or interview will be communicated to anyone else (see informed consent forms in appendices 2A and 2B). Following the client’s consent, a researcher will sit in the corner of the room and observe the client’s interaction with the provider. Observations of the interaction will be recorded on a structured checklist to assess the quality of care provided according to a set of items that will measure the essential elements of an integrated FP/STI/HIV consultation.

On completion of the consultation, the client will be given a piece of paper with a number and asked to give it to the interviewer seated outside the consultation area for the short interview. This number will be recorded on both the observation checklist and the exit questionnaire and linked only for analysis. Consent for the interview will be obtained separately through use of the informed consent procedure outlined in appendix 2B) Women will be asked about their perceptions of and satisfaction with the consultation and about their SRH and HIV-related behaviors and fertility intentions.

LSHTM will also carry out in-depth interviews (IDIs) and focus group discussions (FGDs) with health care providers and key informants. These will take place alongside the community surveys, as well as on an *ad hoc* basis when needed at other points during the project. These interviews will be used to gain a deeper understanding into the motivations, perceptions, and priorities of the health care providers regarding service use. The provider IDIs (10 -15 individuals) and FGDs (6 to 8 persons) will focus more specifically on:

- Services offered
- Attitudes towards integration, including effects on workload
- Integrating HIV care and attitudes towards PLWH
- Benefits and challenges to service integration
- Perception of clients’ views in integration
- The referral system
- Other health care needs

At the end of the FGDs and IDIs, participants will be provided with any necessary information to complete their understanding of the nature of the research. The researcher will discuss with the participants their experience of the research in order to monitor any unforeseen negative effects or misconceptions (see FGD section below for detail).

1. Women of reproductive age using FP services

Representative samples of women aged18 years and above receiving FP and other services in all study groups will be followed over a period of 30 months after receiving the service(s). The purpose of recruiting and following these cohorts is to assess whether there are significant changes in clients’ behavior after they have accessed services and whether there are any significant differences between women exposed and not exposed to the interventions. This cohort study will measure a number of behaviors over this period including fertility intentions and their achievement, use of FP, knowledge of and actual HIV status, pregnancy, other SRH and STI/HIV-related behaviors and health-seeking behaviors.

From among the several indicators of behavior that this study will address, the key variable of interest for calculating the sample size is the one for which the largest sample size will be necessary. Findings from the study that preceded this one^[[9]](#footnote-9)^, found that the proportion of revisit FP clients reporting use of condom with a contraceptive method was 2% before the intervention, increasing to 5% after the intervention. On the assumption that the introduction of this intervention will have a similar level of effect, the minimum sample size will be calculated for an increase from 5% to 10%. To detect a 5% percentage point difference the minimum sample size for each cohort from a cluster of six health facilities would be 683. To allow for a 30% loss to follow up, a sample size of 976 would be needed for each cohort. The number of clients recruited from each facility will be proportional to the size of the revisit FP client load for that facility (i.e. the larger the FP clinic, the more women recruited).

The prevalence of HIV among women aged 15 - 49 years in Central Province is estimated to be 7.6% (KAIS 2008). To be able to include a representative sub-sample of HIV-positive women, a minimum of 100 clients who are HIV-positive will need to be recruited into each study group. However we will try to over sample this group of women to ensure numbers are large enough for analysis and allowing for loss to follow up (130). Every effort will be made to ensure that these interviews are undertaken in conditions of strict privacy and that the interviewers will have been trained in maintaining confidentiality.

All women will be asked whether they have had an HIV test, whether they know their status, and if so, whether they would be willing to disclose their status. There will be no pressure to disclose status and unwillingness to do so will not be a criterion for exclusion. This information will enable a comparison of the behaviors among four groups of women to determine whether knowledge of HIV status influences behavior: 1) those who know they are HIV-positive; 2) those who know they are HIV-negative; 3) those who do not know their HIV status and 4) those who are unwilling to disclose their status.

Women will be eligible for inclusion if they meet the following criteria:

- Are aged18 years and above as obtaining parental/guardian permission for legal minors will not be possible at the clinic level
- Are revisit FP clients (new clients problematic to define as ‘new’, numbers are few)
- Live in the catchment area of the health facility so that they are likely to continue to receive services from the same facility and maintaining contact over time will be easier;
- Give their informed consent to be interviewed on recruitment into the study and to be contacted and interviewed at three additional times: six months, 18 and 30 months later.

All women satisfying the inclusion criteria and giving informed consent to participate in the study will be recruited in July 2009 until the required sample size has been reached (see below). Women will continue to be interviewed even if they become pregnant. All women wishing to withdraw from the study at any point will be allowed to do. For the three interviews subsequent to the recruitment interview, women will be offered the option of the researcher meeting them at their home, at the facility (if attending for an FP service), or at a mutually agreeable location. At the time of recruitment, the woman’s name, physical address and a phone number (where available) will be recorded (Appendix 3B). This contact information will be recorded on a sheet separate from the questionnaire and kept physically apart (in a locked cupboard) from the data collection instruments. The contact details will only be linked to the questionnaires by the study ID numbers and only the Principal Investigator will have access to both the contact sheets and questionnaires. Women will be informed that the costs of their travel and light refreshments will be reimbursed if interviewed away from their homes (an average of $5/Kshs350).

Using a structured Kiswahili or English written consent form (depending on their preferred language), all women will be informed about the objectives, procedures, benefits, and risks of the study. Informed consent will be obtained separately for each interview according to the informed consent procedure described in appendices 3A and 3C.

1. Community members living in the catchment area for selected study facilities

LSHTM will conduct a community-based survey with a randomly selected sample of residents living in communities within the catchment areas of the study facilities. The purpose of this survey is to measure community perceptions of the quality and use of FP, STI, HIV/AIDS and other SRH services available not only from the study facilities but also those available from private practitioners (e.g. pharmacists, private nurses and midwives, and through social marketing) and from informal or traditional providers (e.g. traditional birth attendants, traditional practitioners, etc.). Knowing which services are accessed from which sources will enable the provision and use of services from MOH and FHOK services to be better understood.

Information to be collected through the community surveys will include: awareness of services available at the study facilities; preferences for sources of services; perceptions of service quality at different sources; and any stigma associated with delivery of services from different sources. Preferences will be recorded using Likert-type scales. Views on hypothetical situations, such as men attending services previously dedicated to women, will also be asked to address issues of service-associated stigma. Information will also be requested about: marital status/ current partner status; contraceptive use; whether last pregnancy planned; condom use at last sex; and HIV status.

Surveys will be undertaken in Thika town in the catchment areas of the MOH Thika District Hospital and the FHOK ‘Family Care Thika’ clinic and in the catchment area of Nyandarua District Hospital which is in Nyahururu Town. Thika town was selected as two of the intervention facilities are in that town. Nyahururu is the biggest town in the comparison group sites and similar in social- economic characteristics. Surveys will be undertaken in mid 2009 and 2011 to compare patterns of service use and perception and to compare any differences between the two communities. Eligible households will be those containing a man or woman aged 15 – 49 years. Those who are 15 to 17 years old will only be interviewed following parental consent (see appendix 4C). Respondent sampling and recruitment will be at the household level. Since the main purpose of the surveys is to obtain a clear picture of use of services in the respective areas, which is currently unknown, they are therefore largely descriptive initially and, until initial surveys have been completed, the target indicators for change are speculative.

Table 2 therefore contains indicative numbers showing the approximate magnitude of indicator change possible, to detect with 95% confidence, at a practical and manageable sample size of 800 women and 800 men. The sample sizes given are based on potential changes between the surveys for the example indicators listed in the table, assuming 80% power and a design effect of 2.0.

**Table 2**: **sampling for community survey**

| **Indicator** | **Current estimated level** | **Potential change**  **to:** | **Sample size**  **(80% power,**  **DEFF=2)** |
| --- | --- | --- | --- |
| Proportion of eligible women who have ever used index* clinic | 50% | 60% | 816 |
| Proportion of women who can list the RH/HIV services available at index clinic | 30% | 40% | 752 |
| Proportion of eligible men who have ever used, or would use clinic for sexual health services | 10% | 17% | 802 |
| Proportion of eligible women using multiple services rather than an integrated service | 30% | 20% | 616 |
| Proportion of sexually active unmarried women not using contraception | 54% | 64% | 798 |
| Proportion of men willing to use predominately female-oriented services | 20% | 30% | 616 |
| * index clinics are the MOH Thika district hospital, FHOK Thika clinic and the Nyahururu district hospital | | | |

Census enumeration areas (EAs) for the towns of Thika and Nyahururu will form an initial sampling frame. EAs will be excluded if they do not fall within the facility catchment areas. Due consideration will be given to stratification of EAs based on accessibility and known socio-economic levels. Sampling will be in clusters, such that pairs or small groups of interviewers can spend one day interviewing sufficient numbers in one cluster. To this end, the actual number of clusters (at least 20) will be identified by systematic sampling, with probability proportional to EA population size. Starting points for clusters within EAs will be identified by random selection using map reference coordinates. Interviewers will be instructed in random walk techniques radiating from the selected starting point to identify potential households.

On arrival at a household, a short introduction will be made to the head of the household and if there is agreement to participate a short household listing will be completed recording the sex and age of all household members present at time of visit. Where there is more than one eligible person the interviewee will be selected by a prepared Kish grid. If this person declines to be interviewed another eligible person in the same household may be recruited, again using the Kish grid. When one person has agreed to be interviewed, a more detailed introduction will be made explaining the study and obtaining written informed consent using the procedure given in appendix 4A. We will not interview men and women from the same household, therefore the response from a single household may be a man or a woman, but not both.

Data will be entered into a purpose-designed Access database in Population Council Nairobi office by LSHTM recruited data entry clerks. Using unique identifier numbers and cluster numbers but with no name or address information. Paper copies of all completed questionnaires will be retained in the Population Council’s office under lock and key.

**In-depth interviews and focus group discussions with community members**

In order to enhance the findings from community surveys and address unforeseen questions arising from other components of the study, LSHTM will carry out in-depth interviews (IDIs) and focus group discussions (FGDs) with groups of male and female clients and non-clients (aged 18 years and above) as well as key informants. These will take place alongside the community surveys, as well as on an *ad hoc* basis when needed at other points during the project.

Each Biographic Narrative Interpretive Method (BNIM) interview will consist of 3 sub-sessions. In the first sub-session, the interviewer will offer only a carefully constructed single narrative question to illicit an extensive, uninterrupted narration. In the second sub-session, the themes and stories to be elaborated upon are based upon the Gestalt of the first and therefore reflect the ordering of themes and the same words that the participant had used in sub-session 1. In the third sub-session, more focused non-narrative questions will be posed. Typically, sub-session 1 and 2 blend together into a 'first interview' and sub-session 3 is a 'second interview'. However, in order to avoid the need for the participant having to come back for a second interview, the 3 sub-sessions will be combined into 1 interview lasting approximately 1-2 hours.

These interviews will be used to gain a deeper understanding into the motivations, perceptions, and priorities of the local community regarding service use. Snowball sampling through key informants and the community surveys will be used to recruit participants. The IDIs and FGDs will address the following broad themes:

- Motivations for health care use and selection/use of the particular service, including general community perceptions / rumors regarding different available services
- Attitudes towards integration
- Communication/interaction with the provider
- Attitudes towards integrating HIV and SRH, including willingness to attend services known to provide HIV care and service-related stigma.
- Contraceptive and sexual health behaviour, including communication with partners and other community members about care-seeking decisions

Interviews will only be recorded after obtaining written informed consent from the interviewee (see Appendix 5). From the outset, it will be made clear to participants that they have a right to withdraw from the research at any time. All personal data will be treated confidentially. Data stored on computers and associated hardware will be password−protected. Hard copies of questionnaires, anonymised transcriptions and interview tapes will be stored securely in a locked cabinet for no longer than 10 years after the study has ended, in accordance with the UK Data Protection Act 1998 and the LSHTM Data Protection Policy. At the end of the FGDs and IDIs, participants will be provided with any necessary information to complete their understanding of the nature of the research. The researcher will discuss with the participants their experience of the research in order to monitor any unforeseen negative effects or misconceptions. Participants will receive an honorarium payment of $5 for any out-of-pocket expenses associated with taking part in the FGDs.

***Focus group discussions***

To determine local perceptions and support for the services, and acceptability of integrated services, FGDs will be held with groups of both male and female non-users as well as with providers. The non-client and provider focus group consent forms are in appendix 5 and information sheets and question guides can be found in annex 6. FGDs of between 1 and 2 hours duration will be held in 2 districts:

*Thika District*

- 1 FGD: 6-8 younger women who are currently non-service users (<25 years)
- 1 FGD: 6-8 older women who are currently non-service users (25 years and above)
- 1 FGD: 6-8 younger men who are currently non-service users (<25 years)
- 1 FGD: 6-8 older men who are currently non-service users (25 years and above)

*Nyandarua District*

- 1 FGD: 6-8 younger women who are currently non-service users (<25 years)
- 1 FGD: 6-8 older women who are currently non-service users (25 years and above)
- 1 FGD: 6-8 younger men who are currently non-service users (<25 years)
- 1 FGD: 6-8 older men who are currently non-service users (25 years and above)

***In-depth interviews***

To avoid preconceived ideas about the experiences of using different types of providers, Biographic Narrative Interpretive Method (BNIM) interviews^[[10]](#footnote-10)^ will be conducted with:

*Thika District*

- 15-20 female service users
- 5-10 male service users
- 3-5 nurses / counsellors
- 3-5 doctors / health service managers

*Nyandarua District*

- 15-20 female service users
- 5-10 male service users
- 3-5 nurses / counsellors
- 3-5 doctors / health service managers

# Research Protocol

This study uses three separate designs and is presented diagrammatically below:

**INTERVENTION**

Year 1 Q1 Year 1 Q3 Year2 Q1 Year3 Q1 Year 4 Q1

Final

Health Facility Assessment in all sites

Intermediate Health Facility Assessment in all sites

Pre-intervention

Health Facility Assessment in all sites

Immediate post intervention

Health Facility Assessment

MOH Intervention sites only

Community interviews in two locations

**Design 1**

Community interviews in two locations

2^nd^ interview

(6 months)

Cohorts recruited from: 8 MOH facilities

1^st^ interview

3^rd^ interview (18 months)

4^th^ interview (30 months)

**Design 2**

**Design 3**

Monitoring data collection from 12 MOH and 6 FHOK facilities

Regular supportive supervision visits to 6 MOH and 3 FHOK experimental facilities

Periodic economic data collection from 12 MOH and 6 FHOK facilities

**Description of intervention**

*Study design 1*: Quasi- experimental time series assessment within facilities

*Study design 2*: Static post test comparison following experimental and control cohorts of clients

*Study design 3*: Post test only community perceptions among experimental and control communities Intervention

In conjunction with MOH, evidence from the previous study (Wilson et al 2008) and results of the quality of care assessment in January 2009, the integrated package of care will be reviewed, revised and adapted to improve the services provided. It is expected that at every FP visit clients risk behaviour is assessed and they will be offered CT for HIV. In addition the linkages to CCCs for those HIV positive clients requiring further management and antiretroviral drugs will be strengthened and monitored. All procedures and protocols will follow the national guidelines.

**Table 3: package of integrated HIV/SRH care**

| **Package of integrated care HIV/FP/ART** | **Care required according to status** |
| --- | --- |
| ***All FP clients***  Counseling on FP methods  Counseling on side effects of FP methods  Screening and management for STI  Counseling for HIV  Testing for HIV  Referral for further services | ***HIV negative clients***  IEC  Risk factor exposure  Risk assessment (routine) |
|  | ***HIV positive clients***  CD4, Haemogram, SGPT/ALT creatinine  Clinical staging  CTX prophylaxis  Monitoring x 3 - 6 monthly  Opportunistic infections  Adherence counseling  Counseling on side effects |

Following meetings with relevant stakeholders’ consensus will be built around a series of interrelated interventions likely to be instituted at the study sites to maintain the provision of integrated family planning and HIV services. The intervention will build on the previous study and take place in the six selected facilities, Activities for the comparison facilities will include ensuring sufficient drugs, FP commodities and HIV testing kits are available as well as improving data collection and recording. Training in formal ‘integration’ of CT/FP for the comparison facilities will take place towards the end of the project period.

The intervention will include the following components:

- - Development, adaptation or updating of guidelines and protocols (where necessary)
  - Development of an appropriate training and monitoring/supervisory package
  - Facilitative supervision;
  - Improving provider capacity including technical skills for long term FP, HIV counseling and testing, HIV services, Screening for STIs and syndromic management of STIs
  - Role clarification with all staff working at intervention sites
  - Ensuring availability of minimum levels of equipment and supplies required for providing integrated services
  - Improving the availability of IEC/BCC materials
  - Strengthening data collection and recording systems.
  - Organizational change of how services are provided
  - Strengthened referral system between FP clinic and ART centre

The intervention will be evaluated as follows:

1. Measuring quality of FP and other services over time

Whether facilities can change the quality of care provided following an intervention to integrate services, whether these changes can be sustained over time, and whether external factors can also influence the quality of care provided, are all key issues of interest to stakeholders. Population Council and LSHTM will address these issues by conducting four health facility assessments^[[11]](#footnote-11)^ (HFAs) of the quality of care provided in both MOH and FHOK study facilities.

An initial pre-intervention assessment will be undertaken in both experimental and comparison MOH facilities immediately before the interventions are introduced to determine the comparability of the facilities and to provide baseline measures of the quality of care. An immediate post-intervention assessment will be undertaken at five months after the interventions are introduced, in the experimental facilities only, to determine the extent to which the quality of care has changed. Five months is sufficient time for the new service configurations to have “settled in” and become routine procedures. This second assessment is critical as if there is no programmatically significant change in the quality of care provided then the cohort studies will not be undertaken. If the change is sufficient to justify continuing the study, the measures of quality from this assessment will be assumed to represent the quality of care received by the women recruited into the experimental cohort. The measures of quality of care in the comparison facilities during the pre-intervention assessment will be assumed to represent the quality of care received by the women recruited into the comparison cohort.

To determine the sustainability of the quality of care provided in the experimental facilities and to assess whether external factors may be influencing quality over time, a third ‘intermediate’ HFA will be undertaken at 18 months and a final HFA undertaken at 30 months, in both experimental and comparison facilities and the FHOK facilities^[[12]](#footnote-12)^. In addition, data collected through routine monitoring of service statistics (see below) will provide further information about the sustainability of the new service configurations in terms of client load, services mix, client characteristics etc.

Moreover, Population Council and LSHTM staff with MOH (Kenya) or MOHSW/CSO and FLAS (Swaziland only) will carry out routine support supervision staff to observe the way in which services are being delivered and allow for occasional adjustments, including refresher training (in both M and E and technical skills updates) as needed.

##### Data collection

Data collection procedures for each component of these assessments are as follows:

**Facility Inventory:** An inventory of available resources to learn about the facility infrastructure, staffing numbers and skills mix, services provided, staff training undertaken, availability of equipment, commodities, test kits, stationary (client cards and notes), ARV drugs and other medications required to provide the services within the intervention will be undertaken. The head of the facility will be approached by a nurse/midwife researcher. The researcher will request the in-charge to guide the N/M around the facility to observe and record all relevant information on a checklist.

**Review of service statistics** (related to routine programme data) regarding utilization of family planning and HIV services for a 6 or 12 month period prior to the assessment visit. We will also record the number of new and continuing clients coming to a clinic for FP services as well as other health services. Monthly trends in the numbers of new and continuing family planning clients as well as for other services will be obtained from clinic records.

**Interviews with health care providers** to determine their knowledge and skills for CT/FP/STI, HIV services, as well as the understanding of the organizational setup and description of related activities. Interviews will also ascertain their perceptions of barriers and operational challenges that may influence clients’ acceptance of counseling and testing for HIV and FP, and the provider’s attitudes towards the changes in policy and procedures needed to provide the new integrated consultation. All providers at the MCH-FP and ART units will be approached for interview in the 12 health facilities. We anticipate between 3 and 6 providers from each facility will be interviewed.

**Observe client-provider interactions (CPI) during FP consultations** The CPI encompasses both the **process** (how clients are treated and whether they actively participate) and the **content** (what they are told, technical competence, accuracy of information, provision of essential information) of a consultation. After obtaining informed consent from the client, a structured non-participatory observation of the client-provider interaction will be undertaken to determine the quality of care provided. Subsequent sessions for which consent has been received will be observed until 18 randomly selected new FP clients and 18 revisit FP clients in each facility has been observed. This includes both GOK and FHOK clinics.

We acknowledge that observing client provider interaction may bias in a positive direction the results obtained on quality of care. We will be spending more than one day at each site, so that the presence of the research team becomes more familiar and the behavior of the providers becomes more normative.

**Client exit interview:** Exit interviews (with informed consent) will be held with each client who was observed by a trained interviewer to ascertain their perceptions of the service received, which will be used as the indicator of acceptability to the client. A code on a piece of paper will be given to the client as she leaves the consultation and asked to give to the interviewed outside for a short follow up interview. This will take place before the intervention after the intervention in the intervention facilities and periodically over three years. Additional client exit interviews may be conducted (by LSHTM) in order to ensure a large enough sample size per clinic to observe significant change over the intervention period in key behavioral and service use indicators, such as condom use and testing incidence.

To measure the magnitude of changes in the quality of services provided, composite summary scores will be developed for a series of key indicators by aggregating the mean scores of key items being assessed for each individual client-provider interaction being observed. This scoring system will categorize whether an accepted standard of quality has been met or not. For each study group, a mean score per group will be calculated for each indicator and for the composite summary score to enable statistically comparisons to be made between experimental and comparison groups and over time. Examples of the types of individual items and key indicators that will be used are given in the table below.

**Table 4: Groups of key actions/indicators to make composite scores of quality of care**

| **Quality of:** | **Observed provider actions:** |
| --- | --- |
| 1. FP method counseling (0-6) | Discuss reproductive intentions, discuss previous use of FP, Discuss 2 or more methods, provide choice regarding preferred method, discuss how chosen method works, explain (dis)/ advantages of chosen method |
| 1. Client – provider rapport (0-7) | Client greeted warmly, Discussed medical conditions, Asked if client understood information, Encouraged client to ask questions, Used client's name, Help in decision-making, Consultation time > 15 minutes |
| 1. STI prevention counseling (0-5) | Discuss client history of STI symptoms, discuss number of sexual partners, discuss STI, discuss STI risk factors, tells client STI increase risk of HIV |
| 1. Dual protection counseling (0-5) | Explains condoms protect against STIs/HIV and pregnancy, encourages condoms with another FP method, explains how to use a condom, emphasize consistent condom use, explains how to negotiate condom use |
| 1. HIV counseling for unknown or negative status (0-5) | Discuss HIV sero-status, discuss HIV testing with client, discuss interpretation of results, explain about the window period, advises where client can get tested. |
| 1. HIV testing   (0 -7) | Provider explains procedure, conducts test, assists client to interpret his/her results, counsels client about status, discuss disclosure, discuss partner testing, identifies sources of support, |
| 1. Counseling on referral to ART clinic   (0 -4) | Provider explains further blood tests will be required (eg CD4 count), explains about the availability of ARVs, asks client preferred site for ART clinic, gives client referral letter to ART/escorts client to ART clinic |

The proportion of women receiving an acceptable quality of service will be calculated in addition to the mean scores. This is because the mean score may be artificially high if a small proportion of clients receive excellent services. The methodology to calculate the proportion of women receiving an acceptable quality is similar to the Lot Quality Assurance Sampling (LQAS) approach that has been used in Kenya and elsewhere for assessing quality^^[[13]](#footnote-13)^^. LQAS follows the principle that an entire group (lot) of services is deemed poor quality if a certain proportion within a small sample does not reach a minimum standard. LQAS applies cumulative probabilities calculated with a binomial formula to select small sample sizes and decision criteria for judging a group of providers.

In consultation with the Ministry of Public Health and Sanitation (Division of Reproductive Health and National AIDS STI Control Program) and Ministry of Medical Services (Division of Obstetrics and ART Program)^[[14]](#footnote-14)^ the minimum standard for quality integrated CT/FP services will be developed by building on existing guidelines and protocols and previous studies. Valadez et al (1997) used LQAS to assess service delivery skills of providers trained in a new FP curriculum. The standard used to assess adequacy was that at least 85% of the group of providers retained the family planning service delivery skills learned in training. Each provider was judged as having retained a particular skill if in six observed observations of the task no more than one performance error occurred.

1. **Prospective routine monitoring of clients accessing services over time**

All research sites (MOH and FHOK clinics) will undergo technical assistance to strengthen collection of routine program data, to ensure information on client profiles and their use of different services is regularly compiled and synthesized. A two day workshop will be held for representatives from all study sites and the provincial HMIS and RH/HIV teams. This workshop will include the following: the role of monitoring & evaluation, and how they relate to both project management and assessing outputs; evidence required to establish different levels of certainty about how/ whether projects are having their intended outcomes; useful ways to compile, present and use routine data; data required from intervention and comparison arms; compilation of data for the study and how it will be used.

Simple forms will be introduced for extracting client data from the existing registers. This data will be entered monthly into a database for further analysis.

Analysis of routine program data will contribute to answering the following questions:

- What is the change in client profile pre- and post-integration (for instance, in terms of age, and sex)?
- Does the rate of new clients accessing the services increase post-integration?
- What are the changes in service uptake and client-profile pre- and post-integration across a range of SRH/HIV services

Program monitoring data will complement data from facility-based assessments by demonstrating trends in client profiles over time, thus providing information on which the sampling strategy for recruiting clients into CPIs and exit interviews can be based, to ensure representativeness in terms of age, sex, and primary service sought.

1. Costing of service delivery across the different models

In each of the clinics the incremental costs of delivering integrated services will be ascertained and the potential for efficiency gains through the integration of SRH and HIV services will be examined. These will be measured in each setting, and compared with other models of integration and the estimated costs of providing vertical services. The findings will be used to explore whether there are economies of scope associated with the provision of an integrated package of SRH services, and to explore the relative efficiency of different models of provision.

The economic component will be undertaken in a number of steps or sub activities. First there will be a periodic activity review, where the activities and their outputs will be documented annually. This will provide the intervention inputs and provide a picture of how services are evolving over time. This tool is presented in Appendix 5. Building on this, the cost analysis will look at the quantity of resources that are used to implement activities at facility level. These tools will be developed from the first round of results of the periodic activity review, but will generally be based on UNAIDS costing guidelines: *The Costing Guidelines for HIV Prevention Strategies^[[15]](#footnote-15)^.* The analysis of resource requirements will use the results of the cost analysis to model the cost under different circumstances, such as when projects scale up or more services are integrated. Lastly, the detail of the cost analysis will be used to conduct an econometric analysis to estimate the impact of different cost drivers empirically. This is the first time an economic study of this type will be used to evaluate integrated services.

1. Cohort study of clients

After the post-intervention quality of care assessment has been completed, Population Council will recruit and follow two samples of clients accessing FP services over a period of two and half years from both the intervention and matched control facilities that are not providing the integrated package of services. Revisit FP clients will be interviewed over time to measure the following indicators: fertility intentions; use of FP; HIV status (known, unknown) pregnancy (planned and unintended), other SRH behaviors. While every woman recruited at the MCH-FP represents a potential participant, distinctions will be made between women; by virtue of their HIV status.

Given that majority of FP clients use injectables or oral contraceptive pills, most women recruited into the study will be expected to make a visit to the clinic after six months for either the two or three month injectable or a supply of oral contraceptives. Consequently, field workers will be based at the clinics full time once the six month follow up visits are due to allow follow up in the clinics for the mornings and home visits for clients who require follow up at home in the afternoons. The follow-up interviews can take place at any time before contact with a health provider or after the client has received services for the day.

Women on a method that does not require a return visit to the clinic at six months, or those who have not returned to the clinic for their follow up visit within one week after their visit, or women who went to another clinic for re-supply, or women who discontinued using FP for whatever reason, will be contacted by telephone and/or home visit and an appointment will be scheduled, either for her to visit the clinic or for an interviewer to visit her at home. As highlighted above, the majority of women will come back to the clinic for follow up services, but it is expected that approximately 20-30 percent of women will require follow up at home.

If the woman is not located after three attempts she will be considered lost to follow up. If a woman chooses to be seen elsewhere for a confidential interview a suitable time and place will be arranged. A client database with the expected date for six-month follow up will be maintained to indicate which women have not been followed up. The field worker stationed at each clinic will be responsible for making any home visits required in the vicinity of their clinic in the afternoons. The study coordinator will be responsible for supervision of all field workers/data collectors.

Social scientists will be trained to interview the FP clients. To assist in tracing the women after six months, we will request the district MOH to assign six Public Health Technicians (PHT) to work with the researchers; PHTs are issued with motorbikes to carry out their work in the community and are therefore able to carry the researchers around the communities (all will wear safety helmets). Their knowledge of the local area and the availability of their motorbikes, have proved invaluable in finding the women’s homes in other studies. In many parts of Central Province there is very limited public transport once off the main tarmac roads and the topography is very hilly.

Given that the average monthly client load of revisit FP clients for the two clusters of health facilities ranges from approximately 88 clients to 529 clients we anticipate approximately four to six weeks will be needed to recruit all women for the two cohorts. Therefore two research assistants will recruit women from the health centers (8) and four research assistants will be attached to the hospitals to recruit women at the FP clinics.

1. Community survey

LSHTM will conduct community-based surveys with a randomly selected sample of men and women aged 15-49 years from the catchment communities of Thika town and Nyahururu town. All adolescents 15 – 17 years will only be interviewed following parental consent. The intention of the community surveys is to build an unbiased and independent view of the use of services in the respective communities. Services will be taken to mean any FP or HIV or STI related service, including informal or traditional services. A picture of the use of services will be invaluable in understanding the options available to the target population.

Initially two surveys will be executed, one in the catchment area of Thika District Hospital and the nearby FHOK clinic known as Family Care Thika, and one in the catchment area of Nyahururu District Hospital. These clinics form the *index* clinics. A second pair of surveys will be executed in the same areas in 2011, after the period of service strengthening has occurred in the Thika area. Both pairs of surveys will be cross-sectional.

Although comparisons between the disposition and use of services in the two areas following the first survey round will be useful the prime use of these surveys will be to compare patterns of use between first and second surveys and compare changes between the two communities before and after the intervention period.

Community members will be asked standardized, and sex-specific, questions on access and use of services, attitudes, stigma, experiences and reasons for service use/non-use, as well as on unmet need for HIV testing and family planning. This will offer a comparison between the exit client and the cohort study to give an indication of differences in characteristics, behaviour and attitudes of clients *vs* non-clients, as well as offer insights into preferences for vertical or integrated services and reasons for use/non-use of these. Views on hypothetical situations, such as men attending services previously dedicated to women, will also be asked to address issues of service-associated stigma.

Use of service facilities is then recorded on a grid, with information about fees paid. Selected facilities will be pre-printed onto this grid after advice from the relevant district management team. Additional sheets will ask further details about the use of individual services identified through the use of the service record grid.

**In-depth Interviews and Focus Group Discussions**

Each Biographic Narrative Interpretive Method (BNIM) interview will consist of 3 sub-sessions. In the first sub-session, the interviewer will offer only a carefully constructed single narrative question to illicit an extensive, uninterrupted narration. In the second sub-session, the themes and stories to be elaborated upon are based upon the Gestalt of the first and therefore reflect the ordering of themes and the same words that the participant had used in sub-session 1. In the third sub-session, more focused non-narrative questions will be posed.

With the goal of discovering meaning and achieving understanding through the narratives of lived lives, the emphasis or the interviews will be on analytic induction^[[16]](#footnote-16)^ allowing for modification of concepts and relationships between concepts. The process of analysis will be initiated after the first interview/s from which a tentative hypothetical framework will be generated. Comparisons will be made with additional forthcoming data resulting in changes in the framework until a group of ‘recursive rules’ are developed that comprehensively describe the phenomenon of experience of sexual health care and family planning services. Thematic analysis will be performed manually and using QSR NVivo. A reflective team approach will involve narrative microanalyses of selection of meaningful text upon which hypotheses and associations might be made. The text chosen for analysis will represent shifts in the modes of narration by the interviewee or potential themes and their development. Using a ‘reflecting team’ approach to hypothesize and develop themes opens up other possibilities in interpretation, rather than relying solely upon the primary researcher’s interpretation of the interview. The FGD transcripts will be analyzed thematically.

**Table 5: Operational results and indicators**

| **1. Determine the feasibility, acceptability, cost and immediate effectiveness of providing expanded package of integrated services to new and revisiting FP clients** | | |
| --- | --- | --- |
| **Results** | **Indicators** | **Data source** |
| Feasibility of linking services demonstrated | Number of internal and external referrals  Number of staff updated in C T skills  Facilities have adequate equipment and supplies  Number of staff updated in HIV BCC prevention | Service statistics  Provider interviews  Facility inventory |
| Provision of linked services reported as acceptable by providers and clients | Clients received CT in FP consultation  Clients referred for ART  Clients referred for other services | Client exit  Provider interview  Cohort |
| Increased % of eligible HIV+ women starting to use ART | Improved referral mechanisms  % clients know their CD4 counts | Service statistics  Cohort |
| Increase in clients using integrated CT, FP and STI screening services | % FP clients accessing more than one service | Service statistics  Exit /Cohort |
| Increased numbers of clients screened/managed for STIs | % FP clients accessing separate/integrated services | Service statistics  Cohort |
| Increased in new and repeat FP clients testing for HIV |  | Service statistics  Cohort |
| Improved attitudes of service providers towards HIV+ clients | % Providers indicating non discriminatory attitudes  % Clients recommending services to others | Provider interview  Cohort survey |
| Total resource requirements and unit costs of intervention | Cost per eligible client receiving ART,  Cost per person-month of receiving ART  Cost per client counseled, tested and receiving results  Cost per client receiving each service component | Economic study |
| **2. Determine impact of integration on client behaviors and their health status** | | |
| **Results** | **Indicators** | **Data source** |
| Reduction in reported HIV risk behaviors among HIV negative & HIV positive clients | Condom use at last sex;  Number of partners in past 12m  STI /HIV counseling  Condom/dual protection | Cohort  Client Exit |
| Reduced incidence of unintended pregnancies | % women who become pregnant 0 % planned pregnancy  % clients with correct knowledge of fertile period | Cohort  Client Exit |
| Increased duration of contraceptive use among HIV negative & HIV positive clients | Ever/Current use of FP method  Discontinuation FP rates in 12 months  Ability to achieve fertility goals | Cohort survey |
| Increased compliance of clients on ART treatment | Reduction in number of opportunistic infections  Reduction in missed pill days | Cohort survey |
| Improved attitudes of service providers towards HIV+ clients | Proportion of providers indicating non discriminatory attitudes towards HIV positive clients | Provider interview |
| % clients reporting unacceptable stigmatizing behavior by providers | Clients reporting positive experience of CT process | Community and  Cohort survey |
| Decreased stigmatization at community level of HIV services if integrated with FP RH services | Perceived barriers to accessing services: costs, distance, quality, waiting times, stigma surrounding service | Community survey  Cohort survey |

**Risks:** There are a few risks involved with conducting this study. During the recruitment interviews at the facilities, women will be asked a number of potentially sensitive questions, including their HIV status and their perceptions. Some of which might be regarded as intrusive and too personal. Therefore, careful steps will be taken in the questionnaire design to minimize potential discomfort to our informants. The study tools will also be pre-tested among a small group of women with similar characteristics as the study population to identify potentially negative consequences and modified accordingly*.* To avoid the risk of others overhearing this information, interviews will be conducted in strictly private settings, and ample time will be allowed for data collection to ensure that privacy and confidentiality can be guaranteed.

While we have used the label “women who are HIV positive” in this proposal, extreme caution will be taken during fieldwork NOT to single out our informants as such. Instead, throughout the study, they will be simply referred to as ‘clients’ in order not to reinforce stigma.

Provisions will be made to train researchers to ensure that guidance on ethical conduct is clearly understood and implemented. Such training will include focused sessions and exercises regarding the meaning and process of informed consent, the importance of protecting the privacy of subjects, and confidentiality of the information obtained from them. The research team will also be trained to listen and observe intently without displaying any judgmental attitude towards information they receive from the informants and on other critical ethical issues on gathering information from the women.

**Benefits:** No immediate tangible benefit is likely to accrue to the subjects through their participation and this will be made clear when obtaining informed consent. However, the potential benefits to health care services and the women who use them of conducting this study will be described to the potential participants, so that they are fully aware that the data gathered will be used to provide recommendations to the Kenya Ministries of Health, as well as to health care providers and communities in other countries. Some of our informants may report problems that will require medical and or psychosocial attention. Our informants will be referred to the existing services available in the facilities. This will be made clear when obtaining informed consent.

If the researchers find out information or observe activities during site visits that reflect poorly on the quality of health services provided or the health system in general, this will be recorded and incorporated into the report. The intervention team will try to address these issues (e.g. training of health care providers will include interpersonal skills as well as clinical skills). If, during a client/provider interaction, a client is perceived to be at medical risk, the observer (who will be medically trained) will be told to intervene.

**Confidentiality:** Given the sensitive nature of the information to be gathered, protecting and respecting the confidentiality and privacy of our informants will be a critical consideration throughout the study. We intend to use individual in-depth interviewing in most cases in order to provide each informant with a greater assurance of confidentiality. The research teams will discuss and develop methods and procedural measures in relation to matters such as data recording style, personal identifiers, transcription and processing procedures, lifespan of unprocessed data, type and places of storage, and data safety and right of access. This will be developed prior to the onset of the research study. All data will be kept separately from identifying information and will be stored under locked files. Access to data will be strictly limited to the research team. All interviews will be conducted in private. Identifying details will not be included in the results presentation. Confidentiality will be maintained at all times through training interviewers, the meaning of confidentiality and ways of maintaining confidentiality during data collection and afterwards. Researchers conducting FGDs will be reminded how to preserve confidentiality and ask the groups to respect personal information among the groups before discussions start.

All data collection instruments will be kept under lock and key at all times in the field (in locked boxes at the facilities) and in the Population Council’s Nairobi office (in locked file cabinets). All data collection instruments will contain a study ID number. Names will only be recorded for women agreeing to the follow-up visits to facilitate contact. This information, together with their contact information, will be recorded on a separate sheet and kept physically separate from the data collection instruments containing their information, and will only be linked to questionnaires by study ID numbers. Only the research team coordinator will have access to both the names and instruments.

According to Kenyan law, the age of consent for participating in research for both male and female informants is 18 years. Given that it would be difficult to trace parents of adolescents attending FP clinics we will not interview anyone under 18 years of age. However for the community survey any adolescents aged 15 – 17 years will be interviewed at the household level only if parental consent has been obtained first.

Data collectors/field workers will be asked to work where they are not known personally, to respect privacy and ensure respondents’ anonymity. Results of client and provider interviews will be presented in reports in an aggregated manner such that responses cannot be traced back to individuals. Population Council staff will visit the data collection site to ensure interviewers’ adherence with confidentiality procedures.

As part of Population Council’s monitoring program, the subjects participating in the research will be asked to acknowledge the possibility that an interview may be requested by a representative of Population Council to determine if informed consent occurred. Following a request for an interview, the subject will have the option of accepting or declining the interview.

**Compensation:** During follow-up interviews, a woman may not want others to know that she is participating in the study, and so we will offer all women the opportunity to hold the interview in a location of her choosing, and will reimburse travel costs to ensure that this is a viable option. The majority of clients will be interviewed at the clinic when they come for FP services. However where our informants are requested to travel specifically for interview, the study will compensate informants (at an average rate of US $5 per study participant) for this inconvenience by covering the cost of a meal/snack and also providing transport refund to attend pre-arranged interview meetings.

# Informed Consent: Written informed consent will be obtained for every data collection activity: see Appendix for all informed consents forms that will be used in this study. Prospective study participants will be provided with information about the study before any consent to participate is sought. Participants will be informed about the requirements for participating in the study, including follow up. Information will be read to the participant who will be expected to verbally agree and sign the informed consent sheet.

Participants will receive the following information:

- Aim of the study and methods to be used
- Institutional affiliations of the research
- Anticipated benefits and potential risks and follow-up of the study
- Discomfort it may cause
- Sensitive questions regarding sexual behavior, partners and condom use will be asked, though they may choose not to answer any questions
- Questionnaire administration will increase time at clinic
- Right to abstain from participating in the study, or to withdraw from it at any time, without reprisal
- Measures to ensure confidentiality of information provided
- Study numbers will be used on questionnaires to maintain anonymity of study participants
- No information will be divulged to partners or other third parties
- Monetary compensation will only be provided if participant has to travel for the interview
- Contact details of the study coordinator for any questions or concerns

***Ethical clearance:*** The research protocol will be submitted to the following for approval:

1. Division of Reproductive Health and NASCOP, Ministry of Public Health and Sanitation
2. Kenya Medical Research Institute (KEMRI) Ethical Review Board
3. Population Council IRB
4. LSHTM Ethical Review Board

CV summaries for key research personnel are in Appendix 6 and ethical training certificates in Appendix 7 (Pop Council staff):

**Population Council**

Ian Askew

Charlotte Warren (PI)

Annie Mwangi

**LSHTM**

*Centre for Population Studies*

Andy Sloggett acting PI

(Susannah Mayhew (PI))

Joanna Busza

Natalie Friend-duPreez

*Economics team*

Charlotte Watts (PI)

Lilani Kumaranayake

Fern Terris-Prestholt

All questionnaires (final drafts) are attached in the Annex

# Appendix 1: Consent for provider interview

**Assessing the benefits and costs of integrating HIV into reproductive health /postnatal services in Kenya and Swaziland**

**Interviewer:** Introduce yourself to the provider: Good morning/afternoon. My name is________.

I am working with Population Council an international non profit organisation, the London School of Hygiene and Tropical Medicine, UK, International Planned Parenthood Federation (an international non profit organisation) and Ministry of Health/Ministry of Health and Social Welfare to understand the issues around the integration of HIV and RH services. The Principle Investigators are Charlotte Warren from Population Council, and Susie Mayhew and Charlotte Watts from LSHTM.

**Purpose:** The purpose of this study is to explore factors around the delivery of HIV and RH services. We are collecting information from clients using the services. We would like to learn about how services are organized and provided and to listen to your opinions about integration of services and referral process. I would like to sit down with you and ask you some questions. This will take approximately 30 minutes. I will be writing on this form. It does not have your name on it. The risk of participation is that you may feel uncomfortable talking about your job.

**Benefits:** The results from this project are expected to contribute towards understanding integrated services. The benefit is that what you say is important and valuable. The results of this study will be used to improve formulation of policy and the delivery of services. The information you give us will be used to provide recommendations to the Kenya Ministries of Health as well as to health care providers and communities in other countries.

**Risks:** We believe that this study is safe and do not expect you to suffer any harm or injury because of your participation in it.

**Confidentiality:** The information you provide us is confidential. It will not bear your name and will be kept under lock and key. No one else working in this facility, including your supervisor(s), will know what you say. Please note that the data will be collected and your DHMT and community will receive feedback. Thus the overall information and opinions will be revealed but it will not be known who said what.

Participation in this study is voluntary. You may refuse to answer any question. You may choose to stop the interview at any time. Refusing to participate will not affect your job or position in any way.

As part of the monitoring program, someone may ask to talk with you again to check if I have completed this informed consent. They may ask questions about the risk, benefit, and procedures in this study. If an interview is requested, you have the option of accepting the interview or declining the interview. Again, all information will be kept confidential.

**Subject’s Statement**

*“I have read or have been read the above considerations regarding my participation. I have been given a chance to ask any questions I may have and my questions have been answered to my satisfaction. I understand that the information I give will be kept private. I understand that I may withdraw from this study at any time. My withdrawal from this study or my refusal to participate will in no way affect my job or position. I agree to participate in this study as a volunteer”*.

Do you have any questions? Yes No (If yes, note the questions)

----------------------------------------------------------------------------------------------------------------------

If you have any other questions please ask for:

Annie Mwangi at Population Council Nairobi Tel: +254 020 2713480 (Kenya)

If you have any questions about participating in the research please contact KEMRI 020 2722541 for Kenya

Would you be willing to be in the overall study: Yes No (If NO, Thank provider and end interview)

------------------------------------------ ---------------------------------------------

Provider’s signature Date

# Appendix 2a: Consent for client to observe provider-client interaction

**Title of RESEARCH project: Assessing the benefits and costs of integrating HIV into reproductive health /postnatal services in Kenya and Swaziland**

**Interviewer: Introduce yourself to the provider:** Good morning/afternoon. My name is________________. I am working with Population Council an international non profit organisation, the London School of Hygiene and Tropical Medicine (LSHTM), UK, International Planned Parenthood Federation (an international non profit organisation) and the Ministries of Health to understand the issues around the integration of HIV and RH services. We would also like to learn how family planning and HIV/AIDS prevention and care services are provided to women who come for RH and HIV services. The Principle Investigators are Charlotte Warren from Population Council, Susie Mayhew and Charlotte Watts from LSHTM.

**Study procedure**

Participation in this study will involve observing you and your provider as you interact during the consultation. With your permission, I will sit in the corner of the room and will listen and observe your interaction with the provider. I will write down my observations on this form, which will not bear either your name or that of your provider. I am told that the consultation will not take more than 25 minutes. Following the observation we would like to request you to be interviewed by a colleague before you leave the facility. You will not receive money or reward of any kind if you agree to be interviewed.

**Benefits of participating in the study**

The benefit is that what you say is important and valuable for guiding the Ministries of Health in improving services for women in Kenya.

**Risks of participating in the study**

During consultation you may share with your provider some information, which is of personal nature. I understand that this is part of the normal process but it could be uncomfortable for you if I am present. If at any time you feel you cannot continue with the consultation in my presence please feel free to ask me to leave. I will be recording information about the nature and way in which the provider offers services to you. No personal life stories or situations will be recorded. All the information collected will be confidential; it will not bear your name or of the provider and will be kept under lock and key.

**Withdrawal or refusal to participate**

Participation in this study is voluntary and you should feel free to refuse to participate at all or to stop participating at any stage. If you refuse observation or terminate observation your consultation will proceed as normal. If you refuse there will be no effect on the current services you receive at this facility or any other facility. Do you have any questions?

No Yes (if yes, note the questions)

_________________________________________________________________________________________

If you have any other questions please ask for:

Annie Mwangi at Population Council Nairobi Tel: +254 020 2713480 (Kenya)

If you have any questions about participating in the research please contact:

KEMRI 020 2722541 for Kenya

Would you be willing to be in the overall study: Yes No (If NO, Thank provider and end interview)

Will you allow me to do this observation? No Yes

**Subject’s Statement**

I have read or have been read the above considerations regarding my participation. I have been given a chance to ask any questions I may have and my questions have been answered to my satisfaction. I understand that the information I give will be kept private. I understand that I may withdraw from this study at any time. My withdrawal from this study or my refusal to participate will in no way affect me or my family’s medical care from this centre or any other centre. I agree to participate in this study as a volunteer.

_______________________________________________ ________________ Thank the client.

#### *Signature of Respondent Date*

Signature of witness/interviewer: I/we have explained to the volunteer in a language she or he understands the procedures to be followed in this study, and the risks and benefits involved.

_______________________________________________ _________________

Signature of Interviewer Date

-------------------------------------------------- ------------------------------------

Signature of witness Date

# Appendix 2b: Consent from Client for short interview following CPI

**Title of RESEARCH project: Assessing the benefits and costs of integrating HIV into reproductive health /postnatal services in Kenya and Swaziland**

Good morning/afternoon.

My name is________________. I am working with Population Council an international non profit organisation, the London School of Hygiene and Tropical Medicine (LSHTM), UK, International Planned Parenthood Federation (an international non profit organisation) and Ministry of Health to understand the issues around the integration of HIV and RH services. We would also like to learn how family planning and HIV/AIDS prevention and care services are provided to women who come for RH and HIV services. The Principle Investigators are Charlotte Warren from Population Council, Susie Mayhew and Charlotte Watts from LSHTM.

**Purpose of study:** The purpose of asking these questions is to learn more about the health situation and needs of women after they have received postnatal care and other services in this area. The Ministry of Health will use the results obtained from the study to improve the information and services provided to women in this community. If you agree to be interviewed, you will be asked questions about your knowledge of HIV/RH problems and issues, sources of information and services for women in this community, and your attitude towards provision of reproductive health information and services. If you refuse to be interviewed there will be no effect on the current services you receive.

**Benefits:** The results from this project are expected to contribute towards better HIV/RH services for the community. The information you give us will be used to provide recommendations to the Kenya Ministry of Health, as well as to health care providers and communities in other countries.

**Discomfort and risks:** Some of the questions we will be asking might be considered too personal. It is possible that a question you will be asked may be embarrassing or make you feel uncomfortable. You are free ask me to stop discussions if you are uncomfortable, or decline to answer any single question if it upsets or makes you uncomfortable. If so, you may choose not to answer it. If you agree to be interviewed, you will not be charged for your participation. Your participation in this study is completely voluntary and nothing you say will be communicated to anyone else. It is strictly confidential. If you do not understand certain questions, then ask me to explain them to you.

**Confidentiality:** All information that you provide will be considered private and confidential and will be used only for purposes of this study. Your name will not appear on the interview form. You will be given a number that will be used in place of your name on the interview form to help us to contact you for the second interview. Only the researchers from the Population Council or LSHTM involved in the study will see the interview form, and only the Study Coordinator will have access to the list that has your name and your number. Any written or verbal report using data collected from this study will not use your name or any other information that may identify you. All completed forms will be locked in a room at the Population Council’s office in Nairobi and only the Study Coordinator will have access to the forms.

As part of the monitoring program, someone may ask to talk with you again to check if I have completed this informed consent form. They may ask questions about the risk, benefit, and procedures in this study. If an interview is requested, you have the option of accepting the interview or declining the interview. Again, all information will be kept confidential

You can stop the interview at any time. If any problem arises, and you wish to withdraw from the study, or if you have any questions, you may contact:

Annie Mwangi at Population Council Nairobi Tel: +254 020 2713480 (Kenya)

If you have any questions about participating in the research please contact KEMRI 020 2722541 for Kenya

Would you be willing to be in the overall study: Yes No (If NO, Thank participant and end interview)

You now have an opportunity to ask me questions concerning the study and your consent to participate. Do you have any questions? (If yes, note the questions) Yes No

_____________________________________________________________________________________

Do you agree to participate in this study? (Circle one) Yes No

Thank you,

I have read or have heard and/or understood the above considerations regarding my participation. I have been given a chance to ask any questions I may have and my questions have been answered to my satisfaction. I understand that the information I give will be kept private. I understand that I may withdraw from this study at any time. My withdrawal from this study or my refusal to participate will in no way affect me or my family’s medical care from this centre or any other centre. I agree to participate in this study as a volunteer.

-------------------------------------------------- ------------------------------------

Signature of interviewee Date

OR IF RESPONDENT CANNOT READ OR WRITE:

Signature of witness/interviewer in case informant cannot write and date (certifying that informed consent has been given verbally by informant)

Interviewers’ declaration:

I ...................................................................... hereby declare that I have explained clearly to the informant, the aims, objectives and benefits of this study and I read this statement to the respondent, that she fully understood its meaning, and that she verbally agreed to participate in the study

-------------------------------------------------- ------------------------------------

Signature of interviewer Date

-------------------------------------------------- ------------------------------------

Signature of witness Date

# Appendix 3A: Consent from Client for cohort study

**Title of RESEARCH project: Assessing the benefits and costs of integrating HIV into reproductive health /postnatal services in Kenya and Swaziland**

Good morning/afternoon.

My name is_______________. I am working on with Population Council an international non profit organisation, the London School of Hygiene and Tropical Medicine (LSHTM), UK, International Planned Parenthood Federation (an international non profit organisation) and Ministry of Health to understand the issues around the integration of HIV and RH services. We would also like to learn how family planning and HIV/AIDS prevention and care are provided to women who come for FP and HIV services. The Principle Investigators are Charlotte Warren from Population Council, Susie Mayhew and Charlotte Watts (LSHTM).

**Purpose of study**

The purpose of asking these questions is to learn more about the health situation and needs of women after they have received FP/HIV and other services in this area. The Ministry of Health will use the results obtained from the study to improve the information and services provided to women in this community.

**Procedure:** If you agree to be interviewed, you will be asked questions about your knowledge of FP/HIV problems and issues, sources of information and services for women in this community, and your attitude towards provision of reproductive health information and services. In addition to talking with you today, we would like to ask you to agree for a person to meet and talk with you in about six months time, and again in 18 months and 30 months time (a period of two and a half years). The purpose of these additional interviews is to find out how you have been using the services available for yourself, and your views on how these services could be improved. With your agreement, I would like to write down your name, physical address, and phone number, so that we will be able to contact you for the next interviews. We will call or contact (through letter) you to make an appointment, and then you will be invited to choose whether a person comes to your house or whether you meet the person at a place that you choose. If you would rather meet the person away from your home, we will reimburse the costs of your transport to that place. The form with your name on will be kept under lock and key and will be separate from the questionnaires so that nobody will know what you said. When we report what we have found in these interviews, no one will know that you participated

**Benefits**: The results from this project are expected to contribute towards better /HIV/RH services for the community. The information you give us will be used to provide recommendations to the Kenya Ministry of Health, as well as to health care providers and communities in other countries.

**Discomfort and risks:** We believe that this study is safe and do not expect you to suffer any harm or injury because of your participation in it. However some of the questions we will be asking might be considered too personal. It is possible that a question you will be asked may be embarrassing or make you feel uncomfortable. You are free ask me to stop discussions if you are uncomfortable, or decline to answer any single question if it upsets or makes you uncomfortable. If so, you may choose not to answer it. If you agree to be interviewed, you will not be paid for your participation. Your participation in this study is completely voluntary and nothing you say will be communicated to anyone else. It is strictly confidential. If you do not understand certain questions, then ask me to explain them to you. If at any time over the two and half years you decide you do not wish to continue being interviewed you have the option of declining further interviews.

**Compensation:** For subsequent interviews, if you wish to meet away from home, we will compensate you for your transport arrangements and a refreshment for you to attend the interviews.

**Confidentiality:** All information that you provide will be considered private and confidential and will be used only for purposes of this study. Your name will not appear on the interview form. You will be given a number that will be used in place of your name on the interview form to help us to contact you for the second interview. Only the researchers from the Population Council involved in the study will see the interview form, and only the research team coordinator will have access to the list that has your name and your number. Any written or verbal report using data collected from this study will not use your name or any other information that may identify you. All completed forms will be locked in a room at the Population Council’s office in Nairobi and only the research team coordinator will have access to the forms. If you do not wish to be interviewed there will be no effect on the current health services you receive.

As part of the monitoring program, someone may ask to talk with you again to check if I have completed this informed consent form. They may ask questions about the risk, benefit, and procedures in this study. If an interview is requested, you have the option of accepting the interview or declining the interview. Again, all information will be kept confidential. You can stop the interview at any time. If any problem arises, and you wish to withdraw from the study, or if you have any questions, you may contact:

Annie Mwangi who can be contacted at Population Council Nairobi Tel: +254 020 2713480 (Kenya)

If you have any other questions about participating in the research please contact:

KEMRI 020 2722541 for Kenya

Provider’s signature Date

You now have an opportunity to ask me questions concerning the study and your consent to participate. Do you have any questions? (If yes, note the questions) Yes No

_____________________________________________________________________________________

Do you agree to participate in this study? (Circle one) Yes No

Thank you,

I have read or have heard and/or understand the above considerations regarding my participation. I have been given a chance to ask any questions I may have and my questions have been answered to my satisfaction. I understand that the information I give will be kept private. I understand that I may withdraw from this study at any time. My withdrawal from this study or my refusal to participate will in no way affect my or my family’s medical care from this centre or any other centre. I agree to participate in this study as a volunteer.

-------------------------------------------------- ------------------------------------

Signature of interviewee Date

OR IF RESPONDENT CANNOT READ OR WRITE:

Signature of witness/interviewer in case informant cannot write and date (certifying that informed consent has been given verbally by informant)

Interviewers’ declaration:

I ...................................................................... hereby declare that I have explained clearly to the informant, the aims, objectives and benefits of this study and I read this statement to the respondent, that she fully understood its meaning, and that she verbally agreed to participate in the study

-------------------------------------------------- ------------------------------------

Signature of interviewer Date

-------------------------------------------------- ------------------------------------

Signature of witness Date

# Appendix 3B: Client tracing form for Cohort study

**Title of RESEARCH project: Assessing the benefits and costs of integrating HIV into FP services in Kenya and Swaziland**

Ask for name, address and contact information of each woman who agrees to be contacted in 6 months, 18 months and 30 months time for subsequent interviews as per the informed consent.

Date of interview……… Client’s Code No: Facility code No: (where recruited)

1. Names of the client (3)…………………………………………………………………..
2. Telephone No……………………………………………………………………………
3. Client’s Social Name e. g Mama Jane or the name that they are known …………….
4. Husband’s Name………………………………………………………………………..
5. Husband’s Social Name or name they are known for where they live…………………
6. Family Name……………………………………………………………………………
7. Alternative telephone numbers (optional)

Husbands Tel: ……………………………………..

Neighbor’s Tel……………………………………...

Friend’s Tel…………………………………………

1. Postal address (if available)

…………………………………………………….

……………………………………………………..

………………………………………………………

1. Physical /Residence address (Get clear details on how to get client at her home) …………………………………………………………………………………………………………………………………………………………………………………………………………………………………………………………………………………………………………………………………………………………………………………………………………
2. Client’s place of work (Physical) if any………………………………………………
3. Nearest Landmark (describe if possible)…………………………………………….
4. Name of prominent person near client’s residence………………………………….
5. Division………………………………………………………………………………...
6. Location……………………………………………………………………………….
7. Sub location…………………………..Name of Assistant Chief……………………
8. Village…………………………………Name of village elder……………………….

Draw a map from the Nearest Landmark to client’s home (overleaf)

# Appendix 3C Informed consent during home visit

**Title of RESEARCH project: Assessing the benefits and costs of integrating HIV into reproductive health /postnatal services in Kenya and Swaziland**

Good morning/afternoon. My name is________________. I am working on with Population Council an international nonprofit organisation, the London School of Hygiene and Tropical Medicine (LSHTM), UK, International Planned Parenthood Federation (an international nonprofit organisation) and Ministry of Health/Ministry of Health to understand the issues around the integration of HIV and RH services. We would also like to learn how family planning and HIV/AIDS prevention and care services are provided to women who come for these services. The Principle Investigators are Charlotte Warren from Population Council, Susie Mayhew and Charlotte Watts from LSHTM

I am part of the same team of individuals who talked with you 6 / 18/30 months ago at the postnatal clinic/FP clinic at the facility. At that time you agreed to be contacted for other interviews. We are interviewing you to hear about your experiences with the care and information you have received. I would like to sit down with you and ask you some questions. This will take approximately 20-30 minutes. I will be writing on this form. It does not have your name on it. We will call or contact (through letter) you again to make an appointment, and then you will be invited to choose whether a person comes to your house or whether you meet the person at a place that you choose. If you would rather meet the person away from your home, we will reimburse the costs of your transport to that place.

**Benefits**: The benefit is that you will contribute to the good of your community. What you say is important and valuable, and will help the health authorities to plan better services for your area.

**Risks and discomfort**: We believe that this study is safe and do not expect you to suffer any harm or injury because of your participation in it. However some of the questions may be of a personal nature and may make you feel uncomfortable. Participation in this study is voluntary. You may refuse to answer any question, and you may choose to stop the interview at any time. For the next interview (after one year) you will be invited to choose whether a person comes to your house or whether you meet the person at a place that you choose if you would rather meet the person away from your home, we will reimburse the costs of your transport to that place.

If you do not wish to continue to be interviewed there will be no effect on the current health services you, or your family, receive.

**Confidentiality:** All information that you provide will be considered private and confidential and will be used only for purposes of this study. Your name will not appear on the interview form. You will be given a number that will be used in place of your name on the interview form to help us to contact you for any subsequent interviews. Only the researchers from the Population Council involved in the study will see the interview form, and only the research team coordinator will have access to the list that has your name and your number. Any written or verbal report using data collected from this study will not use your name or any other information that may identify you. All completed forms will be locked in a room at the Population Council’s office in Nairobi and only the research team coordinator will have access to the forms.

I will keep the information confidential. Your name will not appear on the questionnaire. This form will be kept under lock and key. At the end of the study, the results of all the answers will be compiled together.

**Compensation**: For subsequent interviews, if you wish to meet away from home, we will compensate you for your transport arrangements and a refreshment for you to attend the interviews.

Do you have any questions? Yes ____ No ____

If yes, note the questions

---------------------------------------------------------------------------------------------------------------------

If you have further questions after the interview please ask for:

Annie Mwangi at Population Council Nairobi Tel: +254 020 2713480 (Kenya)

If you have any other questions about participating in the research please contact:

KEMRI 020 2722541 for Kenya

Will you allow me to do this interview? Yes No (if no thank participant and finish)

As part of the monitoring program, someone may ask to talk with you again to check if I have completed this informed consent. They may ask questions about the risk, benefit, and procedures in this study. If an interview is requested, you have the option of accepting the interview or declining the interview. Again, all information will be kept confidential.

**Subject’s Statement**

I have read or have been read the above considerations regarding my participation. I have been given a chance to ask any questions I may have and my questions have been answered to my satisfaction. I understand that the information I give will be kept private. I understand that I may withdraw from this study at any time. My withdrawal from this study or my refusal to participate will in no way affect my or my family’s medical care from this centre or any other centre. I agree to participate in this study as a volunteer. I agree to allow someone to contact me again to request another interview.

_______________________________________________ ________________

#### *Signature of Respondent Date*

I, the interviewer, have explained to the volunteer in a language she or he understands the procedures to be followed in this study, and the risks and benefits involved.

_______________________________________________ _________________

Signature of Interviewer Date

-------------------------------------------------- ------------------------------------

Signature of witness Date

# Appendix 4A: Community Survey: Household Roster Form

**Title of RESEARCH project: Assessing the benefits and costs of integrating HIV into reproductive health /postnatal services in Kenya and Swaziland**

This form is for an initial screening of household members. It may be asked of the head of household if present or any responsible adult. Ages do not need verification or probing, they may be approximate; they are necessary for screening purposes only.

**Deliver Preliminary Introduction:**

Good morning/afternoon. My name is________________. I am working on with Population Council an international nonprofit organisation, the London School of Hygiene and Tropical Medicine (LSHTM), UK, International Planned Parenthood Federation (an international nonprofit organisation) and Ministry of Health to understand the issues around the integration of HIV and RH services. We are visiting households to ask women some questions about using certain health services. Is it alright if I ask about the number and ages of people living here today and then possibly ask if I can talk in more depth to one of them? The Principle Investigators are Charlotte Warren from Population Council, Susie Mayhew and Charlotte Watts from LSHTM

My name is ___________. I’m working on behalf of the MOH/MOHSW and LSHTM.

**CLUSTER NUMBER: HOUSEHOLD NUMBER:**

**INTERVIEWER’S CODE:**  **DATE (dd-mm-yy):** **-****-**

Firstly please tell me the age and gender of the person you consider to be head of this household and then list all the people who live in this household with their gender and age.

Names are not going to be recorded.

| **Household resident** | **Sex** | **Age in years*** |
| --- | --- | --- |
| Head of household |  |  |
|  |  |  |
|  |  |  |
|  |  |  |
|  |  |  |
|  |  |  |
|  |  |  |
|  |  |  |
|  |  |  |
|  |  |  |
|  |  |  |
|  |  |  |
|  |  |  |
| * those less than 1 year old record “under 1” | | |

Thank the respondent.

Consider household member for delivery of main questionnaire.

# Appendix 4B: Introductions for use in community surveys

**Title of RESEARCH project: Assessing the benefits and costs of integrating HIV into reproductive health /postnatal services in Kenya and Swaziland**

**Preliminary introduction**

Good morning/afternoon. My name is________________. I am working on with Population Council an international non profit organisation, the London School of Hygiene and Tropical Medicine (LSHTM), UK, International Planned Parenthood Federation (an international non profit organisation) and Ministry of Health to understand the issues around the integration of HIV and RH services. We are visiting households to ask women some questions about using certain health services. Is it alright if I can talk in more depth to you? The Principle Investigators are Charlotte Warren from Population Council, Susie Mayhew and Charlotte Watts from LSHTM.

**Purpose of study** The purpose of asking these questions is to learn more about the available health services and HIV/RH needs of women and men in this area. The Ministry of Health will use the results obtained from the study to improve the information and services provided to women in this community. If agreeable the Household Roster is then commenced. If men and women aged over 15 years are present in household select using selection grid.

If adolescents aged 15 – 17 years, ensure parental consent is obtained first.

**More detailed introduction to men and woman selected:** My name is ___________. I’m working on behalf of the MOH and LSHTM. We are visiting households to ask women and men some questions about using certain health services, mostly to do with women’s health, family planning and services related to HIV infection. The survey is being held so that we can see what services women and men use and how we might improve them.

**Procedure:** Even if you do not use these services we would like to get some opinions from you.

Participation in this study is voluntary. You may refuse to answer any question, and you may choose to stop the interview at any time. Refusing to participate will not affect your or your family’s access to services at any health facility.

**Benefits**: The benefit is that you will contribute to the good of your community. What you say is important and valuable, and will help the health authorities to plan better services for your area.

**Risks and discomfort**: We believe that this study is safe and do not expect you to suffer any harm or injury because of your participation in it. Some of the questions are about private matters but we do not record your name or address on the forms and the information is completely anonymous and confidential. Because of the confidential nature of some questions it would be good if we could sit somewhere private. This form will be kept under lock and key. At the end of the study, the results of all the answers will be compiled together. You will not receive money or reward of any kind if you agree to be interviewed.

Do you have any questions? Yes ____ No ____

If yes, note the questions -------------------------------------------------------------------------------------------------------------

If you have further questions please ask for:

Annie Mwangi at Population Council Nairobi Tel: +254 020 2713480 (Kenya)

If you have any questions about participating in the research please contact KEMRI 020 2722541 for Kenya

Would you be willing to be in the overall study and allow me to interview?

Yes No (If NO, Thank participant and end the interview)

------------------------------------------ ---------------------------------------------

Participants signature Date

As part of the monitoring program, someone may ask to talk with you again to check if I have completed this informed consent. They may ask questions about the risk, benefit, and procedures in this study. If an interview is requested, you have the option of accepting the interview or declining the interview. Again, all information will be kept confidential.

**Subject’s Statement:** I have read or have been read the above considerations regarding my participation. I have been given a chance to ask any questions and my questions have been answered to my satisfaction. I understand that the information I give will be kept private. I understand that I may withdraw from this study at any time. My withdrawal from this study or my refusal to participate will in no way affect my or my family’s medical care from this centre or any other centre. I agree to participate in this study as a volunteer.

_________________________________________ ________________

#### *Signature of Respondent Date*

I, the interviewer, have explained to the volunteer in a language she or he understands the procedures to be followed in this study, and the risks and benefits involved.

_______________________________________________ _________________

Signature of Interviewer Date

-------------------------------------------------- ------------------------------------

Signature of witness Date

# Appendix 4C: Parent/guardian Information sheet - community survey

**Title of RESEARCH project: Assessing the benefits and costs of integrating HIV into reproductive health /postnatal services in Kenya and Swaziland**

**(For parents of participants 15 - 17 years)**

We are inviting your child to take part in a research project. This is a study to help us understand the advantages or disadvantages to offering integrated sexual / reproductive health (SRH) and HIV services, in particular youth services. Before you decide whether to allow your child to take part, you need to understand why the research is being done and what it would involve for your child. Please take time to read or to listen as I read the following information carefully. Talk to others about the study if you wish. Ask us if there is anything that is not clear or if you would like more information.

**1. What is the research about?**

There are many reasons why it is good to integrate sexual / reproductive health (SRH) and HIV services. This means clinics where family planning and sexually transmitted infections, HIV testing and HIV treatment are all offered together in one clinic. Most HIV infections are sexually transmitted or associated with pregnancy, childbirth and breastfeeding. Many of the behaviours that prevent HIV transmission also prevent sexually transmitted infections and some prevent unintended pregnancies. People who use SRH services often benefit from HIV/AIDS services and vice versa. However there are still a lot of unanswered questions about the best way to offer these services to patients, in particular youth services. This study will gather evidence to determine the costs and benefits of a range of models for delivering integrated HIV and SRH services for youth in order to reduce HIV (and associated stigma) and unintended pregnancies. This research is a joint initiative by the International Planned Parenthood Association (IPPF) (funded by the Gates Foundation), the Population Council an international nonprofit organisation, and the London School of Hygiene and Tropical Medicine (LSHTM), (London). The Principle Investigators are Charlotte Warren from Population Council, Susie Mayhew and Charlotte Watts from LSHTM

**2. Why has my child been invited to take part?**

Your child has been invited to take part because they may have used these types of services, they may know someone who has used these types of services or may wish to use these services in the future.

**3. What will happen to my child if I allow them to take part?**

If you give permission for your child to take part in the study (and they also agree to take part), we will ask you to give us your written permission. Your child will either be asked to take part in a focus group discussion with other youth or a confidential tape-recorded interview with a researcher (so that they do not have to write everything down). This will probably last around half an hour to an hour and can happen at a time and place that is convenient to them. The researcher will ask them about theirs or their friends’ experience of using these types of services and to seek their advice about how these services can be improved for youth.

**4. Are there any possible benefits or risks in taking part?**

Although there is no immediate benefit to your child in taking part, the information that they provide will help us to understand the advantages or disadvantages to offering integrated sexual / reproductive health (SRH) and HIV services for youth. We believe that this study is safe and do not expect your child to suffer any harm or injury because of their participation in it.

**5. What if there is a problem?**

Any complaint about the way your child has been dealt with during the study or any possible harm they might suffer will be addressed. If you or your child has a concern about any aspect of this study, you should ask to speak to the researchers who will do their best to answer your questions.

**6. Will my child’s taking part in the study be kept confidential?**

The information that is collected during the interview will not contain anything that could identify your child to anyone outside the research team. They will be given a special research number that will be used for storing the information instead of using their name. Only members of the research team will have access to the interviews. Tape recordings and transcripts will be kept in a locked filing cabinet for no longer than 10 years after the end of the research project. No one will be told that your child has been interviewed for the study.

**7. Does my child have to take part?**

No, your child does not have to take part in the study – it is up to you and your child to decide. We will describe the study, explain what is involved and go through this information sheet, which we will then give to you to keep. If you give permission for your child to take part but then change your mind, you are free to withdraw your child from the study at any time, without giving a reason.

**8. What will happen if I do not want my child to carry on with the study?**

A decision not to allow your child to participate or to withdraw them from the study will not affect you or your child in any way, including your access to health care, either now or in the future. If the interview touches on areas that your child finds upsetting, they will be free to stop the interview at any time and withdraw from the study. If they wish to meet with a professional counsellor to discuss any issues raised by the study, this will be arranged for them.

**9. What will happen to the results of the research study?**

The results of the study will be discussed at medical conferences and published in scientific journals.

**10. Who has reviewed the study?**

This study has been reviewed and given favourable opinion by the Ethics Research Committee, Kenya Medical Research Institute/ SEC, MOHSW Swaziland.

**11. Further information.** Further details about the study can be obtained from the researcher or [X] at FLAS.

If you have further questions after the interview please ask for:

Annie Mwangi at Population Council Nairobi Tel: +254 020 2713480 (Kenya)

If you have any questions about participating in the research please contact KEMRI 020 2722541 for Kenya

Thank you for taking the time to read about this research study.

# APPENDIX 4D: Parent/guardian consent form

Title of Project: **Assessing the benefits and costs of integrating sexual/reproductive health and HIV services for youth**

Centre Number___________ Participant Identification Number:__________

**Note: If participant is under 18, parent or guardian must sign.**

Name of interviewer: _________________________________

Please initial each box to show that you agree:

1. I confirm that both myself and my child have read and understand the information sheet

for the above study. We have had the opportunity to consider the information, ask questions

and have had these answered satisfactorily.

1. I understand that this participation is entirely voluntary and there will be no negative

consequences if my child does not participate. I can withdraw my consent [or my child

can withdraw consent] at any time.

1. I confirm that myself and my child agree to the interview being taped and give consent for anonymous verbatim quotations to be used in publication.

4. In the unlikely event that myself or my child are no longer available to communicate

with the research team, I understand that any personal data collected will continue to be

used confidentially for the purposes of this study.

5. I hereby give my permission for my child to attend and participate in the above-named study.

______________________________ ________________ ___________________

Name of Parent/Guardian Date Signature

______________________________ ________________ ___________________

Researcher Date Signature

When completed, 1 copy for parent/guardian; 1 for researcher site file.

# Appendix 5A: Participant Information Sheet (FGD) Community Based

**Title of RESEARCH project: Assessing the benefits and costs of integrating HIV into reproductive health /postnatal services in Kenya and Swaziland**

We are inviting you to take part in a research project. This is a study to help us understand the advantages or disadvantages to offering integrated sexual / reproductive health (SRH) and HIV services. Before you decide whether to take part, you need to understand why the research is being done and what it would involve for you. Please take time to read / allow me to read to you the following information carefully. Talk to others about the study if you wish. Ask us if there is anything that is not clear or if you would like more information.

**1. What is the research about?**

There are many reasons why it is good to integrate sexual / reproductive health (SRH) and HIV services. This means facilities where family planning, sexually transmitted infections, HIV testing and HIV treatment are all offered together in one facility. Most HIV infections are sexually transmitted or associated with pregnancy, childbirth and breastfeeding. Many of the behaviours that prevent HIV transmission also prevent sexually transmitted infections and some prevent unintended pregnancies. People who use SRH services often benefit from HIV/AIDS services and vice versa. However there are still a lot of unanswered questions about the best way to offer these services to patients. This study will gather evidence to determine the costs and benefits of a range of models for delivering integrated HIV and SRH services in order to reduce HIV (and associated stigma) and unintended pregnancies. This research is a joint initiative by the International Planned Parenthood Association (IPPF) (funded by the Gates Foundation), the Population Council/CSO and the London School of Hygiene and Tropical Medicine (LSHTM), (London). The Principle Investigators are Charlotte Warren from Population Council, Susie Mayhew and Charlotte Watts from LSHTM.

**2. Why have I been invited to take part?**

You have been invited to take part because you are not currently using these types of services and we would like to find out what you know about them.

**3. What will happen to me if I decide to take part?**

If you decide to take part we will ask you to give us your written permission to show you have agreed to join the study. You will be asked to take part in a confidential tape-recorded focus group discussion with a researcher and about 5 other people who are also not currently using these services. The discussion will be tape-recorded so that the researcher does not have to write everything down. This will probably last around an hour. The researcher will ask you about your knowledge about these types of services in your area, any previous experiences using these types of services and to seek your advice about how these services can be improved.

**4. Are there any possible benefits in taking part?**

Although there is no immediate benefit to you in taking part, the information that you provide will help us to understand the advantages or disadvantages to offering integrated sexual / reproductive health (SRH) and HIV services in different settings.

**5. Are there any possible risks in taking part?**

We believe that this study is safe and do not expect you to suffer any harm or injury because of your participation in it. All participants must agree that information will not be shared to preserve confidentiality.

**6. What if there is a problem?**

Any complaint about the way you have been dealt with during the study or any possible harm you might suffer will be addressed. If you have a concern about any aspect of this study, you should ask to speak to the researchers who will do their best to answer your questions.

**7. Will my taking part in the study be kept confidential?**

The information that is collected during the focus group will not contain anything that could identify you to anyone outside the research team. Any potential revelations of personal information from fellow participants must be kept confidential and not discussed outside the group. This will be agreed with the whole group before we start the discussion. You will be given a special research number that will be used for storing the information instead of using your name. Only members of the research team will have access to the interviews. Tape recordings and transcripts will be kept in a locked filing cabinet for no longer than 10 years after the end of the research project. No one will be told that you have been interviewed for the study.

**7. Do I have to take part?**

No, you do not have to take part in the study – it is up to you to decide. We will describe the study, explain what is involved and go through this information sheet, which we will then give to you to keep. If you do decide to take part but then change your mind you are free to withdraw at any time, without giving a reason. A decision not to participate or to withdraw from the study will not affect you in any way, including your access to health care or current services you receive either now or in the future.

**8. What will happen if I don’t want to carry on with the study?**

Even if you decide to take part, you are still free to withdraw at any time without giving a reason. If the interview touches on areas that you find upsetting, you are free to stop the interview at any time and withdraw from the study. If you wish to meet with a professional counsellor to discuss any issues raised by the study, this will be arranged for you.

**9. What will happen to the results of the research study?**

The results of the study will be discussed at medical conferences and published in scientific journals. However I assure you that your name or any other personal information will not appear in any published scientific journal.

**10. Who has reviewed the study?**

This study has been reviewed and given favourable opinion by the Ethics Research Committee, Kenya Medical Research Institute/ Scientific Ethics Committee MOHSW, Swaziland.

**11. Further information**. Further details about the study can be obtained from:

Annie Mwangi at Population Council Nairobi Tel: +254 020 2713480 (Kenya)

If you have any questions about participating in the research please contact KEMRI 020 2722541 for Kenya

**Thank you for taking the time to read about this research study.**

# Appendix 5B: Participant consent form

**Title of RESEARCH project: Assessing the benefits and costs of integrating HIV into reproductive health /postnatal services in Kenya and Swaziland**

Centre Number: ___________

Participant Identification Number: __________

Name of interviewer: _________________________________

Please initial each box to show that you agree:

1. I confirm that I have read / I have heard and/or understand the information sheet for the above study. I have had the opportunity to consider the information, ask questions and have had these answered satisfactorily.
2. I understand that my participation is voluntary and that I am free to withdraw at any time

without giving any reason.

1. I agree to the interview being taped and give consent for anonymous verbatim quotations

to be used in publication.

1. In the unlikely event that I will lose contact with the research team, I understand that any personal data collected will continue to be used confidentially for the purposes of this study.
2. I agree to take part in the above study.

______________________________ ________________ ___________________

Name of participant Date Signature

______________________________ ________________ ___________________

Researcher Date Signature

When completed, 1 for provider; 1 for researcher site file.

# Appendix 5C: Participant Information Sheet (Provider)

**Title of RESEARCH project: Assessing the benefits and costs of integrating HIV into reproductive health /postnatal services in Kenya and Swaziland**

We are inviting you to take part in a research project. This is a study to help us understand the advantages or disadvantages to offering integrated sexual / reproductive health (SRH) and HIV services. Before you decide whether to take part, you need to understand why the research is being done and what it would involve for you. Please take time to read the following information carefully. Talk to others about the study if you wish. Ask us if there is anything that is not clear or if you would like more information.

**1. What is the research about?**

There are many well-established reasons that support the rationale for linking sexual / reproductive health (SRH) and HIV services closely. Most HIV infections are sexually transmitted. Many of the behaviours that prevent HIV transmission also prevent STIs and some prevent unintended pregnancies. Individuals who use SRH services often benefit from HIV/AIDS services and vice versa. There remain many unanswered questions about the effectiveness of different models of integration. This study will gather evidence to determine the costs and benefits of a range of models for delivering integrated HIV and SRH services in high and medium HIV prevalence settings in order to reduce HIV (and associated stigma) and unintended pregnancies. This research is a joint initiative by the International Planned Parenthood Association (IPPF) (funded by the Gates Foundation), the Population Council /CSO and the London School of Hygiene and Tropical Medicine (LSHTM), (London). The Principle Investigators are Charlotte Warren from Population Council, Susie Mayhew and Charlotte Watts from LSHTM.

**2. Why have I been invited to take part?**

You have been invited to take part because you are a provider of these services.

**3. What will happen to me if I decide to take part?**

If you decide to take part we will ask you to give us your written permission to show you have agreed to join the study. You will be asked to take part in a confidential tape-recorded interview with a researcher (so that they do not have to write everything down). This will probably last around half an hour to an hour. The researcher will ask you about your experience of being a provider of these types of services and to seek your advice about how these services can be improved. The interview can happen in a place that is convenient to you.

**4. Are there any possible benefits in taking part?**

Although there is no immediate benefit to you in taking part, the information that you provide will help us to understand the advantages or disadvantages to offering integrated sexual / reproductive health (SRH) and HIV services in different settings.

**5. Are there any possible risks in taking part?**

We believe that this study is safe and do not expect you to suffer any harm or injury because of your participation in it.

**6. What if there is a problem?**

Any complaint about the way you have been dealt with during the study or any possible harm you might suffer will be addressed. If you have a concern about any aspect of this study, you should ask to speak to the researchers who will do their best to answer your questions.

**7. Will my taking part in the study be kept confidential?**

The information that is collected during the interview will not contain anything that could identify you to anyone outside the research team. You will be given a special research number that will be used for storing the information instead of using your name. Only members of the research team will have access to the interviews. Tape recordings and transcripts will be kept in a locked filing cabinet for no longer than 10 years after the end of the research project. No one will be told that you have been interviewed for the study.

**8. Do I have to take part?**

No, you do not have to take part in the study – it is up to you to decide. We will describe the study, explain what is involved and go through this information sheet, which we will then give to you to keep. If you do decide to take part but then change your mind you are free to withdraw at any time, without giving a reason. A decision not to participate or to withdraw from the study will not affect you in any way, including your current employment, access to health care, either now or in the future.

**9. What will happen if I don’t want to carry on with the study?**

Even if you decide to take part, you are still free to withdraw at any time without giving a reason. If the interview touches on areas that you find upsetting, you are free to stop the interview at any time and withdraw from the study. If you wish to meet with a professional counsellor to discuss any issues raised by the study, this will be arranged for you.

**10. What will happen to the results of the research study?**

The results of the study will be discussed at medical conferences and published in scientific journals. However I assure you that your name or any other personal information will not appear in any published scientific journal.

**11. Who has reviewed the study?**

This study has been reviewed and given favourable opinion by the Ethics Research Committee, Kenya Medical Research Institute

**12. Further information**.

If you have further questions after the interview please contact:

Annie Mwangi at Population Council Nairobi Tel: +254 020 2713480 (Kenya)

If you have any questions about participating in the research please contact:

KEMRI 020 2722541 for Kenya

# Appendix 5D: PROVIDER CONSENT FORM

**Title of RESEARCH project: Assessing the benefits and costs of integrating HIV into reproductive health /postnatal services in Kenya and Swaziland**

Centre Number:___________

Participant Identification Number:__________

Name of interviewer: _________________________________

Please initial each box to show that you agree:

1. I confirm that I have read and understand the information sheet for the above study. I have had the opportunity to consider the information, ask questions and have had these answered satisfactorily.
2. I understand that my participation is voluntary and that I am free to withdraw at any time

without giving any reason.

1. I agree to the interview being taped and give consent for anonymous verbatim quotations

to be used in publication.

1. In the unlikely event that I am no longer available (e.g transfer to another region) for communication with the research team, I understand that any personal data collected will continue to be used confidentially for the purposes of this study.
2. I agree to take part in the above study.

______________________________ ________________ ___________________

Name of participant Date Signature

______________________________ ________________ ___________________

Researcher Date Signature

When completed, 1 for provider; 1 for researcher site file.

# Appendix 6: Key Research Personnel

*Charlotte Warren, RH Associate in the Population Council’s Nairobi office* ***Principle Investigator***

She implements a range of reproductive health operations research projects in several countries of east and southern Africa, including Kenya and Swaziland. Charlotte currently manages the Council’s research projects on integrating VCT into FP services in Kenya and developing integrated postpartum services for HIV+ and HIV- mothers and their babies in Swaziland. A manager and technical specialist in Reproductive Health with almost twenty years experience in Africa, Warren has demonstrated experience in policy development and planning with ministry of health and partners, and in understanding the realities of strengthening RH and HIV/AIDS service delivery models in Kenya and Swaziland. She has extensive experience in managing collaborative programmes with Ministries of Health, academic institutions and international NGOs, with in-depth experience in project cycle management including design and appraisal, implementation as well as M&E. Warren is a member of the WHO Technical Working Group on standards for neonatal care and the Council’s Africa representative for the Healthy Newborn Partnership.

*Ian Askew, Director, Reproductive Health Services and Research*, *RH Program Population Council*

Dr. Askew has more than two decades of experience in reproductive health operations research, and for the past 17 years has lived and worked throughout Africa. Askew’s professional interests include integrating reproductive health and HIV and AIDS services; developing and improving the quality of community-based reproductive health services; understanding the causes and consequences of sexual and gender-based violence, including abandonment of female genital mutilation/cutting. Before joining the Population Council, Askew was Deputy Director at the Institute of Population Studies at the University of Exeter in England. He has published over 150 articles and given presentations at conferences and workshops around the world.

*Annie Mwangi, Program Officer RH program, Population Council, Nairobi*

Annie Mwangi has over 20 years experience in health service delivery and management in Kenya. Her recent experience includes coordinating operations research projects and quality of service improvement activities in RH in Kenya specifically on integrated PNC and HIV services. In addition Annie has extensive experience in curriculum development and developing and implementing standards for maternal care using criterion-based audit and is a skilled trainer and facilitator in RH and PMTCT. She served as senior nursing officer for the Obstetrics and Gynaecological Unit in Kenyatta Hospital for 12 years and as the Chairperson of the Midwives Chapter of the National Nurses Association of Kenya. Annie is a long-term provider of technical assistance and advice to the DRH, MOH Kenya

*Dr Susannah Mayhew, MA, PhD, Senior Lecturer, RH and health policy, LSHTM* ***Principle Investigator***

Dr Mayhew directs the LSHTM component of the DFID-funded Reproductive Health and Rights Research Programme Consortia and has managed a number of other multi-country projects on reproductive health issues primarily in sub-Saharan Africa. She has researched and published widely on the operational and systems barriers and opportunities for integrated sexual and reproductive health service delivery in Africa since the 1990s. She has advised WHO in expert consultation meetings on Integration and was invited to give a paper at the recent Addis conference on integration (though could not attend). She has over ten years experience conducting qualitative and quantitative research on reproductive and sexual health policies and programmes in Africa and Asia. Current projects include assessing different models of delivering HIV-related services to high-risk groups in Pakistan and investigating issues of marketing, delivery and use of emergency contraception in four sub-Saharan African countries.

*Andy Sloggett Senior Lecturer on demography, statistics and analysis, LSHTM* ***Acting PI***

Mr Sloggett’s main interests are in demographic analysis of mortality and fertility and other health-related processes.  His strengths are analytical skills in demography and their interface with statistics and epidemiology. He has extensive developing-country experience, including survey analysis and experience of large and complex datasets.  He has particular experience of cancer survival analysis.  He also teaches demography, statistics and analysis techniques to postgraduate students and organises two Masters courses at the London School of Hygiene and Tropical Medicine.

*Professor Charlotte Watts, Department of Public Health and Policy, LSHTM* ***Principle investigator***

Professor Watts has been conducting public health research on HIV for fifteen years, and heads the HIV Tools Research Group, a multi-disciplinary team of economists, mathematicians and behavioural scientists that conduct modelling and economic research to address applied questions related to HIV programming. The group has substantial expertise in conducting cost analyses of different models of HIV prevention and treatment, and linking this to modelling analyses to estimate the impact and cost-effectiveness of HIV interventions in different settings. Work to date includes studies on the potential importance of different new HIV prevention technologies, including microbicides, analyses of the provision of voluntary counseling and HIV testing; and the economic impact of HIV morbidity in rural Thailand. The group is providing economic and modelling support to the ongoing DFID funded Microbicides Development Programme phase III trial, and to the Gates funded Avahan Project in Southern India. Professor Watts has a Ph.D in mathematics, and further epidemiological and public health training. She has extensive fieldwork experience in sub-Saharan Africa and Asia, long-term research relationships with WHO, UNAIDS, and the Global Campaign for Microbicides, and is an advisor to several international HIV projects.

[*Dr Lilani Kumaranayake*](mailto:lilani.kumaranayake@lshtm.ac.uk) *Ph.D. Senior Lecturer in Health Economics ,LSHTM*

An economist specializing in HIV/AIDS, the economics of health systems in lower-resources settings and econometric modelling for health policy analysis. She has worked in more than 15 countries in Sub-Saharan Africa, Eastern Europe, and Asia, including Tanzania, Zimbabwe, South Africa, Cameroon, Ghana, Burkina-Faso, Malawi and Mozambique. Dr. Kumaranayake is an expert in costing and cost-effectiveness and authored the UNAIDS Costing Guidelines for HIV Prevention Strategies. She has more than 150 publications including peer-review journals, books, book chapters, and policy reports. She is currently undertaking research related to economic evaluation, development of new methodologies for assessing costs and resource allocation, the economics of ART, and HIV/AIDS and health systems. She has been a Technical Advisor to international organizations such as the WHO, UNFPA, UNAIDS and the Wellcome Trust. She is currently a member of the UNAIDS Reference Group on Economics, the Organising Committee of the International AIDS Economics Network, and UK Medical Research Council College of Experts.

*Joanna Busza, M. Sc., Lecturer in Sexual & Reproductive Health, LSHTM*

Ms. Busza has over ten years postgraduate experience in research, design and implementation of programmes in sexual & reproductive health, HIV/AIDS prevention and treatment, monitoring and evaluation, participatory action, training and technical assistance to NGO/CBOs. She has done significant work with community-based programmes and vulnerable populations, particularly sex workers and young people, and has recent field experience in Thailand, Cambodia, Tanzania and South Africa. Prior to working at LSHTM, she served as Programme Officer for the Population Council and was responsible for developing operations research studies to evaluate innovative approaches for reaching marginalised communities with sexual and reproductive health services.

[*Fern Terris-Prestholt*](http://www.lshtm.ac.uk/php/personal/hpu/fern_terris-prestholt.htm)*, M.Sc. Lecturer in Economics; economist, LSHTM*

An economist with eight years experience in the economics of HIV prevention. She has undertaken cost analyses of a variety of HIV prevention and care projects in Uganda, Tanzania, Zambia and South Africa. Projects include the ProTEST initiative, which integrated HIV and TB services, strengthened STD treatment, condom promotion, VCT and projects targeted to youth and sex-workers. Fern has more recently been focusing on the economics of microbicides and barrier methods, particularly relating to potential demand and costs. She continues her work on costing of integrated and/or new services into existing ones, such as rapid syphilis test into services for hard to reach populations in the Amazon in Brazil and drug users in China.

*Natalie Friend-du Preez PhD, Research Fellow, LSHTM*

Research has focused on the use of health services and health-seeking behaviour in southern Africa: e.g. health-seeking behaviour for childhood illnesses in South Africa; the use of traditional, complementary and alternative medicines amongst people living with HIV in South Africa (and impact on adherence); symptoms and quality of life amongst HIV patients in South Africa; marital status and its links with HIV transmission in Zimbabwe. Studies also include the non-clinical problems that HIV patients bring to the clinical setting in east London (three-quarters of whom are first-generation Africans).

# Appendix 7: Research Ethics Certificates for Population Council staff

# Appendix 8: Final drafts of Study instruments:

1. CLIENT – PROVIDER INTERACTION CT/FP consultations
2. cLIENT EXIT INTERVIEW
3. FACILITY INVENTORY
4. PROVIDER INTERVIEW (KNOWLEDGE BASED)
5. cLIENT COHOrT INTERVIEW
6. Community Survey for WOMen
7. Community Survey for Men
8. Client in-depth interview guide
9. FP/RH Client in-depth interview guide
10. FP/RH Provider in-depth interview guide
11. HIV Provider in-depth interview guide
12. COSTING TOOL

# Appendix 8.1: CPI

| **Assessing the Benefits of integrated HIV and Reproductive Health Services in Kenya**  **CLIENT-PROVIDER INTERACTION**  **PRE-INTERVENTION**  **Central Province** |
| --- |

**Questionnaire Number**

FACILITY Name ………………… District ………………………….

**FACILITY CATEGORY**

(1**:** Hospital 2: Health Centre, 3: Dispensary, 4: IPPF Clinic)

**Date of assessment**: Day/Month/Year:

**Name of Data Collector** …………………………………………………………... Code:

**Time Started:** (Use 24 hours clock)

**Supervisor’s review**: Name ___________________ Signature _________________Date__________

Designation of observed provider

***(Circle the appropriate code)***

Enrolled Nurse/Midwife (Certificate) ………………………………………………………1

Registered Nurse/Midwife (Diploma) ……………………………………………………...2

Degree Nurses (Degree e.g. BScN) …………………………………………………………3

Clinical Officers (Diploma) ………………………………………………………………...4

Medical Officers (Degree) ………………………………………………………………….5

Other specify ………………………………………………………......................................6

| **INSTRUCTIONS TO OBSERVER**  **Obtain permission from the provider and consent from the client before observing the consultation. When observing, be as discreet as possible and on no account become involved in the interaction. Make sure that the provider knows that you are not there to evaluate her/him and that you are not an “expert” who can be consulted during the session. Try to sit in a position such that you are behind the patient but not directly in view of the provider. Make notes as quickly as possible. For each of the items, circle the answer that most appropriately reflects your assessment of what happened during the interaction. Use the appropriate section of the observation based on the reason for consultation.** |
| --- |

| **A. GREETING AND ASSESSING CLIENT** | | | |
| --- | --- | --- | --- |
| 1 | **Does the provider greet the client in a friendly/ respectful manner?**  ***(Circle the appropriate code)*** | No 0  Yes 1  Did not greet 2 | |
| 2 | **What was the main purpose of the visit as initially indicated by the client?** | New user | 1 |
|  |  | Repeat/refill client | 2 |
|  |  | Switching | 3 |
|  |  | Gap in FP use | 4 |

| **3** | **Are the following areas discussed/mentioned during the consultation (observe and circle if mentioned):** | **Circle each one mentioned** |
| --- | --- | --- |
|  | Client’s age | Y |
|  | Marital status | Y |
|  | Ever been pregnant | Y |
|  | Number of pregnancies | Y |
|  | Number of children alive | Y |
|  | Desired number of children | Y |
|  | Age of youngest child | Y |
|  | Currently breastfeeding | Y |
|  | Timing of next birth | Y |
|  | Date of last menses | Y |
|  | Intercourse since last menses | Y |
|  | Previous use of FP | Y |
|  | Discussed family planning with spouse/partner | Y |
|  | Partner cooperation | Y |
|  | HIV serostatus | Y |
|  | History of medical conditions hypertension, anaemia, cardiac disease, malignancies, etc. | Y |

| B. CLIENT COUNSELING | | |
| --- | --- | --- |
| **4** | **Which information, education, and communication (IEC) materials does the provider use during the consultation?** | **Circle each one mentioned** |
|  | BCS job aids (algorithm, counseling FP method cards, brochures/pamphlets) | Y |
|  | General brochures/leaflets | Y |
|  | Contraceptive samples (pills, condom, etc.) | Y |
|  | Posters | Y |
|  | Anatomical models (e.g. Dildo) | Y |
|  | Other counseling tools | Y |
|  | Other _____________________________________(specify) | Y |
| **5** | Does the provider take the clients blood pressure | No 0  Yes 1 |
| **6** | **WHICH METHODS ARE DISCUSSED DURING THE CONSULTATION**  **(Circle each one mentioned)** | |
|  | Combined pill (ovral, triphasil, nordett) ***(Chaguo langu)*** | Y |
|  | Progestin only pill (microval) | Y |
|  | IUCD | Y |
|  | Male condom | Y |
|  | Female condom | Y |
|  | Injectables (depo or nuristerate) | Y |
|  | Sterilization (btl/vasectomy) | Y |
|  | Emergency contraception | Y |
|  | Implants (norplant,jadelle, implanon) | Y |
|  | LAM | Y |
|  | Natural Family Planning methods (Standard Days Method etc) | Y |
|  | Other _________________________________ (specify) | Y |
| **7** | **Does the provider promote or emphasize one method in particular?**  ***[if a repeat user skip to question 8]*** | No 0  Yes 1 |
| **8** | (If yes) which method does the provider emphasize?  **(Circle each one mentioned)** | |
|  | Combined pill (ovral, triphasil, nordett) ***(Chaguo langu)*** | Y |
|  | Progestin only pill (microval) | Y |
|  | IUCD | Y |
|  | Male condom | Y |
|  | Female condom | Y |
|  | Injectables (depo or noristerate) | Y |
|  | Sterilization (btl/vasectomy) | Y |
|  | Emergency contraception | Y |
|  | Implants (Norplant, Jadelle, Implanon) | Y |
|  | LAM | Y |
|  | Natural Family Planning methods (Standard Days Method etc) | Y |
|  | Other _________________________________ (specify) | Y |
| **9** | Does the client mention a preferred method? | No 0  Yes 1 |
| **10** | If yes) which method does the client prefer?  **(Circle each one mentioned)** | |
|  | Combined pill (ovral, triphasil, nordett) ***(Chaguo langu)*** |  |
|  | Progestin only pill (microval) |  |
|  | IUCD |  |
|  | Male condom |  |
|  | Female condom |  |
|  | Injectables (depo or noristerate) |  |
|  | Sterilization (btl/vasectomy) |  |
|  | Emergency contraception |  |
|  | Implants (Norplant, Jadelle, Implanon) |  |
|  | LAM |  |
|  | Natural Family Planning methods (Standard Days Method etc) |  |
|  | Other _________________________________ (specify) |  |
| **11** | Does the client receive her preferred method | No 0  Yes 1 |
| **12** | Which method does she actually receive  **(Circle each one mentioned)** | |
|  | Combined pill (ovral, triphasil, nordett) ***(Chaguo langu)*** | Y |
|  | Progestin only pill (microval) | Y |
|  | IUCD | Y |
|  | Male condom | Y |
|  | Female condom | Y |
|  | Injectables (depo or noristerate) | Y |
|  | Sterilization (btl/vasectomy) | Y |
|  | Emergency contraception | Y |
|  | Implants (Norplant, Jadelle, Implanon) | Y |
|  | LAM | Y |
|  | Natural Family Planning methods (Standard Days Method etc) | Y |
|  | Other _________________________________ (specify) | Y |
| **13** | **For the method the client receives does the provider:**  **Circle each one mentioned** | |
|  | Explain how method works | Y |
|  | Explain advantages/benefits | Y |
|  | Explain disadvantages | Y |
|  | Explain how to use method | Y |
|  | Discuss practices affecting effectiveness | Y |
|  | Discuss possible side effects | Y |
|  | Discuss management of side effects | Y |
|  | Discuss return to clinic if she has complications | Y |
|  | Discuss possibility of changing method | Y |
|  | Discuss emergency contraception | Y |
|  | Give oral or written follow-up instructions | Y |
|  | Advise client when to return for re-supply | Y |
|  | Discuss emergency contraceptive incase a client forgets to take her contraceptive or use a condom to prevent pregnancy | Y |
| **14** | **If client does not receive any FP method, WHY NOT?** |  |
|  | Not appropriate method/contraindications | Y |
|  | Method not available | Y |
|  | Told to return during/after menses | Y |
|  | Changed mind after listening to provider | Y |
|  | Suspect pregnancy | Y |
|  | Other (specify) ___________________________________________ | Y |

| **STI Risk assessment and condoms** | | | |
| --- | --- | --- | --- |
| Does the provider do any of the following? | | | **Circle each one mentioned** |
|  | Does the provider discuss STI with the client? | | Y |
|  | Does the provider discuss HIV/AIDS with the client? | | Y |
|  | Does the provider discuss STI and/or HIV risk factors with the client? | | Y |
|  | If yes, what risk factors does the provider discuss? | | Y |
|  | Multiple partners | | Y |
|  | STIs | | Y |
|  | Unprotected sexual intercourse | | Y |
|  | Not knowing partner’s status | | Y |
|  | Partner has multiple partners | | Y |
|  | Other (specify) | | Y |
| Does the provider give any of the following? | | | **Circle each one mentioned** |
|  | Give information on symptoms of an STI? | | Y |
|  | Advise to seek medical treatment if they notice any symptoms of an STI? | | Y |
|  | Advise that an STI may be asymptomatic? | | Y |
|  | Advise that an STI can increase transmission of HIV? | | Y |
|  | Screen for STI | | Y |
|  | Provide syndromic management of STIs | | Y |
|  | Refer the client elsewhere for STI services Write in facility or unit if within same facility | | Y |
| **Does the provider discuss the following on condoms:** | | | **Circle each one mentioned** |
|  | Mention condoms? | | Y |
|  | Ask if client ever used condom? | | Y |
|  | Ask if client used condom at last sex | | Y |
|  | Does the provider mention explicitly that condoms protect against STI &/or HIV? | | Y |
|  | Does the provider mention explicitly that condoms protect against pregnancy? | | Y |
|  | Does the provider encourage the use of condoms for STI/HIV prevention along with the use of another method? | | Y |
|  | Does the provider emphasize correct and consistent use of a condom? | | Y |
|  | Does the provider discuss how to negotiate use of condom with partner? | | Y |
|  | Does the provider give information on how to use a male condom? | | Y |
|  | Does the provider give information on how to use a female condom? | | Y |
|  | Does the provider discuss other STI/HIV prevention methods other than the condom? | | Y |
|  | Does the provider mention EC as a backup for condom breakage? | | Y |
|  | Does the provider give the client any condoms? | | Y |
|  | *Write number of male condoms* | | Y |
|  | *Write number of female condoms* | | Y |
|  | Does the provider advise client where she can get more condoms | | Y |
| **Does the provider discuss other STI/HIV prevention methods other than the condom? Which?** | | |  |
|  | Abstinence | | Y |
|  | Monogamy | | Y |
|  | Partner monogamy | | Y |
|  | Knowing your partner’s status | | Y |
|  | Knowing your own status | | Y |
| **HIV Counseling and Testing** | | | |
| **22** | Does the provider do any of the following? | | **Circle each one mentioned** |
|  | Ask for history of signs and symptoms of RTIs/STIs | | Y |
|  | Ask number of sexual partners | | Y |
|  | Ask Partner’s number of sexual partners | | Y |
|  | Does the provider mention Counseling and Testing (CT) for HIV? | | Y |
|  | Does the provider ask the client if she has already tested? | | Y |
|  | Does the provider ask when she last tested for HIV? | | Y |
|  | Does the provider discuss what the test can tell the client? | | Y |
|  | Does the provider explain about the window period? | | Y |
|  | Does the provider give the client information on where to get VCT? | | Y |
|  | Does the provider offer the client counseling and testing for HIV? | | Y |
| 23 | Asks if the client accepts to be tested for HIV? | | No 0  Yes 1 |
| 24 | Is the client counseled and tested for HIV in this session? | | Y |
| 25 | If tested, how long is spent on counseling? PRE TEST………………. *Write in minutes POST TEST………………..*  How long is spent on the testing procedure? *TESTING…*……………… | | |
| 26 | Does the provider refer the client for CT? | | No 0  Yes 1 |
| 27 | If yes: | | Circle each one mentioned |
|  | Which facility or unit? (Write in)……………………………… | | Y |
|  | Does the provider give the client a CT referral letter | | Y |
|  | Does the client raise specific issues regarding the referral letter? | | Y |
|  | If yes, which ones (specify) | |  |
| **FOR HIV POSITIVE CLIENTS ONLY (if diagnosed in consultation or disclosed to provider)** | | | |
| **28** | **Does the provider discuss the following:** | | **Circle each one mentioned** |
|  | Ask if client is on ART? | | Y |
|  | Mention drug interactions between hormonal methods and ART? | | Y |
|  | Discuss positive living for people living with HIV? | | Y |
|  | Ask about the client’s general state of health? | | Y |
|  | Mention the need to prevent unintended pregnancies among women who are HIV-infected? | | Y |
| **ALL CLIENTS other issues** | | | |
| **29** | | **What other health issues are mentioned / discussed with the client during the consultation?** | **Circle each one mentioned** |
|  | | Gynecological exam | Y |
|  | | Pap smear | Y |
|  | | Cervical screening using VIA | Y |
|  | | Pregnancy test | Y |
|  | | PMTCT | Y |
|  | | Gender based violence/abuse | Y |
|  | | Breast examination | Y |
|  | | General health and well-being | Y |
|  | | Childhood vaccinations | Y |
|  | | Child growth monitoring | Y |
|  | | ART | Y |
|  | | Opportunistic infections in HIV positive clients | Y |
|  | | Other ____________________________________________ (specify) | Y |
| **30** | | **Does the provider do any of the following:** | **Circle each one mentioned** |
|  | | Gynecological exam | Y |
|  | | Pap smear | Y |
|  | | Cervical screening using VIA | Y |
|  | | Pregnancy test | Y |
|  | | Breast examination | Y |
|  | | Childhood vaccinations | Y |
|  | | Child growth monitoring | Y |
|  | | Other ________________________________________ (specify) | Y |
| **31** | | **Which services does the provider refer the client for:** | **Circle each one mentioned** |
|  | | Gynecological complications | Y |
|  | | Pap smear | Y |
|  | | Cervical screening using VIA | Y |
|  | | Pregnancy test | Y |
|  | | PMTCT | Y |
|  | | Breast examination/mammogram | Y |
|  | | STI services | Y |
|  | | ART clinic | Y |
|  | | TB clinic | Y |
|  | | Opportunistic infections in HIV positive clients | Y |
|  | | Support group | Y |
|  | | Other ___________________________________________ (specify) | Y |
| **32** | | **Does the health worker give the client a reminder, in writing, of when to return?** | No 0  Yes 1 |
| **33** | | **Does the provider** | Y |
|  | | Use clients name when talking to her/him | Y |
|  | | Ask if client understood the information | Y |
|  | | Encourage client to ask questions | Y |
|  | | Use client record | Y |
|  | | See client in privacy where no one could hear the conversation | Y |
|  | | Ensures confidentiality | Y |
|  | | Looks at clients health card during consultation | Y |
|  | | Document data in the register | Y |
|  | | Give the client a return date | Y |
|  | | Record the return date on client’s card | Y |

| 34 | **Any other comments/impressions (write overleaf if necessary)** |
| --- | --- |

**Time of observation ended (in 24 hour clock)**

**Thank client, if she is willing to be interviewed then give her code for exit interview**

# Appendix 8. 2: Client exit DRAFT

| **Assessing the Benefits of integrated HIV and Reproductive Health Services in Kenya**  **CLIENT EXIT INTERVIEW**  **PRE-INTERVENTION (SEPTEMBER 2008)**  **Central Province** |
| --- |

**Questionnaire Number**

FACILITY name District ……………….

**FACILITY CATEGORY**

(1 Hospital 2. Health centre 3. Dispensary 4. IPPF clinic)

**Date of assessment**: Day/Month/Year:

**Name of Data Collector** …………………………………………………………... Code:

**Time Started:** (Use 24 hours clock)

**Supervisor’s review**: Name ___________________ Signature

_________________Date__________

| ***I am going to ask you a few short questions. Thank you again for taking the time to help us with our study today. Please remember that this information will be kept strictly confidential and your name will not be recorded anywhere on this form.*** | | | | | | | | | | | | |
| --- | --- | --- | --- | --- | --- | --- | --- | --- | --- | --- | --- | --- |
| **Section 1 background socio-demographic information** | | | | | | | | | | |  |  |
|  | | Sex of client | | 1. Male 2. Female | | | | |  | |  |  |
|  | | How old are you? (age in complete in years) | |  | | | | |  | |  |  |
|  | | What is your current marital status? | | 1. Married monogamous 2. Married polygamous 3. Living together 4. Single 5. Divorced/separated/widowed 6. Others | | | | |  | |  |  |
|  | | What is the highest level of school that you have attained? | | 1. None 2. Primary 1 to 4 3. Primary 5 to 8 4. Secondary 5. College/tertiary | | | | |  | |  |  |
|  | | What is your current employment? | | 1. Professional/ technical/ managerial 2. Clerical 3. Sales and services 4. Skilled manual 5. Unskilled manual 6. Domestic service 7. Agriculture 8. Unemployed 9. Other, specify........................................ | | | | |  | |  |  |
|  | | Was this your first visit to this clinic? | | 1. Yes 2. No | | | | |  | |  |  |
|  | | How many times have you been before | | times | | | | |  | |  |  |
|  | | How long did it take you to get to the clinic today? | | hours  minutes | | | | |  | |  |  |
|  | | Where do you live? *(write name). Need to be specific, location, division , rural/urban* | | Location…………………………  Division………………………….  Rural/Urban……………………... | | | | |  | |  |  |
|  | | How long did you have to wait today to be seen by the nurse or doctor? *(first consultation only if queued more than once)* | | hours  minutes | | | | |  | |  |  |
| **Section 2 FP history** | | | | | | | | | | |  |  |
|  | | What was the main (first) reason that you came here today?  (**New**:- new to clinic, new to FP following gap in use of FP,) | | 1. Family planning (new client) 2. Family planning (resupply of method) 3. FP client switching method 4. FP method after childbirth 5. Other (specify): _______________________ | | | | |  | |  |  |
|  | | How many different nurses or doctors did you actually see today? | | ----------------------- insert number | | | | |  | |  |  |
|  | | What did you see them for? *(insert type of service )* | | 1st …………………………..  2^nd^…………………………..  3^rd^…………………………..  4^th^…………………………..  5^th^ ………………………….. | | | | | | |  |  |
|  | | Did you attend any group health talks at the facility today? | | 1. Yes 2. No | | | | | | Skip to Q15 |  |  |
|  | | What was the subject of the group talk?  *Circle all that apply* | | 1. Family planning 2. HIV education 3. HIV testing counseling 4. Other (specify):.. | | | | | |  |  |  |
|  | | Please could you tell me; have you ever been pregnant? | | 1. Yes 2. No | | | | | | Skip Q22 |  |  |
|  | | How many times have you been pregnant? | |  | | | | | |  |  |  |
|  | | How many living children do you have? | |  | | | | | | If none, skip to Q22 |  |  |
|  | | How old is your youngest child? | | Months  Years | | | | | |  |  |  |
|  | | Were you using a contraceptive method when you became pregnant with your last child? | | 1. Yes 2. No | | | | | | Skip to Q22 |  |  |
|  | | If yes, which method were you using?    **(Circle only one method)** | | 1. Progestin only pills 2. Combined oral contraceptive pill 3. Emergency contraception 4. Injectable 5. Male condom 6. Female condom 7. Hormonal implant 8. IUD 9. Female sterilization (BTL) 10. Vasectomy 11. Lactational Amenorrhea Method 12. Natural Family Planning Method 13. Other (specify) ………………… | | | | | |  |  |  |
|  | | Would you like to have (a/another) child, or would you prefer not to have any (more) children? | | 1. Have a/another child 2. No more children 3. Undecided /don’t know | | | | | | Skip to Q24  Skip to Q24 |  |  |
|  | | How long would you like to wait from now before the birth of (a/another) child?  (if does not know probe) | | 1. [ ] [ ] months 2. [ ] [ ] years 3. Cannot get pregnant 4. After marriage 5. Other, specify …………………. | | | | | |  |  |  |
|  | | What is your desired family size? | | [ ] [ ] number | | | | | |  |  |  |
|  | | Is the same number as your husband /partner? | | 1. Wants same number of children as partner 2. Partner wants more children than client 3. Partner wants less children than client 4. Partner does not want any children 5. Don’t know | | | | | |  |  |  |
|  | | Could you please tell me who referred you here for family planning services | | 1. Health worker 2. Self referral 3. Family member 4. Friend 5. Other, specify………………….. | | | | | |  |  |  |
|  | | Were you already using a method of contraception when you arrived here today? | | 1. Yes 2. No | | | | | | Skip to Q30 |  |  |
|  | | Which method(s) did you use last?  (prior to the consultation today) | | 1. Progestin only pills 2. Combined oral contraceptive pill 3. Emergency contraception 4. Injectable 5. Male condom 6. Female condom 7. Hormonal implant 8. IUD 9. Female sterilization (BTL) 10. Vasectomy 11. Lactational Amenorrhea Method 12. Natural Family Planning Method 13. Other, specify............................. | | | | | |  |  |  |
|  | | Are (were) you using a condom with this method | | 1. Yes 2. No | | | | | |  |  |  |
|  | | Please can you confirm which FP methods you were given today?  **(circle all that are mentioned)**  *(See chart for brand names of different pills)* | | 1. Progestin only pills 2. Combined oral contraceptive pill 3. Emergency contraception 4. Injectable 5. Male condom 6. Female condom 7. Hormonal implant 8. IUD 9. Female sterilization (BTL) 10. Vasectomy 11. Lactational Amenorrhea Method 12. Natural Family Planning Method 13. Other specify...............................   ………………………………… | | | | | |  |  |  |
|  | | Is this your preferred method? | | 1. Yes 2. No | | | | | | Skip to Q33 |  |  |
|  | | Why do you prefer this method? | | Write in.................................................... | | | | | |  |  |  |
|  | | Why didn’t you get your preferred method | | Write in....................................................  …………………………………………. | | | | | |  |  |  |
|  | | Did you change methods today? | | 1. Yes 2. No | | | | | |  |  |  |
| **SECTION 3: SEXUAL BEHAVIOUR (reassure the client that some of the questions may be sensitive but confidentiality will be maintained)** | | | | | | | | | | |  |  |
|  | | How many times have you had sex in the last month? | | | 1. [ ] number 2. Never had sex | | | | Skip Q38 | |  |  |
|  | | The last time you had sexual intercourse, was a condom used? | | | 1. Yes 2. No | | | | Skip Q38 | |  |  |
|  | | What was the main reason you used a condom on that occasion? | | | 1. Wanted to prevent STI/HIV 2. Wanted to prevent pregnancy 3. Wanted to prevent both STI/HIV and pregnancy 4. Did not trust partner/feels partner has other partners 5. Partner insisted 6. Don’t know 7. Other (specify) | | | |  | |  |  |
|  | | Do you think your partner has other partners? | | | 1. Yes 2. No 3. Don’t know | | | |  | |  |  |
|  | | Other than this partner, have you had sex with anyone else in the last 12 months? | | | 1. Yes 2. No | | | |  | |  |  |
|  | | In the last 12 months, how many people overall have you had sex with? | | | NUMBER | | | |  | |  |  |
| **SECTION 4: KNOWLEDGE OF SEXUALLY TRANSMITTED INFECTIONS AND HIV** | | | | | | | | | | |  |  |
|  | | As far as you know, are there any diseases that can be transmitted through sexual intercourse? | | | 1. Yes 2. No 3. Don’t know | | | | |  |  |  |
|  | | As far as you know, what are the ways you can become infected with HIV/AIDS?  **(Circle all that are mentioned)** | | | 1. Unprotected sexual intercourse 2. From mother to child during pregnancy 3. From mother to child during birth 4. Breastfeeding 5. Blood transfusion 6. Contact with infected blood 7. Others (specify): | | | | |  |  |  |
|  | | Do you know of ways of protecting yourself from STI/HIV and AIDS?  **(CIRCLE ALL that are mentioned)** | | | 1. Use a condom 2. Stick to one partner 3. Abstain from sex 4. Male circumcision 5. Thigh sex 6. Others (specify) | | | | |  |  |  |
|  | | During the consultation did the provider discuss whether you have ever had an HIV test? | | | 1. Yes 2. No | | | | |  |  |  |
|  | | Please can you tell me, have you ever been tested for HIV? | | | 1. Yes 2. No | | | | | Skip to Q54 |  |  |
|  | | How many times have you tested for HIV? | | | Write in number | | | | |  |  |  |
|  | | Now I would like you to tell me where you received the (last) HIV test?  (if tested more than once ask about the most recent test) | | | 1. ANC clinic 2. PNC clinic 3. VCT clinic 4. FP clinic 5. Postnatal ward 6. Other specify……………… 7. Dont know/can’t remember | | | | |  |  |  |
|  | | Would you feel comfortable returning and speaking to the health provider who gave you your results if you have any questions about HIV? | | | 1. Yes 2. No 3. Don’t know | | | | |  |  |  |
|  | | Please can you tell me:  What was the date of your last test? *(write today’s date if applicable)* | | | Write approximate date  Month  Year | | | | |  |  |  |
|  | | Did you share your results with anyone? | | | 1. Yes 2. No | | | | | Skip to Q52 |  |  |
|  | | Please can you tell me who was the first person you shared your HIV test results with? | | | 1. Partner 2. Mother 3. Father 4. Female relative 5. Male relative 6. Friend 7. Other health provider | | | | |  |  |  |
|  | | If you did not share your results with anyone – please can you tell me why? | | | Write in.................................. | | | | |  |  |  |
|  | | Would you be prepared to tell me your HIV status?  *Please know that I will keep this information confidential* | | | 1. HIV Negative 2. HIV Positive 3. HIV test done but not received results 4. Does not want to disclose results | | | | |  |  |  |
|  | | Has your partner been tested for HIV? | | | 1. Yes 2. No 3. Don’t know | | | | |  |  |  |
|  | | Has he shared his results with you? | | | 1. Yes 2. No | | | | |  |  |  |
| **Section 5 FOR HIV positive clients only** | | | | | | | | | | |  |  |
|  | | **For HIV positive clients only:**  When you received your results did the provider refer you to the ART clinic for follow-up? | | | 1. Yes 2. No 3. Don’t know | | | | |  |  |  |
|  | | **For HIV positive clients only:**  If yes which ART clinic were you referred to? | | | 1. Within the same facility 2. Another facility 3. Other specify | | | | |  |  |  |
|  | | **For HIV positive clients only:**  Have you been to the ART clinic yet? | | | 1. Yes 2. No | | | | |  |  |  |
|  | | **For HIV positive clients only:**  Have you ever had your CD4 count done? | | | 1. Yes 2. No 3. Don’t know | | | | | Skip to Q61  Skip |  |  |
|  | | **For HIV positive clients only:**  Do you know what your CD4 count is? | | | 1. Yes , write in count [ ] 2. CD 4 count done but does not know level | | | | |  |  |  |
|  | | **For HIV positive clients only:**  Are you on any treatment? | | | 1. Drugs for opportunistic infection 2. Pre –HAART Prophylaxis 3. HAART 4. TB treatment 5. Not on any treatment 6. Other specify | | | | |  |  |  |
|  | | **For HIV positive clients only:**  Are you enrolled with any support groups | | | 1. Yes 2. No | | | | |  |  |  |
|  | | **For HIV positive clients only**  If yes which ones? | | | Write in -------------------------------- | | | | |  |  |  |
|  | | **For HIV positive clients only:**  If you have children, has any of your children/child been tested for HIV? | | | 1. All tested 2. Some tested (if applicable) 3. None tested 4. Do not have children | | | | | Skip to Q67  Skip to Q67 |  |  |
|  | | **For HIV positive clients only:**  Are any of your children HIV positive? | | | 1. All are HIV positive 2. Some are HIV positive 3. None are HIV positive 4. Don’t know | | | | | Skip to Q67  Skip to Q67 |  |  |
|  | | **For HIV positive clients only:**  Are your children/Is your child receiving care in this clinic? | | | 1. None are 2. All are 3. Some are 4. Don’t know | | | | |  |  |  |
| **Section 6: Review of services and Satisfaction with services**  *Now I would like to finish by asking you some questions on how you feel about the services here. I would like to remind you that NONE of the information in this questionnaire will be told to the nurses and doctors here.*  **I am going to read some statements about your visit to the clinic today. I would like you to say how much you agree with these statements - please say agree, disagree, or undecided/mixed opinion.** | | | | | | | | | | |  |  |
|  | | **Statements** | | | | **Agree** | **Disagree** | **Mixed** | | |  |  |
|  | | I was greeted warmly today | | | | 1 | 2 | 3 | | |  |  |
|  | | Staff were friendly | | | | 1 | 2 | 3 | | |  |  |
|  | | The nurses were easy to understand | | | | 1 | 2 | 3 | | |  |  |
|  | | The nurse listened to me | | | | 1 | 2 | 3 | | |  |  |
|  | | Staff were helpful in providing information | | | | 1 | 2 | 3 | | |  |  |
|  | | I felt free to ask questions | | | | 1 | 2 | 3 | | |  |  |
|  | | I was provided all the information I wanted during today’s consultation | | | | 1 | 2 | 3 | | |  |  |
|  | | My consultation was private | | | | 1 | 2 | 3 | | |  |  |
|  | | The nurse ensured me about confidentiality | | | | 1 | 2 | 3 | | |  |  |
|  | | The waiting time was reasonable | | | | 1 | 2 | 3 | | |  |  |
|  | | The staff treat me with respect | | | | 1 | 2 | 3 | | |  |  |
|  | | I would like to come back to this clinic again | | | | 1 | 2 | 3 | | |  |  |
|  | | I would recommend this clinic to a friend | | | | 1 | 2 | 3 | | |  |  |
|  | | In your own opinion, which is the best clinic in this town or this region to receive family planning? | | | Write in name of clinic................ | | |  | | |  |  |
|  | | Why?  *Circle all that apply* | | | 1. Proximity/distance to clinic 2. Friendly staff 3. Privacy 4. Confidentiality 5. Integrated so can disguise reason for visit 6. No shortages of drugs/supplies 7. Waiting times 8. Opening hours 9. Other (specify): | | |  | | |  |  |
|  | | Where is the best place to go to get an HIV test? | | | *Write name of clinic* | | |  | | |  |  |
|  | | Why? | | | 1. Proximity/distance to clinic 2. Friendly staff 3. Privacy 4. Confidentiality 5. Integrated so can disguise reason for visit 6. No shortages of drugs/supplies 7. Waiting times 8. Opening hours 9. Able to see doctor 10. Other (specify) | | |  | | |  |  |
|  | | Do you feel that all your health care needs were met today? | | | 1. Yes 2. No 3. Other (specify): | | | Skip to Q87 | | |  |  |
|  | | Which other health issues would you have liked to discuss or get help with today? | | | 1. None 2. Postpartum care 3. Ante natal care 4. HIV testing 5. HIV care/treatment 6. STI services 7. Pregnancy test 8. Child immunisation 9. Follow up visit 10. Information and counseling 11. Infant feeding advice 12. More on family planning 13. Other (specify) …………………   …………………………………. | | |  | | |  |  |
|  | | Why did you not discuss them with the provider?  Write in------------------------------------------------------------------------------ | | | | | |  | | |  |  |
|  | | How much did you pay for your consultation(s) today? | | |  | | |  | | |  |  |

**Time of observation ended (in 24 hour clock)**

***Thank the client for taking the time to do the interview***

# Appendix8.3: facility inventory

| DRAFT  **Assessing the Benefits of integrated HIV and Reproductive Health Services in Kenya**  **FACILITY inventory**  **PRE-INTERVENTION**  **Central Province** |
| --- |

**Questionnaire Number**

FACILITY NAME…………………………….. District ………………………..

**FACILITY CATEGORY**

1. Hospital
2. Health centre
3. Dispensary
4. Private clinic

**Date of assessment**: Day/Month/Year:

**Name of Data Collector** …………………………………………………………... Code:

**Time Started:** (Use 24 hours clock)

**Supervisor’s review**:

Name ___________________ Signature _________________Date__________

| **INSTRUCTIONS TO DATA COLLECTOR: This assessment should be completed by observing the facilities that are available and through discussions with the person in charge of MCH and /or ART unit on the day of the visit. IN ALL CASES, you should verify that items exist by actually observing them yourself. If you are not able to observe then code accordingly. Remember that the objective is to identify the equipment, supplies and facilities that currently exist and not to evaluate the performance of the staff or clinic. For each item, circle the response or describe as appropriate**. |
| --- |

| **SECTION A: POPULATION** | | | | | |
| --- | --- | --- | --- | --- | --- |
| **Q101: What is the catchment population of this facility?**  **(population served by the facility)?** | | | | | |
| **SECTION B: STAFFING** | | | | | |
| **Q201: ASK: Please can you give an overview of the personnel in your health facility. Although we mainly focus on MCH and ART clinic information about the whole facility can give a more complete impression on staffing levels** | | **ASK How many are assigned to work in MCH & ART unit (read the list)** | | |  |
|  |  | **Total in the Facility** | **No. in MCH/FP Unit** | **No. in ART clinic** |  |
| 1. Number of specialist doctors |  |  |  |  |  |
| 1. Number of medical officers |  |  |  |  |  |
| 1. Number of clinical officers |  |  |  |  |  |
| 1. Number of registered nurse midwives |  |  |  |  |  |
| 1. Number of enrolled nurse midwives |  |  |  |  |  |
| 1. Number of laboratory technologists and/or technicians | |  |  |  |  |
| 1. Number of pharmacists and/or pharmacist technicians | |  |  |  |  |
| 1. Number of associated medical staff (radiographers/radiologists, physiotherapists etc) | |  |  |  |  |
| 1. Number of lay counselors | |  |  |  |  |
| 1. Number of administrators |  |  |  |  |  |

| **Q202: Has any of the staff in MCH/FP received in-service training in any of the following?** | | | **Yes** |
| --- | --- | --- | --- |
| 1. PMTCT | | | Y |
| 1. HIV counseling and testing | | | Y |
| 1. How to do rapid HIV screening tests & controls | | | Y |
| 1. STI symptomatic management and treatment | | | Y |
| 1. Counseling for prevention of STIs | | | Y |
| 1. Counseling for prevention of HIV/AIDS | | | Y |
| 1. Counseling/social support for HIV/AIDS infected clients | | | Y |
| 1. Medical management of HIV/AIDS infected clients | | | Y |
| 1. Antiretroviral therapy for HIV infected clients | | | Y |
| 1. HIV and infant feeding counseling | | | Y |
| 1. Family planning | | | Y |
| **SECTION 3: SERVICES OFFERRED** | | | |
| **Q301: Please ask to see the MCH/FP and ART clinic and indicate which of the following activities are routinely carried out there** | | | |
| **ASK: Is this (read a-o) service usually available to client at the MCH/FP section or VCT or CCC/ART clinic?** | **Available at MCH/FP unit (circle as appropriate)** | **Available in the VCT or CCC/ART clinic (circle as appropriate)** | |
|  | **Yes** | **Yes** | |
| 1. Ante-natal care | Y | Y | |
| 1. PMTCT | Y | Y | |
| 1. Post-natal care | Y | Y | |
| 1. Family Planning | Y | Y | |
| 1. HIV/AIDS Counseling | Y | Y | |
| 1. HIV/AIDS testing services | Y | Y | |
| 1. CD4 count services | Y | Y | |
| 1. Antiretroviral therapy (ART) | Y | Y | |
| 1. STI counseling | Y | Y | |
| 1. STI laboratory services | Y | Y | |
| 1. STI Syndromic diagnosis | Y | Y | |
| 1. STI treatment | Y | Y | |
| 1. TB screening and testing | Y | Y | |
| 1. TB treatment | Y | Y | |
| 1. Screening for cancer of the cervix | Y | Y | |

| **Q302: ASK:** **Which of these FP methods are usually available to client at the MCH/FP section or VCT or CCC/ART clinic** | **Available at MCH/FP unit**  **(circle as appropriate)** | **Available in the facility VCT or CCC/ART clinic**  **(circle as appropriate)** |
| --- | --- | --- |
|  | **Yes** | **Yes** |
| 1. Combined pill | Y | Y |
| 1. Progestin only pill | Y | Y |
| 1. Injectable | Y | Y |
| 1. Male/female condom | Y | Y |
| 1. IUCD | Y | Y |
| 1. Hormonal implants | Y | Y |
| 1. Dual protection | Y | Y |
| 1. Female sterilization | Y | Y |
| 1. Male Sterilization | Y | Y |
| 1. LAM | Y | Y |
| 1. Natural FP methods | Y | Y |
| 1. Others | Y | Y |
| 1. Other Specify: |  |  |

| **Q303: Are these services routinely performed in the Family Planning Unit or ART clinic?** | | | | | | |
| --- | --- | --- | --- | --- | --- | --- |
| ***Please circle appropriate response*** | | **FP Clinic** | | | **ART Clinic** | |
|  |  | **Yes** | | | **Yes** | |
| 1. Conducting group health discussion sessions | | Y | | | Y | |
| 1. Weighing of clients | | Y | | | Y | |
| 1. Measuring blood pressure | | Y | | | Y | |
| **Q304: Are these procedures performed in the facility** | | | | | | |
| 1. Female sterilization | | Y | | |  | |
| 1. Male sterilization | | Y | | |  | |
| **Q305: Where are these services routinely performed?** | | | | | | |
|  | | **MCH/FP Clinic** | | **VCT or CCC/ART clinic** | **Elsewhere** | |
| 1. Urine test | | Y | | Y | Y | |
| 1. Pregnancy test | | Y | | Y | Y | |
| 1. HB testing | | Y | | Y | Y | |
| 1. VDRL or syphilis test | | Y | | Y | Y | |

| **Q306: Where are HIV tests conducted in this facility?** | |
| --- | --- |
| 1. In the outpatient unit | Y |
| 1. In the MCH/FP clinic | Y |
| 1. In the inpatient wards | Y |
| 1. In PMTCT clinic | Y |
| 1. VCT centre | Y |
| 1. ART clinic | Y |
| 1. Laboratory only | Y |

| **SECTION 4: AVAILABILITY OF COMMODITIES, EQUIPMENT, SUPPLIES ETC** | | | | | | | |
| --- | --- | --- | --- | --- | --- | --- | --- |
| **Q401: ASK to see the stocks of the following commodities (supplies or equipment for performing…) are currently available at the MCH/FP section and the VCT or CCC/ART clinic?** | | | | **MCH/FP unit**  **(circle as appropriate)** | | **VCT or CCC/ART clinic**  **(circle as appropriate)** |  |
|  |  |  |  | **Yes** | | **Yes** |  |
| 1. Combined pill | | | | Y | | Y |  |
| 1. Progestin only pill | | | | Y | | Y |  |
| 1. Injectable | | | | Y | | Y |  |
| 1. Male /female condom | | | | Y | | Y |  |
| 1. IUCD | | | | Y | | Y |  |
| 1. Hormonal implants | | | | Y | | Y |  |
| 1. Dual protection | | | | Y | | Y |  |
| 1. Female sterilization | | | | Y | | Y |  |
| 1. Male Sterilization | | | | Y | | Y |  |
| k) Others | | | | Y | | Y |  |
| l) Other Specify | | | |  | |  |  |
| **Which of these are available at MCH/FP or CCC/ART clinic or anywhere in the facility** | **MCH/FP unit**  **(circle as appropriate)** | | | **VCT or CCC/ART clinic**  **(circle as appropriate)** | | **Elsewhere** |  |
| **Q402: Testing Reagents**  **Yes**  **No**  **Yes**  **No** | | | | | |  |  |
| 1. Reagents for HIV (**Elisa HIV-1**) | Y | | Y | | | Y |  |
| 1. Reagents for HIV (**Elisa HIV-2**) | Y | | Y | | | Y |  |
| 1. Rapid reagents for HIV: **UNIGOLD** | Y | | Y | | | Y |  |
| 1. Rapid reagents for HIV: D**ETERMINE** | Y | | Y | | | Y |  |
| 1. Reagents for anemia test (Hb/hemocrit/PCV) | Y | | Y | | | Y |  |
| 1. Reagents for TB tests | Y | | Y | | | Y |  |
| 1. Reagents for pregnancy test | Y | | Y | | | Y |  |
| **Which of these are available at MCH/FP or CCC/ART clinic or anywhere in the facility** | | **MCH/FP unit**  **(circle as appropriate)** | | | | **VCT or CCC/ART clinic**  **(circle as appropriate)** |  |
| **Q403: General supplies** | | | | | | |  |
| 1. Disposable needles and syringes | | Y | | | Y | |  |
| 1. Disposable gloves | | Y | | | Y | |  |
| 1. Specimen bottles for urine | | Y | | | Y | |  |
| 1. Specimen pots for sputum | | Y | | | Y | |  |
| 1. Blood specimen pots | | Y | | | Y | |  |
| 1. Reagents for UTI | | Y | | | Y | |  |
| **Q404: Drugs** | | | | | | |  |
| 1. Nevirapine tabs | | Y | | | Y | |  |
| 1. Nevirapine syrup | | Y | | | Y | |  |
| 1. Zidovudine (ZDV, AZT) | | Y | | | Y | |  |
| 1. AZT syrup | | Y | | | Y | |  |
| 1. Zidovudine + Lamivudine (Combivir) | | Y | | | Y | |  |
| 1. Metronidazole tablets | | Y | | | Y | |  |
| 1. Miconazole or clotrimazole pessaries | | Y | | | Y | |  |
| 1. Ciprofloxacin oral | | Y | | | Y | |  |
| 1. Erythromycin oral | | Y | | | Y | |  |
| 1. Tetracycline oral | | Y | | | Y | |  |
| 1. Benzathine Penicillin | | Y | | | Y | |  |
| 1. Contrimoxazole tabs | | Y | | | Y | |  |
| 1. Contrimoxazole syrup | | Y | | | Y | |  |
| 1. List other HIV/AIDS drugs available in the facility | | Y | | | Y | |  |

| **Q405: For the Family Planning Clinic, are the following items in the room or somewhere in the clinic?** | | |
| --- | --- | --- |
|  | Yes |  |
| 1. Spotlight or flashlight or examination light | Y |  |
| 1. Examination couch | Y |  |
| 1. Sterile latex gloves | Y |  |
| 1. Clean latex gloves | Y |  |
| 1. Clean non latex gloves | Y |  |
| 1. Decontamination solution (chlorine based) for clinical equipment | Y |  |
| 1. Waste receptacle with lid and plastic liner | Y |  |
| 1. Container for used sharps | Y |  |
| 1. Single use hand drying towels or a functioning electric hand dryer | Y |  |
| 1. Running water | Y |  |
| 1. A working blood pressure machine | Y |  |
| 1. A stethoscope | Y |  |
| 1. A functional weighing scale | Y |  |
| 1. Speculum (S) | Y |  |
| 1. Speculum (M) | Y |  |
| 1. Speculum (L) | Y |  |
| 1. Tenacula | Y |  |
| 1. Uterine sound | Y |  |
| 1. Autoclave | Y |  |
| 1. Cleaning solution e.g. betadine | Y |  |
| 1. Troca | Y |  |
| 1. Gauze | Y |  |
| 1. Surgical scissors | Y |  |
| 1. Elastoplast | Y |  |
| 1. Kidney dishes | Y |  |
| 1. Sponge holding forceps | Y |  |
| 1. Mosquito forceps - curved | Y |  |
| 1. Mosquito forceps - straight | Y |  |
| 1. Surgical blade - size 15 or 11 | Y |  |
| 1. Draping towels | Y |  |

| **SECTION 5: PAYMENT/FEES** | | |
| --- | --- | --- |
| **Q501: For each of the following items, indicate if there is routine fee and if yes the amount** | **Yes** | **Amount in Kshs** |
| 1. Fee for FP client records (card and file) | Y |  |
| 1. Fee for consultation | Y |  |
| 1. Pregnancy test | Y |  |
| 1. IUCD insertion | Y |  |
| 1. Oral contraceptives/pills | Y |  |
| 1. Male condom | Y |  |
| 1. Female condom | Y |  |
| 1. Injectable methods | Y |  |
| 1. Implants | Y |  |
| 1. Emergency contraceptives | Y |  |
| 1. Male sterilization | Y |  |
| 1. Female sterilization | Y |  |
| 1. Others | Y |  |
| 1. Other specify; |  |  |

| **SECTION 6: IEC MATERIAL** | | |
| --- | --- | --- |
| **Q601: Are any of the following visual aids for teaching available in the counseling rooms** | **FP Clinic** | **ART Clinic** |
|  | **Yes** | **Yes** |
| 1. Samples of various FP methods | Y | Y |
| 1. Visual aids for teaching about STIs | Y | Y |
| 1. Visual aids for teaching about HIV/AIDS | Y | Y |
| 1. Balanced counseling strategy cards | Y | Y |
| 1. Model for demonstrating how to use condoms | Y | Y |
| 1. Posters about FP | Y | Y |
| **Q602: Are any of the following types of information booklets or pamphlets available in the counseling or consultation rooms for clients to take home** | **FP Clinic** | **ART Clinic** |
|  | **Yes** | **Yes** |
| 1. Printed materials on FP | Y | Y |
| 1. Printed materials on STIs | Y | Y |
| 1. Printed materials on HIV/AIDS | Y | Y |

| **SECTION 7: GUIDELINES, POLICIES AND STANDARDS** |  |  |
| --- | --- | --- |
| **Are any of the following protocols for delivery of services available in the consultation/counseling rooms** | **FP Clinic** | **ART Clinic** |
|  | **Yes** | **Yes** |
| 1. FP policy guidelines for service providers | Y | Y |
| 1. Guidelines for making a syndromic diagnosis of STIs and their treatment | Y | Y |
| 1. PMTCT guidelines | Y | Y |
| 1. Guidelines to Antiretroviral Drug Therapy (ART) | Y | Y |
| 1. Clinical manual for ARV providers | Y | Y |
| 1. Is there an official guideline/protocol on HIV testing procedures in this facility? | Y | Y |
| 1. Is there a pre and post test counseling protocol for HIV testing | Y | Y |

| **SECTION 8: DATA COLLECTION TOOLS** |  |  |
| --- | --- | --- |
| **Are any of the following data collection tools available in the consultation/counseling rooms** | **FP Clinic** | **ART Clinic** |
|  | **Yes** | **Yes** |
| 1. Is there a register where information on FP clients’ visits or referrals is recorded? **Show as subset question** (**If yes,** for the register to be valid it must show client’s status (new or revisit) | Y | Y |
| 1. Do you have Family Planning Cards in stock | Y | Y |
| 1. Is there a register where information on HIV clients is recorded? **Show as subset question** (**if yes,** for the register to be valid it must show status (new or continuing) | Y | Y |
| 1. Do you have a referral document for the HIV positive clients e.g referral form | Y | Y |

| **SECTION 9: INFRASTRUCTURE** | |  |
| --- | --- | --- |
| **Infrastructure available in FP and ART units** | **MCH/FP** | **ART clinic** |
|  | **Yes** | **Yes** |
| 1. Waiting area is shaded and with seats | Y | Y |
| 1. Private space for FP examination | Y | Y |
| 1. Source of clean water in the clinic 24 hours | Y | Y |
| 1. Power to ensure fridge remains functional 24 hours/day | Y | Y |
| 1. Working autoclave/sterilization | Y | Y |
| 1. Reliable lighting | Y | Y |
| 1. Client toilets | Y | Y |
| 1. Clean water for drinking | Y | Y |
| 1. Clean cups/glasses for drinking water | Y | Y |

# Appendix 8.4: Provider Knowledge

| DRAFT  **Assessing the Benefits of integrated HIV and Reproductive Health Services in Kenya**  **PROVIDER KNOWLEDGE**  **PRE-INTERVENTION**  **Central Province** |
| --- |

**Questionnaire Number**

FACILITY name .................................. DISTRICT.........................................

**FACILITY CATEGORY**

(1. Hospital 2.Health centre 3. Dispensary 4. IPPF clinic )

**Date of assessment**: Day/Month/Year:

**Name of Data Collector** …………………………………………………………... Code:

**Time Started:** (Use 24 hours clock)

**Time finished:**

**Supervisor’s review**: Name ___________________ Signature

_________________Date__________

| **Q100. Record the type of provider who attended to the client.** | | | | | |
| --- | --- | --- | --- | --- | --- |
|  | Enrolled Nurse/Midwife (Certificate) | | | | Y |
|  | Registered Nurse/Midwife (Diploma) | | | | Y |
|  | Degree Nurses (Degree e.g. BScN) | | | | Y |
|  | Clinical Officers (Diploma) | | | | Y |
|  | Medical Officers (Degree) | | | | Y |
|  | Other, specify | | | | Y |
| Q101 | | For how long have you worked in this clinic? | Indicate in Months | | |
| **Q102. ASK THE FOLLOWING QUESTION FOR EACH SPECIFIC SUBJECT: Have you received updates in (SUBJECT) as a part of IN – SERVICE training?** | | | | | |
|  | | Family planning | | | Y |
|  | | Contraceptive technology e.g. Implants | | | Y |
|  | | FANC | | | Y |
|  | | Syndromic diagnosis and management of STIs | | | Y |
|  | | Prevention of Mother-to-child transmission of HIV/AIDS (PMTCT) | | | Y |
|  | | Voluntary Counseling and Testing of HIV/AIDS | | | Y |
|  | | Anti-retroviral therapy for HIV/AIDS infected clients | | | Y |
|  | | Management of TB in HIV+ family planning clients | | | Y |
|  | | Medical Eligibility Criteria for Contraceptive use | | | Y |
|  | | Integration of Counseling and Testing for HIV into FP | | | Y |
|  | | Other, specify……………………………………………………………… | | | Y |
| Q103 | | Can you comfortably counsel a client for HIV testing? | | | Y |
| Q104 | | Can you comfortably test a client for HIV using the rapid test kit? | | | Y |
| Q105 | | Have you seen the Balanced Counseling Strategy cards? | | | Y |
| Q106 | | Do you ever use the BCS cards to provide services to your clients? | | | Y |
| **ASK THE FOLLOWING QUESTION FOR EACH SPECIFIC SUBJECT: Have you received updates in (SUBJECT) as a part of IN – SERVICE training?** | | | | | |
| **Q107. What are the four prongs/pillars of WHO to reduce MTCT? *(Do not read. Wait for spontaneous response)*** | | | | |  |
|  | | Primary prevention of HIV infection | | | Y |
|  | | Prevention of unintended pregnancies among HIV-infected women | | | Y |
|  | | Prevention of HIV transmission from HIV-infected women to their children | | | Y |
|  | | Provision of care for HIV-infected mothers and their infants | | | Y |
| **Q108.** | In your opinion, is integration of CT to FP feasible and practical in our health care system? | | | | **Y** |
| **Q109** | If yes, Why? | | | | |
|  | If not, what are the challenges   1. …………………………………………………………………… 2. …………………………………………………………………… 3. …………………………………………………………………… | | |  | |

| **Q110** | ***As a health provider clients will expect you to explicitly counsel and inform them on the various methods of FP available in the clinic. Your discussion will focus on the contraindications of the method, how the method works and the side effects. I would likely ask you to tell me the contraindications, and side effects of some of these methods (Do not read. Circle what is mentioned)*** | |
| --- | --- | --- |
| **Q110b** | **When is condom use Contraindicated? *(Do not read. Circle what is mentioned)***   1. Allergy to the rubber 2. Others specify.................................................. | **Y**  **Y** |
| **Q111** | **Clients who use condom must be informed on how to correctly use it. What should the client know about condoms use?** | |
|  | Should be put on an erect penis before entrance to vagina | **Y** |
|  | Use a new condom for a new coitus | **Y** |
|  | Remove after coitus while penis is still erect | **Y** |
|  | Should not be exposed to high temperatures or sunlight | **Y** |
|  | Do not use condoms after 3 years beyond date of manufacture | **Y** |
|  | Before use, check condom package for air tightness | **Y** |
|  | Tear open packages with hands. Do not use teeth, scissors, razors, or other sharp object | **Y** |
|  | Use only water or water-based lubricant (oil or Vaseline can damage it) | **Y** |
|  | Use only water or water-based lubricant (oil or Vaseline can damage it) | **Y** |
| **Q112** | **When are Combined Oral Contraceptives contra indications? *(Do not read. Circle what is mentioned)*** | |
|  | Surgery or any event that will keep her in bed for many days | **Y** |
|  | Swelling in breasts | **Y** |
|  | Breast cancer in her family | **Y** |
|  | History of cervical cancer in her family | **Y** |
|  | Jaundice (yellow eyes). | **Y** |
|  | Cardiovascular illness (heart disease) | **Y** |
|  | History or presence of unexplained vaginal bleeding | **Y** |
|  | Currently breastfeeding a child less than 6 month-old | **Y** |
|  | Client’s smoking status (more than 15 cigarettes / daily) | **Y** |
|  | Use of specific medicines (as for tuberculosis or epilepsy) | **Y** |
|  | | |
| **Q113** | What do you advise a client to do if she misses to take her pill for one day? | |
| **Q114** | What do you advise a client to do if she misses to take her pill for 2 days? | |
| **Q115** | What do you advise a client to do if she misses to take her pill for 3days? | |
| **Q116. Contraindications of IUCD contraceptives use include: *(Do not read. Circle what is mentioned)*;** | | |
|  | Possible pregnancy | **Y** |
|  | Unexplained or abnormal vaginal bleeding | **Y** |
|  | Client has more than one sexual partner | **Y** |
|  | Client’s partner has more than one sexual partner ( multiple partners) | **Y** |
|  | Presence of genital tract infections | **Y** |
|  | STD or pelvic inflammatory disease in the past 3 months | **Y** |
|  | Genital tract cancer | **Y** |
| **Q117** | **The side effects of IUCD include: *(Do not read. Circle what is mentioned)*;** | |
|  | Increased menstrual cramps | **Y** |
|  | Increased or irregular bleeding | **Y** |
| **Q118** | **Injectable contraceptives contra indication include; *(Do not read. Circle what is mentioned)*** | |
|  | Possible pregnancy | **Y** |
|  | Abnormal or unexplained vaginal bleeding | **Y** |
|  | Current or past breast cancer | **Y** |
|  | Evidence of liver disease(yellow eyes) | **Y** |
| **Q119** | **Side effects of Injectables** | |
|  | Altered menstrual flow (spotting, amenorrhoea, heavier bleeding) | **Y** |
|  | Weight changes ( weight gain or loss) | **Y** |
|  | Dizziness or nausea | **Y** |
|  | Headaches | **Y** |
|  | Return to fertility may be delayed up to 6 months once method is discontinued | **Y** |
| **Q120** | **Which method do you usually recommend in particular for new postpartum women who are HIV+?** | |
|  | Combined pill (ovral, triphasil, nordett) | **Y** |
|  | Progestin-only pill (microval) | **Y** |
|  | IUCD | **Y** |
|  | Condom (male or female) | **Y** |
|  | Injectable (nuristerate or depo | **Y** |
|  | Hormonal implant (jadelle etc) | **Y** |
|  | Female sterilization | **Y** |
|  | Male sterilization | **Y** |
|  | Emergency contraception | **Y** |
|  | Dual protection method (condom +) | **Y** |
|  | LAM | **Y** |
|  | Nothing – no method | **Y** |
|  | Other specify | **Y** |
| **Q121** | **What kind of information would you give to HIV positive mothers and family about care and support that is available to them?** | |
|  | Follow up appointments | **Y** |
|  | Cotrimoxazole for baby | **Y** |
|  | Immunisation for baby | **Y** |
|  | Growth monitoring for baby | **Y** |
|  | Family planning | **Y** |
|  | Management of opportunistic infections | **Y** |
|  | Support groups & psycho social support that is available | **Y** |
|  | Adherence support groups | **Y** |
|  | Community support | **Y** |
|  | ART | **Y** |
|  | Other specify | **Y** |
| **Q122** | **What records do you keep to facilitate referral to and between other units such as CCC or art clinic?** | |
|  | None | **Y** |
|  | Referral slips | **Y** |
|  | Registers | **Y** |
|  | Recorded on patients cards | **Y** |

**Time of observation ended (in 24 hour clock)**

**Thank the provider**

| **Assessing the Benefits of integrated HIV and Reproductive Health Services in Kenya**  **CLIENT COHORT INTERVIEW**  **Central Province** |
| --- |

Appendix 8.5: client cohort **DRAFT**

**Questionnaire Number**

FACILITY name …………………. District ……………………..

**FACILITY CATEGORY**

1. Hospital
2. Health centre
3. Dispensary
4. Private clinic

**Date of assessment**: Day/Month/Year:

**Name of Data Collector** …………………………………………………………... Code:

**Time Started:** (Use 24 hours clock)

**Supervisor’s review**: Name ___________________ Signature _________________Date__________

**Order of interview being undertaken:**

1. First Interview on recruitment
2. Second interview at 6 months
3. Third Interview at 18 months
4. Fourth Interview at 30 months

| ***I am going to ask you a few short questions. Thank you for taking the time to help us with our study today. Please remember that this information will be kept strictly confidential and your name will not be recorded anywhere on this form.*** | | | | | | | | | | | | | | | | |
| --- | --- | --- | --- | --- | --- | --- | --- | --- | --- | --- | --- | --- | --- | --- | --- | --- |
| **Section 1 background socio-demographic information** | | | | | | | | | | | | | | |  |  |
|  | | | Sex of client | | | | | 1. Male 2. Female | | |  | | | |  |  |
|  | | | How old are you? (age in complete in years) | | | | |  | | |  | | | |  |  |
|  | | | What is your current marital status? | | | | | 1. Married monogamous 2. Married polygamous 3. Living together 4. Single 5. Divorced/separated/widowed 6. Others | | |  | | | |  |  |
|  | | | What is the highest level of school that you have attained? | | | | | 1. None 2. Primary 1 to 4 3. Primary 5 to 8 4. Secondary 5. College/tertiary | | |  | | | |  |  |
|  | | | What is your current employment? | | | | | 1. Professional/ technical/ managerial 2. Clerical 3. Sales and services 4. Skilled manual 5. Un skilled manual 6. Domestic service 7. Agriculture 8. Unemployed 9. Other, specify............................. | | |  | | | |  |  |
|  | | | Was this your first visit to this clinic? | | | | | 1. Yes 2. No | | |  | | | |  |  |
|  | | | How many times have you been before | | | | | times | | |  | | | |  |  |
|  | | | How long did it take you get to the clinic today? | | | | | hours  minutes | | |  | | | |  |  |
|  | | | Where do you live? *(write name). Need to be specific, location, division , rural/urban* | | | | | .................................. | | |  | | | |  |  |
|  | | | How long did you have to wait today to be seen by the nurse or doctor? *(first consultation only if queued more than once)* | | | | | hours  minutes | | |  | | | |  |  |
| **Section 2 FP history** | | | | | | | | | | | | | | |  |  |
|  | What was the main (first) reason that you visit the facility today or your latest visit?  (**New**:- new to clinic, new to FP following gap in use of FP,) | | | | | 1. Family planning (new client) 2. Family planning (resupply of method) 3. FP client switching method 4. FP method after childbirth 5. Other (specify): | | | |  | | | | |  |  |
|  | How many different nurses or doctors did you actually see today/ last visit to facility? | | | | | --------------------------insert number | | | |  | | | | |  |  |
|  | What did you see them for? *(insert type of service )* | | | | | 1st …………………………..  2^nd^…………………………..  3^rd^…………………………..  4^th^…………………………..  5^th^ ………………………….. | | | | | | | | |  |  |
|  | Did you attend any group health talks at the facility today / last visit to the facility? | | | | | 1. Yes 2. No | | | Skip to Q16 | | | | | |  |  |
|  | What was the subject of the group talk?  *Circle all that apply* | | | | | 1. Family planning 2. HIV education 3. HIV testing counseling 4. Other (specify): | | |  | | | | | |  |  |
|  | Please could you tell me; have you ever been pregnant? | | | | | 1. Yes 2. No | | | Skip Q22 | | | | | |  |  |
|  | How many times have you been pregnant? | | | | | number | | |  | | | | | |  |  |
|  | How many living children do you have? | | | | | number | | | If none, skip to Q20 | | | | | |  |  |
|  | How old is your youngest child? | | | | | months  years | | |  | | | | | |  |  |
|  | Were you using a contraceptive method when you became pregnant with your last child? | | | | | 1. Yes 2. No | | | Skip to Q22 | | | | | |  |  |
|  | If yes, which method were you using?    **(Circle only one method)** | | | | | 1. Progestin only pills 2. Combined oral contraceptive pill 3. Emergency contraception 4. Injectable 5. Male condom 6. Female condom 7. Hormonal implant 8. IUD 9. Female sterilization (BTL) 10. Vasectomy 11. Lactational Amenorrhea Method 12. Natural Family Planning Method 13. Other (specify) | | |  | | | | | |  |  |
|  | Would you like to have (a/another) child, or would you prefer not to have any (more) children? | | | | | 1. Have a/another child 2. No more children 3. Undecided /don’t know | | | Skip to Q24  Skip to Q24 | | | | | |  |  |
|  | How long would you like to wait from now before the birth of (a/another) child?  (if does not know probe) | | | | | 1. [ ] [ ] months 2. [ ] [ ] years 3. Cannot get pregnant 4. After marriage 5. Other specify | | |  | | | | | |  |  |
|  | What is your desired family size? | | | | | number | | |  | | | | | |  |  |
|  | Is this the same as your husband /partner? | | | | | 1. Wants same number of children as partner 2. Partner wants more children 3. Partner wants less children 4. Partner does not want any children 5. Don’t know | | |  | | | | | |  |  |
|  | Could you please tell me who referred you for family planning services | | | | | 1. Health worker 2. Self referral 3. Family member 4. Friend 5. Other specify | | |  | | | | | |  |  |
|  | Were you already using a method of contraception when you arrived here today or visit the FP clinic the last time? | | | | | 1. Yes 2. No | | | Skip to Q30 | | | | | |  |  |
|  | Which method(s) did you use last?  (prior to the consultation today) | | | | | 1. Progestin only pills 2. Combined oral contraceptive pill 3. Emergency contraception 4. Injectable 5. Male condom 6. Female condom 7. Hormonal implant 8. IUD 9. Female sterilization (BTL) 10. Vasectomy 11. Lactational Amenorrhea Method 12. Natural Family Planning Method 13. Other specify.................... | | | Skip Q29  Skip Q29 | | | | | |  |  |
|  | Are (were) you using a condom with this method | | | | | 1. Yes 2. No | | |  | | | | | |  |  |
|  | Please can you confirm which FP methods you were given today?  **(circle all that are mentioned)**  *(See chart for brand names of different pills)* | | | | | 1. Progestin only pills 2. Combined oral contraceptive pill 3. Emergency contraception 4. Injectable 5. Male condom 6. Female condom 7. Hormonal implant 8. IUD 9. Female sterilization (BTL) 10. Vasectomy 11. Lactational Amenorrhea Method 12. Natural Family Planning Method 13. Other specify.................... | | | |  | | | | |  |  |
|  | Is this your preferred method? | | | | | 1. Yes 2. No | | | | Skip | | | | |  |  |
|  | Why do you prefer this method? | | | | | Write in............................ | | | |  | | | | |  |  |
|  | Why didn’t you get your preferred method | | | | | Write in------------------------------- | | | |  | | | | |  |  |
|  | Did you change methods today/ last time you visited the clinic? | | | | | 1. Yes 2. No | | | | Skip | | | | |  |  |
|  | Please tell me what you would do if you missed to take your routine pill for one day. | | | | | | | | | | | | | |  |  |
|  | Please tell me what you would do if you missed to take your routine pill for 2 days.. | | | | | | | | | | | | | |  |  |
|  | Please tell me what you would do if you missed to take your routine pill for 3 days.. | | | | | | | | | | | | | |  |  |
| **SECTION 3: SEXUAL BEHAVIOUR( reassure the client that some of the questions may be sensitive but confidentiality will be maintained)** | | | | | | | | | | | | | | |  |  |
|  | How many times have you had sex in the last month? | | | 1. [ ] [ ] number 2. Never had sex | | | | | |  | | | | |  |  |
|  | The last time you had sexual intercourse, was a condom used? | | | 1. Yes 2. No | | | | | | Skip to Q41 | | | | |  |  |
|  | What was the main reason you used a condom on that occasion? | | | 1. Wanted to prevent STI/HIV 2. Wanted to prevent pregnancy 3. Wanted to prevent both STI/HIV and pregnancy 4. Did not trust partner/feels partner has other partners 5. Partner insisted 6. Don’t know 7. Other (SPECIFY) | | | | | |  | | | | |  |  |
|  | Do you think your partner has other partners? | | | 1. Yes 2. No 3. Don’t know | | | | | |  | | | | |  |  |
|  | Other than this partner, have you had sex with anyone else in the last 12 months? | | | 1. Yes 2. No | | | | | |  | | | | |  |  |
|  | In the last 6, 12, 12 months with how many people overall have you had sex with? | | | NUMBER | | | | | |  | | | | |  |  |
| SECTION 4: KNOWLEDGE OF SEXUALLY TRANSMITTED INFECTIONS AND HIV | | | | | | | | | | | | | | | |  |
|  | | | As far as you know, are there any diseases that can be transmitted through sexual intercourse? | | | | | 1. Yes 2. No 3. Don’t know | | | | |  | | |  |
|  | | | As far as you know, what are the ways you can become infected with HIV/AIDS?  (Circle all that are mentioned) | | | | | 1. Unprotected sexual intercourse 2. From mother to child during pregnancy 3. From mother to child during birth 4. Breastfeeding 5. Blood transfusion 6. Contact with infected blood 7. Others (specify): | | | | |  | | |  |
|  | | | Do you know of ways of protecting yourself from STI/HIV and AIDS?  (CIRCLE ALL that are mentioned) | | | | | 1. Use a condom 2. Stick to one partner 3. Abstain from sex 4. Male circumcision 5. Thigh sex 6. Others (specify) | | | | |  | | |  |
| 42 | | | Please can you tell me, have you ever been tested for HIV? | | | | | 1. Yes 2. No | | | | | Skip to Q54 | | |  |
| 43 | How many times have you tested for HIV? | | | | | | Write in number | | | | | |  | |  |  |
| 44 | Now I would like you to tell me where you received the (last) HIV test?  (if tested more than once ask about the most recent test) | | | | | | 1. ANC clinic 2. PNC clinic 3. VCT clinic 4. FP clinic 5. Postnatal ward 6. Other specify……………… 7. Dont know/can’t remember | | | | | |  | |  |  |
| 45 | | | Would you feel comfortable returning to the health provider who gave you your results if you have any questions about HIV? | | | | | 1. Yes 2. No 3. Don’t know | | | | |  | |  |  |
| 46 | | | Please can you tell me:  What was the date of your last test? (write today’s date if applicable) | | | | | Write approximate date  Month  Year | | | | |  | |  |  |
| 47 | | | Did you share your results with anyone? | | | | | 1. Yes 2. No | | | | | Skip to Q52 | |  |  |
| 48 | | | Please can you tell me who was the first person you shared your HIV test results with? | | | | | 1. Partner 2. Mother 3. Father 4. Female relative 5. Male relative 6. Friend 7. Other health provider | | | | |  | |  |  |
| 49 | | | If you did not share your results with anyone – please can you tell me why? | | | | | Write in.................................. | | | | |  | |  |  |
| 50 | | | Would you be prepared to tell me your HIV status?  Please know that I will keep this information confidential | | | | | 1. HIV Negative 2. HIV Positive 3. HIV test done but not received results 4. Does not want to disclose results | | | | |  | |  |  |
| 51 | | | Has your partner been tested for HIV? | | | | | 1. Yes 2. No 3. Don’t know | | | | |  | |  |  |
| 52 | | | Has he shared his results with you? | | | | | 1. Yes 2. No | | | | |  | |  |  |
| **Section 5 FOR HIV positive clients only** | | | | | | | | | | | | | | |  |  |
| 53 | | | **For HIV positive clients only:**  When you received your results did the provider refer you to the ART clinic for follow-up? | | | 1. Yes 2. No 3. Don’t know | | | | | | |  | |  |  |
| 54 | | | **For HIV positive clients only:**  If yes which ART clinic were you referred to? | | | 1. Within the same facility 2. Another facility 3. Other specify | | | | | | |  | |  |  |
| 55 | | | **For HIV positive clients only:**  Have you been to the ART clinic yet? | | | 1. Yes 2. No | | | | | | |  | |  |  |
| 56 | | | **For HIV positive clients only:**  Have you ever had your CD4 count done? | | | 1. Yes 2. No 3. Don’t know | | | | | | | Skip to Q61  Skip | |  |  |
| 57 | | | **For HIV positive clients only:**  Do you know what your CD4 count is? | | | 1. Yes , write in count [ ] 2. CD 4 count done but does not know level | | | | | | |  | |  |  |
| 58 | | | **For HIV positive clients only:**  Are you on any treatment? | | | 1. Drugs for opportunistic infection 2. Pre –HAART Prophylaxis 3. HAART 4. TB treatment 5. Not on any treatment 6. Other specify | | | | | | |  | |  |  |
| 59 | | | **For HIV positive clients only:**  Are you enrolled with any support groups | | | 1. Yes 2. No | | | | | | |  | |  |  |
| 60 | | | **For HIV positive clients only**  If yes which ones? | | | Write in -------------------------------- | | | | | | |  | |  |  |
| 61 | | | **For HIV positive clients only:**  If you have children, has any of your children/child been tested for HIV? | | | 1. All tested 2. Some tested (if applicable) 3. None tested 4. Do not have children | | | | | | | Skip to Q67  Skip to Q67 | |  |  |
| 62 | | | **For HIV positive clients only:**  Are any of your children HIV positive? | | | 1. All are HIV positive 2. Some are HIV positive 3. None are HIV positive 4. Don’t know | | | | | | | Skip to Q67  Skip to Q67 | |  |  |
| 63 | | | **For HIV positive clients only:**  Are your children/Is your child receiving care in this clinic? | | | 1. None are 2. All are 3. Some are 4. Don’t know | | | | | | |  | |  |  |

Time of observation ended (in 24 hour clock)

Thank the client for taking the time to do the interview

# Appendix 8.6: Community Survey of Women

**IPPF Research Project on the
Integration of Sexual and Reproductive Health Services**

**Kenya 2008-2012**

**CONFIDENTIAL**

**Community Survey of Women (BASELINE)**

**BASELINE QUESTIONNAIRE**

**CLUSTER NUMBER:**

**HOUSEHOLD NUMBER:**

**INTERVIEWER’S CODE:**

**DATE OF INTERVIEW (dd-mm-yy): --**

**TIME STARTED (hh-mm): :**

**TIME ENDED: :**

| **SECTION 1: BACKGROUND INFORMATION** | | | | |
| --- | --- | --- | --- | --- |
| **No.** | **Questions and filters** | **Coding categories** | | **Skip to** |
| 1.1 | What is your date of birth | [ ] [ ] [ ] [ ] 19 [ ] [ ]  D D M M Y Y  Don’t know | 99 |  |
| 1.2 | What is your current marital status? | Single  Married monogamous  Married polygamous  Living with a partner  Divorced / separated / widowed  Others | 1  2  3  4  5  6 |  |
| 1.3 | What is the highest level of school you attended?  (or are still attending if at school) | None  Primary 1 to 4  Primary 5 to 8  Secondary  College / tertiary  Others | 1  2  3  4  5  6 |  |
| 1.4 | What is your religion? | Christian (Protestant)  Christian (Roman Catholic)  Christian (Pentecostal)  Islam  Zionist  Traditional  None  Other (specify)_________________________________________ | 1  2  3  4  5  6  7  8 |  |
| 1.5 | How would you describe your present employment situation? | Unemployed, looking for work  Unemployed, not looking for work  Work in informal sector, not looking for permanent work  Sick / disabled and unable to work  Student  Self-employed - full time (40 hours or more per week)  Self-employed - part time (less than 40 hours per week)  Employed part time (if none of the above) (less than 40 hours per week)  Employed full time (40 hours or more per week)  Other specify)_________________________________________ | 1  2  3  4  5  6  7  8  9  10 |  |
| 1.6 | Do you have enough money to meet your basic needs for food, clothes, health care and accommodation? | Not at all  A little  Moderately  Mostly  Completely | 1  2  3  4  5 |  |
| 1.7 | Does your household own any of the following in working condition:  *Circle all those that apply* | Television  Video / DVD player  Radio  Telephone / Mobile phone  Refrigerator  Microwave  Bicycle  Motorbike / scooter  Car | 1  1  1  1  1  1  1  1  1 |  |

| **SECTION 2: FERTILITY PREFERENCE & HIV** | | | | |
| --- | --- | --- | --- | --- |
| *Now I would like to ask you some very private information. Please be assured that what you tell me will be kept completely confidential and your name and address does not appear in this questionnaire.* | | | | |
| 2.1 | Do you have one regular sexual partner or do you ever have casual partners? | One regular partner  Casual partners only  Regular partner and casual partners | 1  2  3 |  |
| 2.2 | Have you had sexual intercourse in the last 1 month? | No  Yes | 0  1 |  |
| 2.3 | When do you think you may have your [first or next] child? | Within the next year  After the next 1-2 years  After 3-5 years  After 5 years from now  When I complete schooling  When I get a job  When I get married  No more children  Not applicable (can’t have / don’t want children)  Don’t know | 1  2  3  4  5  6  7  8  9  99 |  |
| 2.4 | Have you ever had an HIV test? | No  Yes  Don’t know | 0  1  99 |  |
| 2.5 | Do you know your HIV status? | Don’t know status  HIV negative  HIV positive  Awaiting results | 1  2  3  4 | 2.7  2.8 |
| 2.6 | Are you receiving monitoring or treatment in a health service? | No  Yes | 0  1 | 2.8  2.8 |
| 2.7 | Would you ever consider having an HIV test? | No  Yes  Maybe / Don’t know | 0  1  99 |  |
| 2.8 | Would you like to know where to obtain information on getting an HIV test or HIV treatment? | Yes (*give information on services)*  Maybe  No | 1  2  3 |  |
| 2.9 | Do you know if your current partner has ever had an HIV test? | No  Yes  Don’t know | 0  1  99 |  |
| 2.10 | Do you know your partner’s HIV status? | Don’t know partner’s status  HIV negative  HIV positive  Awaiting results | 1  2  3  4 | Sec 3 |
| 2.11 | Do you think your partner should have an HIV test? | No  Yes  Maybe / Don’t know | 0  1  99 |  |

| *Use a separate sheet for each partner mentioned in 2.1* | | | | |
| --- | --- | --- | --- | --- |
| **SECTION 3: CURRENT CONTRACEPTIVE USE Partner number** [ ] [ ] | | | | |
| 3.1 | Partner type | Husband / cohabiting partner  Boyfriend (not living together)  Casual acquaintance  Other (specify)____________________________ | 1  2  3  4 |  |
| 3.2 | Who makes decisions about what contraception to use in this relationship? | Partner does  I do  We both do | 1  2  3 |  |
| 3.3 | Have you ever used anything or tried in any way to delay or avoid getting pregnant?  ***Show card of methods*** | No  Yes  Don’t know | 0  1  99 | 3.6 |
| 3.4 | Are you or this partner *currently* doing something or using any method to avoid getting pregnant? | No  Yes  Don’t know | 0  1  99 | 3.6 |
| 3.5 | What are you or this partner using or doing to avoid getting pregnant?  (circle all that apply)  ***Show card*** | Pills  Injectables  Male Condoms  Female condoms  IUD  Implants  Female sterilization  Male sterilization (vasectomy)  Diaphragm  Foam/Jelly  Lactational amenorrhoea method  Rhythm method  Withdrawal  Other | 1  2  3  4  5  6  7  8  9  10  11  12  13  14 | 3.7 |
| 3.6 | What is the main reason that no method was used? | Casual partner, doesn’t care  Menopausal / had hysterectomy  Couple infertile / subfertile  Pregnant  Amenorrheic  Breastfeeding  Wanted (more) children  Respondent opposed  Partner opposed  Others opposed  Religious prohibition  Knows no method  Knows no source  Health concerns  Fear of side effects  Lack of access / too far  Costs too much  Inconvenient to use  Interferes with body’s normal processes  Other (specify)___________________________  Do not know | 1  2  3  4  5  6  7  8  9  10  11  12  13  14  15  16  17  18  19  20  99 |  |
| 3.7 | At the time of your last act of intercourse did you use a condom with this partner? | No  Yes  Don’t know | 0  1  99 |  |
| 3.8 | Would you like to know where to obtain information on delaying or avoiding your partner getting pregnant? | No  Yes *(give information on services)*  Don’t know | 0  1  99 |  |

| **SECTION 4: HEALTH SERVICE USE** | | | | | |
| --- | --- | --- | --- | --- | --- |
| I am going to read to you a list of health services or needs. Please can you tell us whether you or your partner has used this service, and if so, which facility did you attend for the service at any time in the last 1 year. | | | | | |
| **Have you ever been to the doctor, nurse or other person for:** | **4.1 Family Planning** | **4.2 ANC or PNC** | **4.3 VCT** | **4.4 HIV monitoring / ART** | **4.5 STI care** |
|  | [ ] Attended service 1  [ ] Did not attend 2  [ ] Partner attended 3  [ ] Both attended 4 | [ ] Attended service 1  [ ] Did not attend 2  [ ] Partner attended 3  [ ] Both attended 4 | [ ] Attended service 1  [ ] Did not attend 2  [ ] Partner attended 3  [ ] Both attended 4 | [ ] Attended service 1  [ ] Did not attend 2  [ ] Partner attended 3  [ ] Both attended 4 | [ ] Attended service 1  [ ] Did not attend 2  [ ] Partner attended 3  [ ] Both attended 4 |
| **If attended:** | | | | | |
| **CODING** | Where did you go [ ] [ ]  What did you go for? [ ] [ ]  How long ago [ ] [ ]  Fees: ________  Were you satisfied with  the service? [ ] [ ] | Where did you go [ ] [ ]  What did you go for? [ ] [ ]  How long ago [ ] [ ]  Fees: ________  Were you satisfied with  the service? [ ] [ ] | Where did you go [ ] [ ]  What did you go for? [ ] [ ]  How long ago [ ] [ ]  Fees: ________  Were you satisfied with  the service? [ ] [ ] | Where did you go [ ] [ ]  What did you go for? [ ] [ ]  How long ago [ ] [ ]  Fees: ________  Were you satisfied with  the service? [ ] [ ] | Where did you go [ ] [ ]  What did you go for? [ ] [ ]  How long ago [ ] [ ]  Fees: ________  Were you satisfied with  the service? [ ] [ ] |
| ***Where attended*** |  |  |  |  |  |
| 01. Clinic X  02. Clinic Y  03. Clinic Z  04. Can’t remember |  |  |  |  |  |
| ***What for*** | Where did you go [ ] [ ]  What did you go for? [ ] [ ]  How long ago [ ] [ ]  Fees: ________  Were you satisfied with  the service? [ ] [ ] | Where did you go [ ] [ ]  What did you go for? [ ] [ ]  How long ago [ ] [ ]  Fees: ________  Were you satisfied with  the service? [ ] [ ] | Where did you go [ ] [ ]  What did you go for? [ ] [ ]  How long ago [ ] [ ]  Fees: ________  Were you satisfied with  the service? [ ] [ ] | Where did you go [ ] [ ]  What did you go for? [ ] [ ]  How long ago [ ] [ ]  Fees: ________  Were you satisfied with  the service? [ ] [ ] | Where did you go [ ] [ ]  What did you go for? [ ] [ ]  How long ago [ ] [ ]  Fees: ________  Were you satisfied with  the service? [ ] [ ] |
| 01. Contraception  02. FP advice  03. ANC  04. PNC  05. VCT  06. STI test / treatment  07. HIV care  08. Other |  |  |  |  |  |
| ***When attended*** | Where did you go [ ] [ ]  What did you go for? [ ] [ ]  How long ago [ ] [ ]  Fees: ________  Were you satisfied with  the service? [ ] [ ] | Where did you go [ ] [ ]  What did you go for? [ ] [ ]  How long ago [ ] [ ]  Fees: ________  Were you satisfied with  the service? [ ] [ ] | Where did you go [ ] [ ]  What did you go for? [ ] [ ]  How long ago [ ] [ ]  Fees: ________  Were you satisfied with  the service? [ ] [ ] | Where did you go [ ] [ ]  What did you go for? [ ] [ ]  How long ago [ ] [ ]  Fees: ________  Were you satisfied with  the service? [ ] [ ] | Where did you go [ ] [ ]  What did you go for? [ ] [ ]  How long ago [ ] [ ]  Fees: ________  Were you satisfied with  the service? [ ] [ ] |
| 01. Last week  02. Last month  03. Last 3-6 months  04. Last year  06. More than a year ago  07. Can’t remember |  |  |  |  |  |
| ***Satisfaction*** | Where did you go [ ] [ ]  What did you go for? [ ] [ ]  How long ago [ ] [ ]  Fees: ________  Were you satisfied with  the service? [ ] [ ] | Where did you go [ ] [ ]  What did you go for? [ ] [ ]  How long ago [ ] [ ]  Fees: ________  Were you satisfied with  the service? [ ] [ ] | Where did you go [ ] [ ]  What did you go for? [ ] [ ]  How long ago [ ] [ ]  Fees: ________  Were you satisfied with  the service? [ ] [ ] | Where did you go [ ] [ ]  What did you go for? [ ] [ ]  How long ago [ ] [ ]  Fees: ________  Were you satisfied with  the service? [ ] [ ] | Where did you go [ ] [ ]  What did you go for? [ ] [ ]  How long ago [ ] [ ]  Fees: ________  Were you satisfied with  the service? [ ] [ ] |
| 01. Very satisfied  02. Satisfied  03. Neither satisfied nor dissatisfied  04. Dissatisfied  05. Very dissatisfied |  |  |  |  |  |

**SECTION 5: SERVICE USE**

**Use a separate sheet for each service use entered in Section 4**

| **No.** | **Questions and filters** | **Coding categories** | | **Skip to** |
| --- | --- | --- | --- | --- |
| 5.1 | **INTERVIEWER** to indicate what type of service | a. Family Planning  b. Pregnancy Care  c. HIV Testing & Counselling  d. HIV Monitoring and Treatment  e. STI Care | 1  2  3  4  5 |  |
| 5.2 | The first time you went there, what were your reasons for going to this particular service?  ***Circle all that apply*** | Referred there by a nurse or doctor  Recommended by a friend or relative  My partner told me to go there  Cheap / affordable  No shortages of medicines  Was easy to get to / near by  Short waiting times  Opening hours  Friendly providers / good treatment by provider  Confidentiality and privacy  Possibility to receive other health care services at the same time  It is my family doctor / nurse  Saw advertising for it  Was passing by  Didn’t know of any other services  Others (specify)  Don’t know | 1  2  3  4  5  6  7  8  9  10  11  12  13  14  15  16 99 |  |
| 5.3 | Overall, how satisfied would you say you are with this service?  ***read choices and score in box as:***  ***1 Very satisfied***  ***2 Satisfied***  ***3 Neither satisfied nor dissatisfied***  ***4 Dissatisfied***  ***5 Very dissatisfied***  ***99 Don’t know*** | Overall service |  |  |
|  |  | Cost of services |  |  |
|  |  | Availability of drugs and supplies (e.g. contraceptive methods) |  |  |
|  |  | Possibility to receive other health care services at the same time |  |  |
|  |  | Easy to get to |  |  |
|  |  | Waiting times |  |  |
|  |  | Opening hours |  |  |
|  |  | Friendliness of providers |  |  |
|  |  | Confidentiality and privacy |  |  |
| 5.4 | Would you recommend this service to a friend or relative? | Yes  No  Not sure  Don’t know | 1  2  3  99 |  |
| 5.5 | Do you intend to go back to this service again? | Yes  No  Not sure | 1  2  3 |  |

| **For any service user (all components):** | | | | | | | |  |
| --- | --- | --- | --- | --- | --- | --- | --- | --- |
| 5.6 | I am going to read out a list of things people may consider when deciding to use these types of health services. Please can you tell me how important each is for YOU in choosing a health facility? Please tell me if they are not at all important, somewhat important, or very important.  ***Score in box as:***  ***1 Not important***  ***2 Somewhat important***  ***3 Very important***  ***99 Don’t know*** | | Cost of services | | |  |  | |
|  |  |  | Availability of drugs and supplies (e.g. contraceptive methods) | | |  |  |  |
|  |  |  | Possibility to receive other health care services at the same time | | |  |  |  |
|  |  |  | Easy to get to | | |  |  |  |
|  |  |  | Waiting times | | |  |  |  |
|  |  |  | Opening hours | | |  |  |  |
|  |  |  | Friendliness of providers | | |  |  |  |
|  |  |  | Confidentiality and privacy | | |  |  |  |
| 5.7 | Are there any reasons that we haven’t mentioned that you consider important when deciding to go to a clinic? | | No  Yes (specify)  Don’t know | | | 0  1  99 |  | |
| 5.8 | How comfortable do you feel going to a clinic that women also attend for sexual health matters?  ***Read scale*** | | Doesn’t bother me at all  Bothers me a little  Bothers me a lot  I wouldn’t go to that service  I already attend a service like that  Don’t know | | | 1  2  3  4  5  99 |  | |
| 5.9 | How comfortable would you feel going to a clinic that also treated patients who have HIV or other sexually transmitted diseases?  ***Read scale*** | | Doesn’t bother me at all  Bothers me a little  Bothers me a lot  I wouldn’t go to that service  I already attend a service like that  Don’t know | | | 1  2  3  4  5  99 |  | |
| 5.10 | Have you heard of the follow clinics: | | Clinic X [ ] No [ ] Yes | Clinic Y [ ] No [ ] Yes | Clinic Z [ ] No [ ] Yes |  |  | |
| 5.11 | Please can you tell me if you think the following services are available from *index clinics*?  ***Circle those that apply*** | FP / contraception  Condoms  ANC  PNC  Child Immunizations  Delivery care  STI care  HIV VCT  HIV treatment  Male circumcision  TB treatment | 1  2  3  4  5  6  7  8  9  10  11 | 1  2  3  4  5  6  7  8  9  10  11 | 1  2  3  4  5  6  7  8  9  10  11 |  |  | |

**SECTION 6: SERVICE PERCEPTIONS**

**Finally, I would like to ask your thoughts about these services.**

Please put a mark on the line below at the point that shows your thoughts about the following services and their characteristics:

Example: Poor quality of care / Good quality of care

0% means poor quality of care ; 50% means fair quality of care; 100% means very good quality of care

(Visual Analogue Scale)

0 10 20 _30 40 _50 60 70 80 _90 100

| **+** | Element 1  (e.g. *index* clinic) | Element 2 | Element 3 | Element 4 | Element 5 | Element 6 | **-** |
| --- | --- | --- | --- | --- | --- | --- | --- |
| Construct 1^[[17]](#footnote-17)^  (e.g. Good care) |  |  |  |  |  |  | Construct 1  (e.g. Poor care) |
| Construct 2 |  |  |  |  |  |  | Construct 2 |
| Construct 3 |  |  |  |  |  |  | Construct 3 |
| Construct 4 |  |  |  |  |  |  | Construct 4 |
| Construct 5 |  |  |  |  |  |  | Construct 5 |
| Construct 6 |  |  |  |  |  |  | Construct 6 |
| Construct 7 |  |  |  |  |  |  | Construct 7 |
| Construct 8 |  |  |  |  |  |  | Construct 8 |
| Construct 9 |  |  |  |  |  |  | Construct 9 |

**Thank you for taking part in this survey. Did you have any questions you wanted to ask me?**

# Appendix 8.7: Community Survey of Men

**CONFIDENTIAL**

**IPPF Research Project on the
Integration of Sexual and Reproductive Health Services**

**Kenya 2008-2012**

**Community Survey of Men**

**BASELINE QUESTIONNAIRE**

**CLUSTER NUMBER:**

**HOUSEHOLD NUMBER:**

**INTERVIEWER’S CODE:**

**DATE OF INTERVIEW (dd-mm-yy): --**

**TIME STARTED (hh-mm): :**

**TIME ENDED: :**

| **SECTION 1: BACKGROUND INFORMATION** | | | | |
| --- | --- | --- | --- | --- |
| **No.** | **Questions and filters** | **Coding categories** | | **Skip to** |
| 1.1 | What is your date of birth | [ ] [ ] [ ] [ ] 19 [ ] [ ]  D D M M Y Y  Don’t know | 99 |  |
| 1.2 | What is your current marital status? | Single  Married monogamous  Married polygamous  Living with a partner  Divorced / separated / widowed  Others | 1  2  3  4  5  6 |  |
| 1.3 | What is the highest level of school you attended?  (or are still attending if at school) | None  Primary 1 to 4  Primary 5 to 8  Secondary  College / tertiary  Others | 1  2  3  4  5  6 |  |
| 1.4 | What is your religion? | Christian (Protestant)  Christian (Roman Catholic)  Christian (Pentecostal)  Islam  Zionist  Traditional  None  Other (specify)_________________________________________ | 1  2  3  4  5  6  7  8 |  |
| 1.5 | How would you describe your present employment situation? | Unemployed, looking for work  Unemployed, not looking for work  Work in informal sector, not looking for permanent work  Sick / disabled and unable to work  Student  Self-employed - full time (40 hours or more per week)  Self-employed - part time (less than 40 hours per week)  Employed part time (if none of the above) (less than 40 hours per week)  Employed full time (40 hours or more per week)  Other specify)_________________________________________ | 1  2  3  4  5  6  7  8  9  10 |  |
| 1.6 | Do you have enough money to meet your basic needs for food, clothes, health care and accommodation? | Not at all  A little  Moderately  Mostly  Completely | 1  2  3  4  5 |  |
| 1.7 | Does your household own any of the following in working condition:  *Circle all those that apply* | Television  Video / DVD player  Radio  Telephone / Mobile phone  Refrigerator  Microwave  Bicycle  Motorbike / scooter  Car | 1  1  1  1  1  1  1  1  1 |  |

| **SECTION 2: FERTILITY PREFERENCE & HIV** | | | | |
| --- | --- | --- | --- | --- |
| *Now I would like to ask you some very private information. Please be assured that what you tell me will be kept completely confidential and your name and address does not appear in this questionnaire.* | | | | |
| 2.1 | Do you have one regular sexual partner or do you ever have casual partners? | One regular partner  Casual partners only  Regular partner and casual partners | 1  2  3 |  |
| 2.2 | Have you had sexual intercourse in the last 1 month? | No  Yes | 0  1 |  |
| 2.3 | When do you think you may have your [first or next] child? | Within the next year  After the next 1-2 years  After 3-5 years  After 5 years from now  When I complete schooling  When I get a job  When I get married  No more children  Not applicable (can’t have / don’t want children)  Don’t know | 1  2  3  4  5  6  7  8  9  99 |  |
| 2.4 | Have you ever had an HIV test? | No  Yes  Don’t know | 0  1  99 |  |
| 2.5 | Do you know your HIV status? | Don’t know status  HIV negative  HIV positive  Awaiting results | 1  2  3  4 | 2.7  2.8 |
| 2.6 | Are you receiving monitoring or treatment in a health service? | No  Yes | 0  1 | 2.8  2.8 |
| 2.7 | Would you ever consider having an HIV test? | No  Yes  Maybe / Don’t know | 0  1  99 |  |
| 2.8 | Would you like to know where to obtain information on getting an HIV test or HIV treatment? | Yes (*give information on services)*  Maybe  No | 1  2  3 |  |
| 2.9 | Do you know if your current partner has ever had an HIV test? | No  Yes  Don’t know | 0  1  99 |  |
| 2.10 | Do you know your partner’s HIV status? | Don’t know partner’s status  HIV negative  HIV positive  Awaiting results | 1  2  3  4 | Sec 3 |
| 2.11 | Do you think your partner should have an HIV test? | No  Yes  Maybe / Don’t know | 0  1  99 |  |

| *Use a separate sheet for each partner mentioned in 2.1* | | | | |
| --- | --- | --- | --- | --- |
| **SECTION 3: CURRENT CONTRACEPTIVE USE Partner number** [ ] [ ] | | | | |
| 3.1 | Partner type | Wife / cohabiting partner  Girlfriend (not living together)  Casual acquaintance  Sex worker  Other (specify)____________________________ | 1  2  3  4  5 |  |
| 3.2 | Who makes decisions about what contraception to use in this relationship? | Partner does  I do  We both do | 1  2  3 |  |
| 3.3 | Have you ever used anything or tried in any way to delay or avoid this partner getting pregnant?  ***Show card of methods*** | No  Yes  Don’t know | 0  1  99 | 3.6 |
| 3.4 | Are you or this partner *currently* doing something or using any method to avoid getting pregnant? | No  Yes  Don’t know | 0  1  99 | 3.6 |
| 3.5 | What are you or this partner using or doing to avoid getting pregnant?  (circle all that apply)  ***Show card*** | Pills  Injectables  Male Condoms  Female condoms  IUD  Implants  Female sterilization  Male sterilization (vasectomy)  Diaphragm  Foam/Jelly  Lactational amenorrhoea method  Rhythm method  Withdrawal  Other | 1  2  3  4  5  6  7  8  9  10  11  12  13  14 | 3.7 |
| 3.6 | What is the main reason that no method was used? | Casual partner, doesn’t care  Contraception is women’s business  Woman is menopausal / had hysterectomy  Couple infertile / subfertile  Woman was pregnant  Woman was amenorrheic  Woman was breastfeeding  Wanted (more) children  Respondent opposed  Woman opposed  Others opposed  Religious prohibition  Knows no method  Knows no source  Health concerns  Fear of side effects  Lack of access / too far  Costs too much  Inconvenient to use  Interferes with body’s normal processes  Other (specify)___________________________  Do not know | 1  2  3  4  5  6  7  8  9  10  11  12  13  14  15  16  17  18  19  20  21  99 |  |
| 3.7 | At the time of your last act of intercourse did you use a condom with this partner? | No  Yes  Don’t know | 0  1  99 |  |
| 3.8 | Would you like to know where to obtain information on delaying or avoiding your partner getting pregnant? | No  Yes *(give information on services)*  Don’t know | 0  1  99 |  |

| **SECTION 4: HEALTH SERVICE USE** | | | | | |
| --- | --- | --- | --- | --- | --- |
| I am going to read to you a list of health services or needs. Please can you tell us whether you or your partner has used this service, and if so, which facility did you attend for the service at any time in the last 1 year. | | | | | |
| **Have you ever been to the doctor, nurse or other person for:** | **4.1 Family Planning** | **4.2 ANC or PNC** | **4.3 VCT** | **4.4 HIV monitoring / ART** | **4.5 STI care** |
|  | [ ] Attended service 1  [ ] Did not attend 2  [ ] Partner attended 3  [ ] Both attended 4 | [ ] Attended service 1  [ ] Did not attend 2  [ ] Partner attended 3  [ ] Both attended 4 | [ ] Attended service 1  [ ] Did not attend 2  [ ] Partner attended 3  [ ] Both attended 4 | [ ] Attended service 1  [ ] Did not attend 2  [ ] Partner attended 3  [ ] Both attended 4 | [ ] Attended service 1  [ ] Did not attend 2  [ ] Partner attended 3  [ ] Both attended 4 |
| **If attended:** | | | | | |
| **CODING** | Where did you go [ ] [ ]  What did you go for? [ ] [ ]  How long ago [ ] [ ]  Fees: ________  Were you satisfied with  the service? [ ] [ ] | Where did you go [ ] [ ]  What did you go for? [ ] [ ]  How long ago [ ] [ ]  Fees: ________  Were you satisfied with  the service? [ ] [ ] | Where did you go [ ] [ ]  What did you go for? [ ] [ ]  How long ago [ ] [ ]  Fees: ________  Were you satisfied with  the service? [ ] [ ] | Where did you go [ ] [ ]  What did you go for? [ ] [ ]  How long ago [ ] [ ]  Fees: ________  Were you satisfied with  the service? [ ] [ ] | Where did you go [ ] [ ]  What did you go for? [ ] [ ]  How long ago [ ] [ ]  Fees: ________  Were you satisfied with  the service? [ ] [ ] |
| ***Where attended*** |  |  |  |  |  |
| 01. Clinic X  02. Clinic Y  03. Clinic Z  04. Can’t remember |  |  |  |  |  |
| ***What for*** | Where did you go [ ] [ ]  What did you go for? [ ] [ ]  How long ago [ ] [ ]  Fees: ________  Were you satisfied with  the service? [ ] [ ] | Where did you go [ ] [ ]  What did you go for? [ ] [ ]  How long ago [ ] [ ]  Fees: ________  Were you satisfied with  the service? [ ] [ ] | Where did you go [ ] [ ]  What did you go for? [ ] [ ]  How long ago [ ] [ ]  Fees: ________  Were you satisfied with  the service? [ ] [ ] | Where did you go [ ] [ ]  What did you go for? [ ] [ ]  How long ago [ ] [ ]  Fees: ________  Were you satisfied with  the service? [ ] [ ] | Where did you go [ ] [ ]  What did you go for? [ ] [ ]  How long ago [ ] [ ]  Fees: ________  Were you satisfied with  the service? [ ] [ ] |
| 01. Contraception  02. FP advice  03. ANC  04. PNC  05. VCT  06. STI test / treatment  07. HIV care  08. Other |  |  |  |  |  |
| ***When attended*** | Where did you go [ ] [ ]  What did you go for? [ ] [ ]  How long ago [ ] [ ]  Fees: ________  Were you satisfied with  the service? [ ] [ ] | Where did you go [ ] [ ]  What did you go for? [ ] [ ]  How long ago [ ] [ ]  Fees: ________  Were you satisfied with  the service? [ ] [ ] | Where did you go [ ] [ ]  What did you go for? [ ] [ ]  How long ago [ ] [ ]  Fees: ________  Were you satisfied with  the service? [ ] [ ] | Where did you go [ ] [ ]  What did you go for? [ ] [ ]  How long ago [ ] [ ]  Fees: ________  Were you satisfied with  the service? [ ] [ ] | Where did you go [ ] [ ]  What did you go for? [ ] [ ]  How long ago [ ] [ ]  Fees: ________  Were you satisfied with  the service? [ ] [ ] |
| 01. Last week  02. Last month  03. Last 3-6 months  04. Last year  06. More than a year ago  07. Can’t remember |  |  |  |  |  |
| ***Satisfaction*** | Where did you go [ ] [ ]  What did you go for? [ ] [ ]  How long ago [ ] [ ]  Fees: ________  Were you satisfied with  the service? [ ] [ ] | Where did you go [ ] [ ]  What did you go for? [ ] [ ]  How long ago [ ] [ ]  Fees: ________  Were you satisfied with  the service? [ ] [ ] | Where did you go [ ] [ ]  What did you go for? [ ] [ ]  How long ago [ ] [ ]  Fees: ________  Were you satisfied with  the service? [ ] [ ] | Where did you go [ ] [ ]  What did you go for? [ ] [ ]  How long ago [ ] [ ]  Fees: ________  Were you satisfied with  the service? [ ] [ ] | Where did you go [ ] [ ]  What did you go for? [ ] [ ]  How long ago [ ] [ ]  Fees: ________  Were you satisfied with  the service? [ ] [ ] |
| 01. Very satisfied  02. Satisfied  03. Neither satisfied nor dissatisfied  04. Dissatisfied  05. Very dissatisfied |  |  |  |  |  |

**SECTION 5: SERVICE USE**

**Use a separate sheet for each service use entered in Section 4**

| **No.** | **Questions and filters** | | | **Coding categories** | | | | | | **Skip to** | |  |  |
| --- | --- | --- | --- | --- | --- | --- | --- | --- | --- | --- | --- | --- | --- |
| 5.1 | **INTERVIEWER** to indicate what type of service | | | a. Family Planning  b. Pregnancy Care  c. HIV Testing & Counselling  d. HIV Monitoring and Treatment  e. STI Care | | | | 1  2  3  4  5 | |  | |  |  |
| 5.2 | The first time you went there, what were your reasons for going to this particular service?  ***Circle all that apply*** | | | Referred there by a nurse or doctor  Recommended by a friend or relative  My partner told me to go there  Cheap / affordable  No shortages of medicines  Was easy to get to / near by  Short waiting times  Opening hours  Friendly providers / good treatment by provider  Confidentiality and privacy  Possibility to receive other health care services at the same time  It is my family doctor / nurse  Saw advertising for it  Was passing by  Didn’t know of any other services  Others (specify)  Don’t know | | | | 1  2  3  4  5  6  7  8  9  10  11  12  13  14  15  16  99 | |  | |  |  |
| 5.3 | Overall, how satisfied would you say you are with this service?  ***read choices and score in box as:***  ***1 Very satisfied***  ***2 Satisfied***  ***3 Neither satisfied nor dissatisfied***  ***4 Dissatisfied***  ***5 Very dissatisfied***  ***99 Don’t know*** | | | Overall service | | | |  | |  | |  |  |
|  |  |  |  | Cost of services | | | |  | |  |  |  |  |
|  |  |  |  | Availability of drugs and supplies (e.g. contraceptive methods) | | | |  | |  |  |  |  |
|  |  |  |  | Possibility to receive other health care services at the same time | | | |  | |  |  |  |  |
|  |  |  |  | Easy to get to | | | |  | |  |  |  |  |
|  |  |  |  | Waiting times | | | |  | |  |  |  |  |
|  |  |  |  | Opening hours | | | |  | |  |  |  |  |
|  |  |  |  | Friendliness of providers | | | |  | |  |  |  |  |
|  |  |  |  | Confidentiality and privacy | | | |  | |  |  |  |  |
| 5.4 | Would you recommend this service to a friend or relative? | | | Yes  No  Not sure  Don’t know | | | | 1  2  3  99 | |  | |  |  |
| 5.5 | Do you intend to go back to this service again? | | | Yes  No  Not sure  Don’t know | | | | 1  2  3  99 | |  | |  |  |
| **For any service user (all components):** | | | | | | | | | | | | |  |
| 5.6 | | I am going to read out a list of things people may consider when deciding to use these types of health services. Please can you tell me how important each is for YOU in choosing a health facility? Please tell me if they are not at all important, somewhat important, or very important.  ***Score in box as:***  ***1 Not important***  ***2 Somewhat important***  ***3 Very important***  ***99 Don’t know*** | | | Cost of services | | | |  | |  | | |
|  |  |  |  |  | Availability of drugs and supplies (e.g. contraceptive methods) | | | |  | |  |  |  |
|  |  |  |  |  | Possibility to receive other health care services at the same time | | | |  | |  |  |  |
|  |  |  |  |  | Easy to get to | | | |  | |  |  |  |
|  |  |  |  |  | Waiting times | | | |  | |  |  |  |
|  |  |  |  |  | Opening hours | | | |  | |  |  |  |
|  |  |  |  |  | Friendliness of providers | | | |  | |  |  |  |
|  |  |  |  |  | Confidentiality and privacy | | | |  | |  |  |  |
| 5.7 | | Are there any reasons that we haven’t mentioned that you consider important when deciding to go to a clinic? | | | No  Yes (specify)  Don’t know | | | | 0  1  99 | |  | | |
| 5.8 | | How comfortable do you feel going to a clinic that women also attend for sexual health matters?  ***Read scale*** | | | Doesn’t bother me at all  Bothers me a little  Bothers me a lot  I wouldn’t go to that service  I already attend a service like that | | | | 1  2  3  4  5 | |  | | |
| 5.9 | | How comfortable would you feel going to a clinic that also treated patients who have HIV or other sexually transmitted diseases?  ***Read scale*** | | | Doesn’t bother me at all  Bothers me a little  Bothers me a lot  I wouldn’t go to that service  I already attend a service like that | | | | 1  2  3  4  5 | |  | | |
| 5.10 | | Have you heard of the follow clinics: | | | Clinic X [ ] No [ ] Yes | Clinic Y [ ] No [ ] Yes | Clinic Z [ ] No [ ] Yes | |  | |  | | |
| 5.11 | | Please can you tell me if you think the following services are available from *index clinics*?  ***Circle those that apply*** | FP / contraception  Condoms  ANC  PNC  Child Immunizations  Delivery care  STI care  HIV VCT  HIV treatment  Male circumcision  TB treatment | | 1  2  3  4  5  6  7  8  9  10  11 | 1  2  3  4  5  6  7  8  9  10  11 | 1  2  3  4  5  6  7  8  9  10  11 | |  | |  | | |

**SECTION 6: SERVICE PERCEPTIONS**

**Finally, I would like to ask your thoughts about these services.**

Please put a mark on the line below at the point that shows your thoughts about the following services and their characteristics:

Example: Poor quality of care / Good quality of care

0% means poor quality of care ; 50% means fair quality of care; 100% means very good quality of care

(Visual Analogue Scale)

0 10 20 _30 40 _50 60 70 80 _90 100

| **+** | Element 1  (e.g. *index* clinic) | Element 2 | Element 3 | Element 4 | Element 5 | Element 6 | **-** |
| --- | --- | --- | --- | --- | --- | --- | --- |
| Construct 1^[[18]](#footnote-18)^  (e.g. Good care) |  |  |  |  |  |  | Construct 1  (e.g. Poor care) |
| Construct 2 |  |  |  |  |  |  | Construct 2 |
| Construct 3 |  |  |  |  |  |  | Construct 3 |
| Construct 4 |  |  |  |  |  |  | Construct 4 |
| Construct 5 |  |  |  |  |  |  | Construct 5 |
| Construct 6 |  |  |  |  |  |  | Construct 6 |
| Construct 7 |  |  |  |  |  |  | Construct 7 |
| Construct 8 |  |  |  |  |  |  | Construct 8 |
| Construct 9 |  |  |  |  |  |  | Construct 9 |

**Thank you for taking part in this survey. Did you have any questions you wanted to ask me?**

# Appendix 8.8: Client in-depth interview guide

**CONFIDENTIAL**

**IPPF Research Project on the
Integration of Sexual and Reproductive Health Services**

**Kenya 2008-2012**

**Client in-depth interview guide**

**Study ID NUMBER:**

**Recruited from:**

**INTERVIEWER’S CODE:**

**DATE OF INTERVIEW (dd-mm-yy): --**

**TIME STARTED (hh-mm): :**

**TIME ENDED: :**

***Note:*** *This topic guides are semi-structured guides and therefore are indicative of the topic areas to be covered. The guide consists of open-ended questions and will not be followed rigidly. It should not be seen as a structured questionnaire.*

**Consent sought:** Y N

| A. Background information | | | |
| --- | --- | --- | --- |
| **No.** | **Questions and filters** | **Coding categories** | |
| 1 | Sex | [ ] Male  [ ] Female | 0  1 |
| 2 | What is your date of birth | [ ] [ ] [ ] [ ] 19 [ ] [ ]  D D M M Y Y  [ ] Don’t know | 1  99 |
| 3 | What is your current marital status? | [ ] Single  [ ] Married monogamous  [ ] Married polygamous  [ ] Living with a partner  [ ] Divorced / separated / widowed  [ ] Other | 1  2  3  4  5  6 |
| 4 | What is the highest level of school you attended?  (or are still attending if at school) | [ ] None  [ ] Primary 1 to 4  [ ] Primary 5 to 8  [ ] Secondary  [ ] College / tertiary  [ ] Other | 1  2  3  4  5  6 |
| 5 | What is your religion? | [ ] Christian (Protestant)  [ ] Christian (Roman Catholic)  [ ] Christian (Pentecostal)  [ ] Islam  [ ] Zionist  [ ] Traditional  [ ] None  [ ] Other (specify)_______________________________________ | 1  2  3  4  5  6  7  8 |
| 6 | How would you describe your present employment situation? | [ ] Unemployed, looking for work  [ ] Unemployed, not looking for work  [ ] Work in informal sector, not looking for permanent work  [ ] Sick / disabled and unable to work  [ ] Student  [ ] Self-employed - full time (40 hours or more per week)  [ ] Self-employed - part time (less than 40 hours per week)  [ ] Employed part time (if none of the above) (less than 40 hours per week)  [ ] Employed full time (40 hours or more per week)  [ ] Other specify)_______________________ | 1  2  3  4  5  6  7  8  9  10  11  12  13 |
| 7 | Do you have enough money to meet your basic needs for food, clothes, health care and accommodation? | [ ] Not at all  [ ] A little  [ ] Moderately  [ ] Mostly  [ ] Completely | 1  2  3  4  5 |
| 8 | Does your household own any of the following in working condition:  *Tick all those that apply* | [ ] Television  [ ] Video / DVD player  [ ] Radio  [ ] Telephone / Mobile phone  [ ] Refrigerator  [ ] Microwave  [ ] Bicycle  [ ] Motorbike / scooter  [ ] Car | 1  1  1  1  1  1  1  1  1 |

| B. Single-question initial subsession (SQUIN) | |
| --- | --- |
| Please tell me about your use of any health services over the past year related to HIV or other health issues and problems, and please discuss whether you feel these services meet your own needs. Please discuss all events and experiences that have been important to you personally related to this topic; begin wherever you want, I won’t interrupt, I’ll just take some notes afterwards. | |
| C. Second subsession: narrative follow-up | |
| Notes from SQUIN: Stick to the sequence of topics raised in SQUIN and the words used | |
| 1 |  |
| 2 |  |
| 3 |  |
| 4 |  |
| 5 |  |
| 6 |  |
| 7 |  |
| 8 |  |
| 9 |  |
| 10 |  |
| 11 |  |
| 12 |  |
| 13 |  |

| Further notes |
| --- |
| D. Third subsession: non-narrative material  (if not previously discussed) |
| **Motivations for health care use and selection / use of the particular service** |
| Please can you tell me why you chose to go to *[insert service name]*? *(probes: cost, distance, friends recommendations, been before (why?), quality of care)* |
| Had you been before? Do you usually go to this service for HIV care? |
| Which other clinics do you know that offer HIV care in [town]? Have you used any of these other clinics? Why did you switch to this clinic? |
| Have you used this clinic for other services? Which ones? Did you find it helpful to have the services at the same clinic? |
| Do you know what other health services (aside from HIV care) that you could receive at this clinic? |
| 11. What do you consider important when deciding where to go for:  *Probe: Overall service, cost, availability of drugs and supplies, possibility to receive other health care services at the same time, easy to get to, waiting times, opening hours, friendliness of providers, confidentiality and privacy*   - HIV care - HIV test - family planning advice or contraception - Testing or treatment for STI - Postnatal or antenatal care (women)      - Smear test (women) |
| **Attitudes towards integration** |
| When you go to the clinic, is it usually just for one problem or need? Or do you often have many issues to discuss? |
| Is it important for you to be able to access different services in one clinic? |
| If you have more than one problem, is it the same nurse/doctor who deals with these problems? Or do you have to see different nurses/doctors? How do you feel about that? |
| Have you ever been referred from this clinic to another service? Did you go to your referral appointment? |
| How comfortable do / would you feel going to a clinic that [other sex] also attend for sexual health matters?  *If not comfortable, why?* |
| **Communication / interaction with the provider** |
| Do you usually see the same nurse / doctor at the clinic? Would you prefer to see the same nurse / doctor? Why is that? |
| Do you feel that the nurses/doctors are able to deal with all your problems? Why? |
| Do you feel that the nurses/doctors ask you enough questions or spend enough time with you? |
| Do you feel able to talk to them openly about all your needs or problems? Why? Do you feel able to ask anything that you want? |
| Do you trust the nurse/doctor with information that you tell them? |
| Do you think the nurse/doctor does a good job in caring for your health? Why? |
| What things do they do well? |
| What things do they do badly? |
| **Attitudes towards integrating HIV and SRH** |
| Do you think PLWH are treated well at this clinic? |
| Are they treated better there than at other clinics? |
| Do you think it is better to have services for PLWH in a specialised clinic for HIV or in a more general health clinic? Why? |
| Does your nurse or doctor at the clinic address your needs for other sexual and reproductive health issues, for example family planning, STIs, pregnancy care, HIV prevention? |
| If referred, how do you feel about going to other services for these needs? Would you prefer to have the services all together? Or is it better to stay in a specialised service? |
| **Contraceptive and sexual health behaviour** |
| Can you tell me about the sexual relationship/s you are currently involved in and who makes decision-making about contraception / family planning? |
| Do you know where your partner might go for any of these services? Do you ever attend these services together? *Why? Why not? Is there anyone else you go with?* |
| (Besides your partner), is there anyone else who you speak to about contraception, sexual health, HIV and where to go if you need any of these services? Is there anywhere else you would get this information from?  *Probe: friends, relatives, media; why they use this source and not others* |
| Do you have one regular sexual partner or do you ever have casual partners? |
| Are you planning on having any more children in the future? |
| Can you tell me about the contraceptive method you are currently using and how you came to be on that method? Why are you using that particular method?  *If not using, why?* |
| Do you know about any other types of methods? |
| At the time of your last act of intercourse did you use a condom with this partner?  Where did you obtain this from? |
| 1. Can you tell me where the best place to go would be if you wanted:  - HIV care *Why would you choose to go there?* - HIV test *Why would you choose to go there?* - family planning advice or contraception *Why would you choose to go there?* - Testing or treatment for STI *Why would you choose to go there?* - Postnatal or antenatal care (women) *Why would you choose to go there?* - Smear test (women) *Why would you choose to go there?* |
| INTERVIEWERS OBSERVATION  Comments about the respondent/s:  Comments on specific questions: |

# Appendix 8.9: FP/RH Client in-depth interview guide

**CONFIDENTIAL**

**IPPF/GATES Research Project on the
Integration of Sexual and Reproductive Health Services**

**Kenya 2008-2012**

**FP/RH Client in-depth interview guide**

**Study ID NUMBER:**

**Recruited from:**

**INTERVIEWER’S CODE:**

**DATE OF INTERVIEW (dd-mm-yy): --**

**TIME STARTED (hh-mm): :**

**TIME ENDED: :**

***Note:*** *This topic guides are semi-structured guides and therefore are indicative of the topic areas to be covered. The guide consists of open-ended questions and will not be followed rigidly. It should not be seen as a structured questionnaire.*

**Consent sought:** Y N

| A. Background information | | | |
| --- | --- | --- | --- |
| **No.** | **Questions and filters** | **Coding categories** | |
| 1 | Sex | [ ] Male  [ ] Female | 0  1 |
| 2 | What is your date of birth | [ ] [ ] [ ] [ ] 19 [ ] [ ]  D D M M Y Y  [ ] Don’t know | 1  99 |
| 3 | What is your current marital status? | [ ] Single  [ ] Married monogamous  [ ] Married polygamous  [ ] Living with a partner  [ ] Divorced / separated / widowed  [ ] Other | 1  2  3  4  5  6 |
| 4 | What is the highest level of school you attended?  (or are still attending if at school) | [ ] None  [ ] Primary 1 to 4  [ ] Primary 5 to 8  [ ] Secondary  [ ] College / tertiary  [ ] Other | 1  2  3  4  5  6 |
| 5 | What is your religion? | [ ] Christian (Protestant)  [ ] Christian (Roman Catholic)  [ ] Christian (Pentecostal)  [ ] Islam  [ ] Traditional  [ ] None  [ ] Other (specify)_______________________________________ | 1  2  3  4  5  6  7 |
| 6 | How would you describe your present employment situation? | [ ] Unemployed, looking for work  [ ] Unemployed, not looking for work  [ ] Work in informal sector, not looking for permanent work  [ ] Sick / disabled and unable to work  [ ] Student  [ ] Self-employed - full time (40 hours or more per week)  [ ] Self-employed - part time (less than 40 hours per week)  [ ] Employed part time (if none of the above) (less than 40 hours per week)  [ ] Employed full time (40 hours or more per week)  [ ] Other specify)_______________________ | 1  2  3  4  5  6  7  8  9  10  11  12  13 |
| 7 | Do you have enough money to meet your basic needs for food, clothes, health care and accommodation? | [ ] Not at all  [ ] A little  [ ] Moderately  [ ] Mostly  [ ] Completely | 1  2  3  4  5 |
| 8 | Does your household own any of the following in working condition:  *Tick all those that apply* | [ ] Television  [ ] Video / DVD player  [ ] Radio  [ ] Telephone / Mobile phone  [ ] Refrigerator  [ ] Microwave  [ ] Bicycle  [ ] Motorbike / scooter  [ ] Car | 1  1  1  1  1  1  1  1  1 |

| B. Single-question initial subsession (SQUIN) | |
| --- | --- |
| Please tell me about your use of any health services over the past year related to sexual and reproductive health, and by this I mean family planning services or care for mothers and babies, as well as services  for sexually transmitted infectsions or HIV, and please discuss whether you feel these services meet your own needs. Please discuss all events and experiences that have been important to you personally related to this topic; begin wherever you want, I won’t interrupt, I’ll just take some notes afterwards. | |
| C. Second subsession: narrative follow-up | |
| Notes from SQUIN: Stick to the sequence of topics raised in SQUIN and the words used | |
| 1 |  |
| 2 |  |
| 3 |  |
| 4 |  |
| 5 |  |
| 6 |  |
| 7 |  |
| 8 |  |
| 9 |  |
| 10 |  |
| 11 |  |
| 12 |  |
| 13 |  |
| 14 |  |

| Further notes |
| --- |
| D. Third subsession: non-narrative material  (if not previously discussed) |
| **Motivations for health care use and selection / use of the particular service** |
| Please can you tell me why you chose to go to *[insert service name]*? *(probes: cost, distance, friends recommendations, been before (why?), quality of care)* |
| Had you been before? Do you usually go to this service for FP? |
| Which other clinics do you know that offer FP in [town]? Have you used any of these other clinics? Why did you switch to this clinic? |
| Have you used this clinic for other services? Which ones? Did you find it helpful to have the services at the same clinic? |
| Do you know what other health services (aside from FP) that you could receive at this clinic? |
| What do you consider important when deciding where to go for:  *Probe: Overall service, cost, availability of drugs and supplies, possibility to receive other health care services at the same time, easy to get to, waiting times, opening hours, friendliness of providers, confidentiality and privacy*   - family planning advice or contraception - Testing or treatment for STI - HIV test - Postnatal or antenatal care (women) - Smear test (women) |
| **Attitudes towards integration** |
| When you go to the clinic, is it usually just for one problem or need? Or do you often have many issues to discuss? |
| Is it important for you to be able to access different services in one clinic? |
| If you have more than one problem, is it the same nurse/doctor who deals with these problems? Or do you have to see different nurses/doctors? How do you feel about that? |
| Have you ever been referred from this clinic to another service? Did you go to your referral appointment? |
| How comfortable do / would you feel going to a clinic that [other sex] also attend for sexual health matters?  *If not comfortable, why?* |
| **Communication / interaction with the provider** |
| Do you usually see the same nurse / doctor at the clinic? Would you prefer to see the same nurse/doctor? Why is that? |
| Do you feel that the nurses / doctors are able to deal with all your problems? Why? |
| Do you feel that the nurses/doctors ask you enough questions or spend enough time with you? |
| Do you feel able to talk to them openly about all your needs or problems? Why? Do you feel able to ask anything that you want? |
| Do you trust the nurse/doctor with information that you tell them? |
| Do you think the nurse/doctor does a good job in caring for your health? Why? |
| What things do they do well? |
| What things do they do badly |
| **Attitudes towards integrating HIV and SRH** |
| How do you feel about having HIV services provided at your clinic? |
| Do you think PLWH are treated well at this clinic? |
| Are they treated better there than at other clinics? |
| Do you think it is better to have services for PLWH in your clinic, or in a specialised clinic on HIV? Why? |
| **Contraceptive and sexual health behaviour** |
| Can you tell me about the sexual relationship/s you are currently involved in and who makes decision-making about contraception / family planning? |
| Do you know where your partner might go for any of these services? Do you ever attend these services together? *Why? Why not? Is there anyone else you go with?* |
| (Besides your partner), is there anyone else who you speak to about contraception, sexual health, HIV and where to go if you need any of these services? Is there anywhere else you would get this information from?  *Probe: friends, relatives, media; why they use this source and not others* |
| Do you have one regular sexual partner or do you ever have casual partners? |
| Are you planning on having any more children in the future? |
| Can you tell me about the contraceptive method you are currently using and how you came to be on that method? Why are you using that particular method?  *If not using, why?* |
| Do you know about any other types of methods? |
| At the time of your last act of intercourse did you use a condom with this partner?  Where did you obtain this from? |
| 1. Can you tell me where the best place to go would be if you wanted:  - family planning advice or contraception *Why would you choose to go there?* - Testing or treatment for STI *Why would you choose to go there?* - HIV test *Why would you choose to go there?* - Postnatal or antenatal care (women) *Why would you choose to go there?* - Smear test (women) *Why would you choose to go there?* |
| Have you ever had an HIV test?  *If yes discuss experience of deciding where to go and how the process was.*  *If no, discuss whether interviewee would ever consider this and how the type of health facility or provider would affect their choice.* |
| INTERVIEWERS OBSERVATION  Comments about the respondent/s:  Comments on specific questions: |

# Appendix 8.10 FP/RH Provider in-depth interview guide

**CONFIDENTIAL**

**IPPF/Gates Research Project on the
Integration of Sexual and Reproductive Health Services**

**Kenya 2008-2012**

**FP/RH Provider in-depth interview guide**

**Study ID NUMBER:**

**Recruited from:**

**INTERVIEWER’S CODE:**

**DATE OF INTERVIEW (dd-mm-yy): --**

**TIME STARTED (hh-mm): :**

**TIME ENDED: :**

***Note:*** *This topic guides are semi-structured guides and therefore are indicative of the topic areas to be covered. The guide consists of open-ended questions and will not be followed rigidly. It should not be seen as a structured questionnaire.*

**Consent sought:** Y N

| A. Background information | | | |
| --- | --- | --- | --- |
| **No.** | **Questions and filters** | **Coding categories** | |
| 1 | Sex | [ ] Male  [ ] Female | 0  1 |
| 2 | What is your date of birth | [ ] [ ] [ ] [ ] 19 [ ] [ ]  D D M M Y Y  [ ] Don’t know | 1  99 |
| 3 | What is your current position in this health service? | [ ] Nurse  [ ] Doctor  [ ] Counsellor  [ ] Manager  [ ] Other (specify)_______________________________________ | 1  2  3  4  5 |
| 4 | How long have you been working here? |  |  |
| 5 | Have you worked anywhere else? |  |  |
| 6 | What is your religion? | [ ] Christian (Protestant)  [ ] Christian (Roman Catholic)  [ ] Christian (Pentecostal)  [ ] Islam  [ ] Zionist  [ ] Traditional  [ ] None  [ ] Other (specify)_______________________________________ | 1  2  3  4  5  6  7  8 |

| B. Single-question initial subsession (SQUIN) | |
| --- | --- |
| Please tell me about your role as a [health care provider] in your clinic, and whether the services you offer meet the needs of the community? Please discuss all events and experiences that have been important to you personally related to this topic; begin wherever you want, I won’t interrupt, I’ll just take some notes afterwards. | |
| C. Second subsession: narrative follow-up | |
| Notes from SQUIN: Stick to the sequence of topics raised in SQUIN and the words used | |
| 1 |  |
| 2 |  |
| 3 |  |
| 4 |  |
| 5 |  |
| 6 |  |
| 7 |  |
| 8 |  |
| 9 |  |
| 10 |  |

| Further notes: |
| --- |
| D. Third subsession: non-narrative material  (if not previously discussed) |
| **Services offered** |
| What type of services are offered at your clinic? |
| What specific type of healthcare do you provide? Have you always had this focus? |
| How did you start working in this area? |
| **Attitudes towards integration** |
| Have you heard of the concept of integrating sexual and reproductive health care with HIV care? |
| What does integrated care mean to you? |
| Do you consider yourself a generalist or a specialist? Why? |
| Why did you decide to be a generalist / specialist? |
| How do you feel about providing integrated care? |
| Do you feel you have enough training to provide a range of SRH care? |
| Which services do you feel you require more training in? |
| **Practicalities of integrating** |
| Do you practice integrated care? |
| How many of your clients typically come with more than one health care need? |
| What additional topics of health care needs may be addressed during a typical FP consultation? |
| **Benefits and challenges** |
| What do you see as some of the benefits to integrating sexual and reproductive health and HIV care? *(probes: meeting clients needs/satisfaction, prevention, holism, efficiency, continuity, stigma, reaching men / youth)* |
| What are some of the challenges? *(probes: time, waiting times, capacity, guidance / guidelines, attitudes, supervision, complexity, referrals, loss of specialisation, separate funding/management)* |
| **Clients** |
| Do you feel your clients want integrated care? |
| Do they request or demand it? Or do you need to push to cover more health care topics? |
| Do they have to pay more for integrated care? |
| **Referrals** |
| What kind of services are clients referred for? |
| Are they referred internally or externally? How often? |
| Do you think referrals are successful? |
| Do you think care should be integrated at the ‘provider’ level, or at the ‘facility’ level? Does it make a difference? |
| **Integrating HIV care and attitudes towards PLWH** |
| How long has this service been offering HIV care? |
| Do you feel this is an important component of your service? |
| How is HIV care provided? |
| Do you feel that care for HIV patients is integrated with other SRH services? |
| How are the SRH needs of HIV patients being met? |
| Do you feel comfortable providing services to PLWH? |
| How do you think other SRH clients feel about this service offering HIV services? |
| **Other healthcare needs** |
| Do you think that clients have any other health care needs that are not being addressed through your services? |
| What kind of issues do you feel unable or not confident to address? How do you deal with this? |
| What about PLWH? Do they have additional health care needs? |
| INTERVIEWERS OBSERVATION  Comments about the respondent/s  Comments on specific questions: |

# Appendix 8.11: HIV Provider in-depth interview guide

**IPPF/GATES Research Project on the
Integration of Sexual and Reproductive Health Services**

**Kenya 2008-2012**

**HIV Provider in-depth interview guide**

**Study ID NUMBER:**

**Recruited from:**

**INTERVIEWER’S CODE:**

**DATE OF INTERVIEW (dd-mm-yy): --**

**TIME STARTED (hh-mm): :**

**TIME ENDED: :**

***Note:*** *This topic guides are semi-structured guides and therefore are indicative of the topic areas to be covered. The guide consists of open-ended questions and will not be followed rigidly. It should not be seen as a structured questionnaire.*

**Consent sought:** Y N

| A. Background information | | | |
| --- | --- | --- | --- |
| **No.** | **Questions and filters** | **Coding categories** | |
| 1 | Sex | [ ] Male  [ ] Female | 0  1 |
| 2 | What is your date of birth | [ ] [ ] [ ] [ ] 19 [ ] [ ]  D D M M Y Y  [ ] Don’t know | 1  99 |
| 3 | What is your current position in this health service? | [ ] Nurse  [ ] Doctor  [ ] Counsellor  [ ] Manager  [ ] Other (specify)_______________________________________ | 1  2  3  4  5 |
| 4 | How long have you been working here? |  |  |
| 5 | Have you worked anywhere else? |  |  |
| 6 | What is your religion? | [ ] Christian (Protestant)  [ ] Christian (Roman Catholic)  [ ] Christian (Pentecostal)  [ ] Islam  [ ] Traditional  [ ] None  [ ] Other (specify)_______________________________________ | 1  2  3  4  5  6  7 |

| B. Single-question initial subsession (SQUIN) | |
| --- | --- |
| Please tell me about your role as a [health care provider] in your clinic, and whether the services you offer meet the needs of the community? Please discuss all events and experiences that have been important to you personally related to this topic; begin wherever you want, I won’t interrupt, I’ll just take some notes afterwards. | |
| C. Second subsession: narrative follow-up | |
| Notes from SQUIN: Stick to the sequence of topics raised in SQUIN and the words used | |
| 1 |  |
| 2 |  |
| 3 |  |
| 4 |  |
| 5 |  |
| 6 |  |
| 7 |  |
| 8 |  |
| 9 |  |
| 10 |  |

| Further notes |
| --- |
| D. Third subsession: non-narrative material  (if not previously discussed) |
| **Services offered** |
| What type of services are offered at your clinic? |
| What specific type of healthcare do you provide? Have you always had this focus? |
| How did you start working in this area? |
| **Attitudes towards integration** |
| Have you heard of the concept of integrating sexual and reproductive health care with HIV care? |
| What does integrated care mean to you? |
| Do you consider yourself a generalist or a specialist? Why? |
| Why did you decide to be a generalist / specialist? |
| How do you feel about providing integrated care? |
| Do you feel you have enough training to provide a range of HIV and SRH care? |
| Which services do you feel you require more training in? |
| **Integrating HIV care and attitudes towards PLWH** |
| How long has this service been offering HIV care? |
| Do you integrate care SRH care with HIV care?  *If so, how is this done? If not, why is this not done?* |
| Do you feel that many of your HIV clients have a need for SRH care? What kind of needs do they have? |
| Do you feel this is an important component of your service? *(integrated only)* |
| Do you feel comfortable providing services to PLWH? |
| How do you think other SRH clients feel about this service offering HIV services? *(where applicable)* |
| **Benefits and challenges** |
| What do you see as some of the benefits to integrating sexual and reproductive health and HIV care? *(probes: meeting clients needs/satisfaction, prevention, holism, efficiency, continuity, stigma, reaching men / youth)* |
| What are some of the challenges? *(probes: time, waiting times, capacity, guidance / guidelines, attitudes, supervision, complexity, referrals, loss of specialisation, separate funding/management)* |
| **Clients** |
| Do you feel your clients want integrated care? |
| Do they request or demand it? Or do you need to push to cover more health care topics? |
| Do they have to pay more for integrated care? |
| **Referrals** |
| What kind of services are clients referred for? |
| Are they referred internally or externally? How often? |
| Do you think referrals are successful? |
| Do you think care should be integrated at the ‘provider’ level, or at the ‘facility’ level? Does it make a difference? |
| **Integrating HIV care and attitudes towards PLWH** |
| How long has this service been offering HIV care? |
| Do you feel this is an important component of your service? |
| How is HIV care provided? |
| Do you feel that care for HIV patients is integrated with other SRH services? |
| How are the SRH needs of HIV patients being met? |
| Do you feel comfortable providing services to PLWH? |
| How do you think other SRH clients feel about this service offering HIV services? |
| **Other healthcare needs** |
| Do you think that clients have any other health care needs that are not being addressed through your services? |
| What kind of issues do you feel unable or not confident to address? How do you deal with this? |
| What about PLWH? Do they have additional health care needs? |
| INTERVIEWERS OBSERVATION  Comments about the respondent/s  Comments on specific questions: |

# Appendix 8.12: Economics component periodic activity review tool

**Economic Analysis of Integrated SRH and HIV Services in Kenya**

**Periodic Activity Review of Facilities providing Integrated SRH & HIV Services**

**Topic Guide for Information Collection**

**London School of Hygiene and Tropical Medicine**

**Economics Team –Kumaranayake, Obure, Terris-Prestholt, Watts**

The periodic activity review has the following objectives:

A. To understand the organization and size of the facility.

B. To review activities and services currently being delivered in each facility.

1. To understand the evolution of services i.e. was a service previously provided? How long has the service been delivered for?
2. To identify new services or activities planned and the likely timeframe.
3. To understand what other facilities/services patients are referred to?

C. To understand how integration of services provided in this facility works.

D. To understand the patient flow, by illustrating what happens when a patient comes into a clinic.

1. To provide patient information - Are the patients accessing more than one service and how is the service flow registered?

E. To understand the nature of existing monitoring.

1. What indicators are collected and how are they collected? E.g. For age, are the actual ages noted down or is a patient labeled as either youth or adult? What information is actually entered and who enters the information? Is it the patient or the service providers? What is collected in paper or electronic form?
2. To match indicators with services.

F. To identify providers of substitute services if any in the community.

G. To feed into the development of a costing protocol.

This instrument is designed for members of the economics team to collect information speaking to key staff in the facilities and through observation of activities.

| **A. Facility Description** | | | | | | | | |
| --- | --- | --- | --- | --- | --- | --- | --- | --- |
| A1. Name of facility/location | | |  | | | | | |
| A2. Contact Person | | | | |  | | | |
| A3. When was the facility established? | | | | |  | | | |
| A4. Brief description of how the facility is organized or draw organogram. If available get a copy of an organization chart if any. | | | | | | | | |
|  | | | | | | | | |
| A5. Facility operating hours | | | | | | | | |
| Mon | Tues | Wed | | Thur | | Fri | Sat | Sun |
|  |  |  | |  | |  |  |  |

| A6. Facility size (number of rooms available). A separate sheet is included to draw a map of the rooms in the facility. | | | | | | | | | | | | |
| --- | --- | --- | --- | --- | --- | --- | --- | --- | --- | --- | --- | --- |
| 1. Waiting rooms | |  | 4. VCT rooms | | |  | | 7. | | |  | |
| 2. Consultation rooms | |  | 5. Other | | |  | | 8. | | |  | |
| 3. Lab rooms | |  | 6. | | |  | | 9. | | |  | |
| A7. What is the total number of staff currently working in the facility? | | | | | | | | | | | | |
| Full Time: |  | | | Part-Time: |  | | | | Volunteers |  | | |
| Types of Staff (Please Provide #s) | | | | | | | | | | | | |
| Doctors (D) | |  | Pharmacists (P) | | | |  | | Administrative staff (AS) | | |  |
| Nurses (N) | |  | Lab technicians (LTs) | | | |  | | Support staff (SS) | | |  |
| Midwives (M) | |  | Counselors (C) | | | |  | |  | | |  |
| Gynecologists/  Obstetricians (OBGYN) | |  | Lay counselors (LC) | | | |  | |  | | |  |
| Anesthetists (A) | |  | Community Outreach Workers (COW) | | | |  | |  | | |  |

| A8. Services offered at the facility. A description of all services offered at the facility, including SRH and HIV Services and target groups for each service. | |
| --- | --- |
| **Service** | **Target population** |
|  |  |
|  |  |
|  |  |
|  |  |
|  |  |
|  |  |
|  |  |

**Map of the Facilities Map of the Facilities –** This is a sketch of the physical layout of the facilities. Numbers refer to category of room from question A6.

| **B. Overall Description Of SRH And HIV Related Activities Offered** | | | | |
| --- | --- | --- | --- | --- |
| B1. Does your facility offer the following integrated sexual and reproductive health products and services? If yes, how long has this service been offered? (Start date). For services offered, please also indicate what type of staff provides the service (i.e. doctor, nurse, lab tech). | | | | |
|  | **Yes** | **No** | **If Yes,**  **Start Date** | **Staff type involved in service provision** |
| Contraceptive pills |  |  |  |  |
| Intra-uterine contraceptive device |  |  |  |  |
| Injectables |  |  |  |  |
| Implants |  |  |  |  |
| Vasectomy and bilateral tubal ligation |  |  |  |  |
| Male and female condoms |  |  |  |  |
| Post partum FP |  |  |  |  |
| ANC and PNC |  |  |  |  |
| Voluntary counseling and testing |  |  |  |  |
| Anti retroviral treatment |  |  |  |  |
| STI management |  |  |  |  |
| Male circumcision |  |  |  |  |
| Maternity services/gyne services |  |  |  |  |
| Child health services (immunization & routine checkups) |  |  |  |  |
| Pap smear / cervical cancer screening |  |  |  |  |
| Emergency contraception |  |  |  |  |
| Post abortion care & manual vacuum aspiration |  |  |  |  |
| Unwanted/ Teenage Pregnancy Counselling |  |  |  |  |
| Management of infertility |  |  |  |  |
| Education on adolescent sexuality |  |  |  |  |
| General FP counseling |  |  |  |  |
| Youth friendly services |  |  |  |  |
| Out client services |  |  |  |  |
| Pharmacy |  |  |  |  |

| **B2. Are there any other services or activities other than those indicated above, offered in the facility? Please list and include date service began.** | | | | |
| --- | --- | --- | --- | --- |
| **Service** | **Start Date** | | | **Staff type involved in service provision** |
|  |  | | |  |
|  |  | | |  |
|  |  | | |  |
|  |  | | |  |
|  |  | | |  |
| **B3. Are there any new services/activities that are yet to be implemented? If yes, Please provide details including the likely timeframe.** | | | | |
| **Service** | **Expected start date** | | | |
|  |  | | | |
|  |  | | | |
|  |  | | | |
|  |  | | | |
|  |  | | | |
|  |  | | | |
|  |  | | | |
| **B4. For services not offered in this facility from question B1, where are clients referred to?** | | | | |
| **Service** | | **Referral Location** | | |
|  | | |  | |
|  | | |  | |
|  | | |  | |
|  | | |  | |
|  | | |  | |
|  | | |  | |
|  | | |  | |

| **B5. Service Provision Hours** | | | | | | |
| --- | --- | --- | --- | --- | --- | --- |
|  | **Mon** | **Tue** | **Wed** | **Thur** | **Fri** | **Sat** |
| Contraceptive pills |  |  |  |  |  |  |
| Intra-uterine contraceptive device |  |  |  |  |  |  |
| Injectables |  |  |  |  |  |  |
| Implants |  |  |  |  |  |  |
| Vasectomy and bilateral tubal ligation |  |  |  |  |  |  |
| Male and female condoms |  |  |  |  |  |  |
| Post partum FP |  |  |  |  |  |  |
| ANC and PNC |  |  |  |  |  |  |
| Voluntary counseling and testing |  |  |  |  |  |  |
| Anti retroviral treatment |  |  |  |  |  |  |
| STI management |  |  |  |  |  |  |
| Male circumcision |  |  |  |  |  |  |
| Maternity services/gynecological services |  |  |  |  |  |  |
| Child health services (immunization & routine checkups) |  |  |  |  |  |  |
| Pap smear for cervical cancer screening |  |  |  |  |  |  |
| Emergency contraception |  |  |  |  |  |  |
| Post abortion care |  |  |  |  |  |  |
| Unwanted/teenage pregnancy counseling |  |  |  |  |  |  |

|  | **Mon** | **Tue** | **Wed** | **Thur** | **Fri** | **Sat** |
| --- | --- | --- | --- | --- | --- | --- |
| Management of infertility |  |  |  |  |  |  |
| Education on adolescent sexuality |  |  |  |  |  |  |
| General FP counseling |  |  |  |  |  |  |
| Youth friendly services |  |  |  |  |  |  |
| Outclient |  |  |  |  |  |  |
| Pharmacy |  |  |  |  |  |  |
|  |  |  |  |  |  |  |
|  |  |  |  |  |  |  |
|  |  |  |  |  |  |  |
|  |  |  |  |  |  |  |
|  |  |  |  |  |  |  |
|  |  |  |  |  |  |  |
|  |  |  |  |  |  |  |
|  |  |  |  |  |  |  |
|  |  |  |  |  |  |  |

| **C. How do services work together?** |
| --- |
| C1. Describe the type of integration currently in the facility. i.e. is it structural or functional. Structural integration is defined as provision of different services by different people under the same roof while functional Integration is defined as provision of different services by the same person. |
|  |

| C2. Has the type of integration in the facility always been like this? | | Yes |  | No | |  |
| --- | --- | --- | --- | --- | --- | --- |
| If No, when did the change occur? | Last Year | | | |  | |
|  | 2 Years ago | | | |  | |
|  | 3 Years ago | | | |  | |
| Details of how integration has changed over the last 3 years. | | | | | | |
|  | | | | | | |
| C3. When did service integration begin and how was it initiated? | | | | | | |
|  | | | | | | |

| C4. Was there any training on service integration provided to staff? | Yes |  | No |  |
| --- | --- | --- | --- | --- |
| If Yes, provide a summary of the training provided | | | | |
|  | | | | |

| **D. Client Flow** |
| --- |
| D1. Please describe a typical client visit. i.e. Who does the client see upon arrival at the clinic and then what happens. E.g. Someone coming in for FP who is not pregnant and doesn’t want to get pregnant. Attach room numbers etc. How many doors do they need to go through |
|  |
|  |
|  |
|  |
|  |
|  |
|  |
|  |
|  |
|  |

| D2. A pictorial representation of patient flow |
| --- |
| Based on the map of the clinic, show the different sequential locations that the patient visits. |

| **E. Monitoring And Evaluation** | | | | | |
| --- | --- | --- | --- | --- | --- |
| E1 Are records kept of the number of clients using each service? | Yes |  | | No |  |
| If yes in what form are the records kept? | Electronic |  | | Paper |  |
| If registers are used, how many registers are used to collect the information required? Do the registers have titles or are they by service. | | | | | |
| **Type of Register** | **# of Registers** | | **Who collects information?** | | |
|  |  | |  | | |
|  |  | |  | | |
|  |  | |  | | |
|  |  | |  | | |
|  |  | |  | | |
|  |  | |  | | |
|  |  | |  | | |
|  |  | |  | | |
| E2 What types of routine monitoring data is collected? Please check those that apply. | | | | | |
| Client Numbers by age and sex | | | |  | |
| Location of clients residence | | | |  | |
| Services Accessed by client for each visit | | | |  | |
| Referrals to other services made by client | | | |  | |
|  | | | |  | |
|  | | | |  | |
|  | | | |  | |

| **E3. Indicators for each service provided. Please provide process indicators for monitoring integrated services provided in the facility (Table to be filled out looking at existing records and registers)** | | |
| --- | --- | --- |
| **Service** | **Indicators** | **How long have indicators been collected for (indicate start year):** |
| Contraceptive Pills |  |  |
|  |  |  |
| Intra-Uterine Contraceptive Device |  |  |
|  |  |  |
| Injectables |  |  |
|  |  |  |
|  |  |  |
| Implants |  |  |
|  |  |  |
| Vasectomy and bilateral tubal ligation |  |  |
|  |  |  |
| Male and Female condoms |  |  |
|  |  |  |
| Post partum FP |  |  |
|  |  |  |
| ANC and PNC |  |  |
|  |  |  |
| Voluntary counseling and testing |  |  |
|  |  |  |
| Anti retroviral treatment |  |  |
|  |  |  |
| STI management |  |  |
|  |  |  |
| Male circumcision |  |  |
|  |  |  |
| Maternity Services & Gynecological services |  |  |
|  |  |  |
| Child health services (immunization & routine checkups) |  |  |
|  |  |  |
| Pap smear for cervical cancer screening |  |  |
|  |  |  |
| Emergency contraception |  |  |
|  |  |  |

| **Services** | **Indicators** | **How long have indicators been collected for (indicate year):** | |
| --- | --- | --- | --- |
| Post abortion care |  |  | |
|  |  |  | |
| Unwanted/ teenage pregnancy counseling |  |  |  |
|  |  |  |  |
| Management of infertility |  |  |  |
|  |  |  |  |
| Education on adolescent sexuality |  |  |  |
|  |  |  |  |
|  |  |  |  |
| General FP counseling |  |  |  |
|  |  |  |  |
| Youth friendly services |  |  |  |
|  |  |  |  |
| Outpatient Client |  |  |  |
|  |  |  |  |
| Pharmacy |  |  |  |
|  |  |  |  |

| E4. How often are summary reports prepared? | | | | | | | | | | | | | | |
| --- | --- | --- | --- | --- | --- | --- | --- | --- | --- | --- | --- | --- | --- | --- |
| Weekly  (W) |  | Bi-Weekly (BW) |  | | Monthly (M) |  | Quarterly (Q) | | |  | Yearly  (Y) | | |  |
| Do these summary reports include the indicators in E3? | | | | | | | | Yes | |  | | No |  | |
| If No, what do these summary reports include? | | | | | | | | | | | | | | |
|  | | | | | | | | | | | | | | |
| **F. Provider Mapping. Substitute Services – Who else provides similar types of products and services within the community?** | | | | | | | | | | | | | | |
| **Service** | | | | **Service Provider** | | | | | **Location** | | | | | |
|  | | | |  | | | | |  | | | | | |
|  | | | |  | | | | |  | | | | | |
|  | | | |  | | | | |  | | | | | |
|  | | | |  | | | | |  | | | | | |
|  | | | |  | | | | |  | | | | | |
|  | | | |  | | | | |  | | | | | |

**Notes – General Observations (on additional pages)**

**List of People (positions and contact details) talked to.**

| **Contact Name** | **Position** | **Contact Details** |
| --- | --- | --- |
|  |  |  |
|  |  |  |
|  |  |  |
|  |  |  |
|  |  |  |
|  |  |  |
|  |  |  |
|  |  |  |
|  |  |  |

1. August 2005 UNAIDS policy position paper Intensifying HIV Prevention and the UNGASS Review, June 2006. These build upon both the New York Call for Commitment: Linking HIV/AIDS and SRH and the Glion Call to Action on FP and HIV/AIDS in Women and Children. [↑](#footnote-ref-1)
2. ‘Maputo Plan of Action for the operationalization of the continental policy framework for sexual and reproductive health and rights, 2007-2010’ Report from the Special Session of the African Union, Conference of Ministers of Health, Maputo Mozambique, 18-22 September 2006 [↑](#footnote-ref-2)
3. WHO/UNFPA/UNAIDS/IPPF, Sexual and Reproductive Health and HIV/AIDS, A Framework for Priority Linkages, 2006 (in <http://content.ippf.org/output/ORG/files/12345.pdf>) [↑](#footnote-ref-3)
4. Berhane, Y. Linking Reproductive Health, Family Planning and HIV/AIDS in Africa, Closing remarks, Ethiopia, 2006. [↑](#footnote-ref-4)
5. Liambila, W. et al. 2008. *“Feasibility, acceptability, effect and cost of integrating counseling and testing for HIV within family planning services in Kenya,”* FRONTIERS Final Report. Washington, DC: Population Council. [↑](#footnote-ref-5)
6. Sweat, M. et al. 2000 “Cost-effectiveness of voluntary HIV-1 counselling and testing in reducing sexual transmission of HIV-1 in Kenya and Tanzania,” *The Lancet* 356:113-21. [↑](#footnote-ref-6)
7. Another protocol is being submitted simultaneously to the Population Council IRB by the same consortium, which is addressing the integration of HIV with postpartum RH services in Kenya and Swaziland. [↑](#footnote-ref-7)
8. The IPPF affiliate in Kenya is Family Health Options of Kenya (FHOK). [↑](#footnote-ref-8)
9. Liambila et al 2008. [↑](#footnote-ref-9)
10. Wengraf, T. (2001) Qualitative Research Interviewing Biographic Narrative and Semi-Structured Methods. London: Sage Publications. [↑](#footnote-ref-10)
11. The term ‘Health Facility Assessment’ is used to describe methodologies for assessing the readiness of facilities to offer services and the quality of care provided. For an overview of a range of methods, see HFA TWG. 2007. Profiles of Health Facility Assessment Methods, MEASURE Evaluation, JSI, USA. [↑](#footnote-ref-11)
12. FHOK have recently undergone [↑](#footnote-ref-12)
13. Valadez, Joseph. Transgrud R, Mbugua M, and Smith T. 1997 *Assessing Family Planning Service Delivery Skills in Kenya*. Studies in Family Planning Vol 28 number 2 [↑](#footnote-ref-13)
14. Kenya now has two Ministries of Health: Medical Services are responsible for clinical services in hospitals and Public Health and Sanitation are responsible for preventive services in Health Centres [↑](#footnote-ref-14)
15. Kumaranayake et al (2000) Costing guidelines for HIV prevention strategy. UNAIDS, Geneva. [↑](#footnote-ref-15)
16. Robinson, W.S. (1951) The logical structure of analytic induction. *American Sociological Review* 16: 12-18. [↑](#footnote-ref-16)
17. Constructs will be developed with focus group participants during the pilot study [↑](#footnote-ref-17)
18. Constructs will be developed with focus group participants during the pilot study [↑](#footnote-ref-18)
