# Supplementary material for: Ambivalence in pregnancy intentions: The effect of quality of care and context among a cohort of women attending family planning clinics in Kenya
Source: PLoS One. 2018 Jan 9;13(1):e0190473. doi: 10.1371/journal.pone.0190473 (PMC5760043; doi:10.1371/journal.pone.0190473)
Supplement: S1 Fig — (DOCX) [file pone.0190473.s002.docx]

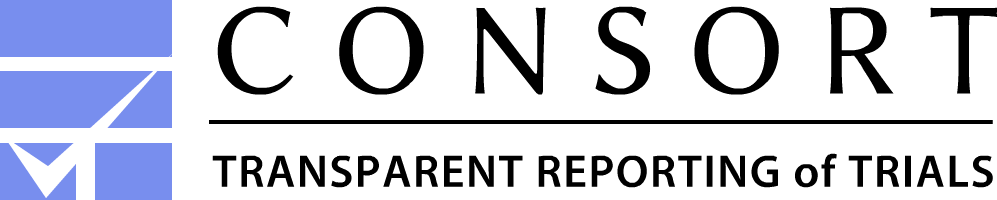


**S1 Figure: Participant Flow Diagram**

Analysed (**n=496** )
♦ Excluded from analysis (give reasons)(n=48) =Not interviewed in all four rounds

Lost to follow-up (give reasons) (n= 409 )

Migrated out of the study sites, refused or could not be traced after 3 attempts

## Follow-Up

## Analysis

Analysed (**n= 557** )
♦ Excluded from analysis (give reasons) (n=54) = Not interviewed in all four rounds

Lost to follow-up (give reasons) (n=393) Migrated out of the study sites, refused or could not be traced after 3 attempts

Allocated to intervention

(**Intervention Group)** (n=953)

## Allocation

Not allocated to intervention

(**Control Group)** (n=1004)

**Randomized (n=1957)**

## Enrollment

## Analysis (n= 1053)
