# Supplementary material for: Ambivalence in pregnancy intentions: The effect of quality of care and context among a cohort of women attending family planning clinics in Kenya
Source: PLoS One. 2018 Jan 9;13(1):e0190473. doi: 10.1371/journal.pone.0190473 (PMC5760043; doi:10.1371/journal.pone.0190473)
Supplement: S2 Table — (DOCX) [file pone.0190473.s004.docx]

**S2 Table: Annex 1: Attributes of service delivery**

| **Constructs used** | **Elements for each construct** |
| --- | --- |
| Building rapport adequately (0-5) | Providers greet client in a friendly manner |
|  | Provider used clients name |
|  | Encourage client to ask question |
|  | Ensured privacy |
|  | Provider assured about confidentiality |
| Adequate history taking (0-4) | Asked about HIV sero status |
|  | Asked about date of last menses |
|  | Asked age of youngest child |
|  | Took history of medical condition |
| Use of IEC materials adequately (0-3) | Used BSC+ cards |
|  | Provider used contraceptive samples |
|  | Provider used anatomical models |
| Adequate counselling on method use (0-3) | Providers discussed how to use method |
|  | Providers discussed benefit of methods |
|  | Provider discusses possible side effects |
| Overall STI management (0-10) | Discussed STI with client |
|  | Discussed HIV with client |
|  | Discussed STI risk factors with client |
|  | Discussed multiple partners as risk factor |
|  | Discussed that STI increase risks of HIV |
|  | Discussed unprotected sexual intercourse as a risk factor |
|  | Discussed not knowing partner status as risk factor |
|  | give information on symptoms of STI |
|  | Screened for STI |
|  | Advice to seek medical attention if notice symptoms |
| Adequate documentation (0-2) | looked at client's health card before beginning the consultation |
|  | recorded information in register/ tally sheet |
| Total scores (0-27) |  |
